# Butyrophilin 3A1 Contributes to Inflammation and Induces a Lupus‐Like Disease by Inhibiting the IL‐38‐Ferroptosis Axis

**DOI:** 10.1002/mco2.70356

**Published:** 2025-09-14

**Authors:** Wang‐Dong Xu, Da‐Cheng Wang, Yang‐Yang Tang, Qi Huang, Lu Fu, You‐Yue Chen, Lu‐Qi Yang, Si‐Yu Feng, Lin‐Chong Su, An‐Fang Huang

**Affiliations:** ^1^ Department of Evidence‐Based Medicine, School of Public Health Southwest Medical University Luzhou Sichuan China; ^2^ Vanke School of Public Health Tsinghua University Beijing China; ^3^ Laboratory Animal Center Southwest Medical University Luzhou Sichuan China; ^4^ Hubei Provincial Key Laboratory of Occurrence and Intervention of Rheumatic Diseases Affiliated Minda Hospital of Hubei Minzu University Enshi Hubei China; ^5^ Department of Rheumatology and Immunology Affiliated Minda Hospital of Hubei Minzu University Enshi Hubei China; ^6^ Department of Rheumatology and Immunology the Affiliated Hospital Southwest Medical University Luzhou Sichuan China

**Keywords:** butyrophilin 3A1, IL‐38, inflammation, lupus

## Abstract

Systemic lupus erythematosus (SLE) is an autoimmune disease of unknown origin. Recent evidence has linked butyrophilin 3A1 (BTN3A1) to immune dysregulation. This study was to elucidate the relationship of BTN3A1 in SLE. Expression of BTN3A1 in plasma and peripheral blood mononuclear cells from SLE patients and healthy controls explored the association between BTN3A1 and SLE. We found that BTN3A1 mRNA, plasma levels, and expression in CD4^+^ T cells were significantly elevated in SLE patients. In BTN3A1 gene knock‐in (BTN3A1^KI^) mice, inflammation and lupus‐like manifestations occurred, including increased proportions of Th1, Th2, and Th17 cells, decreased Treg cells, elevated levels of inflammatory cytokines and anti‐dsDNA antibodies, renal injury, and suppressed IL‐38 serum levels. Intraperitoneal injection of IL‐38 in pristane‐treated BTN3A1^KI^ mice notably alleviated these pathological changes. Mechanistic investigations revealed that CD4^+^ T cells and the ferroptosis pathway were closely associated with the effects mediated by the BTN3A1‐IL‐38 axis. In vitro experiments showed that IL‐38 stimulation reduced proliferation, apoptosis, and decreased the expression of ferroptosis‐related proteins, Fe^2^⁺, glutathione, and malondialdehyde in CD4^+^BTN3A1^+/+^ T and BTN3A1^+/+^ Jurkat T cells. Overall, BTN3A1 plays a crucial role in SLE pathogenesis by regulating CD4^+^ T cell function.

## Graphical abstract

We found that BTN3A1 was abnormally expressed in peripheral blood of SLE patients. BTN3A1 promotes the release of inflammatory factors and antibodies in mice and the pathological damage of the kidney, and multi‐omics reveal the abnormalities of CD4 + T cells and the occurrence of ferroptosis. Finally, we demonstrated that BTN3A1 promotes inflammation and induces lupus‐like diseases by inhibiting the IL‐38‐iron death axis through BTN3A1.

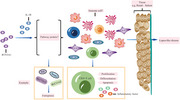

## 1 Introduction

Systemic lupus erythematosus (SLE) is a complex autoimmune disease, and its main causes may include immune dysregulation, genetics, and environmental irritation. SLE is highly prevalent in women often attacks systemic organs, including the kidneys, blood, joints, and nervous system. During the development of SLE, CD4^+^ T cells, the master population of a class of αβ T cells, are hyperactivated, leading to loss of immune tolerance. In addition, autoantibodies are produced by memory B cells or plasma cells whose differentiation is induced by CD4^+^ T helper (Th) cells [1]. The imbalance of CD4^+^ T cells, especially Th17/Treg, is a key feature of disease activity in SLE, and there is activated CD4^+^ T cell infiltration in the affected kidney, skin, and other organs [2, 3]. Studies have confirmed that the occurrence of ferroptosis in CD4^+^ T cells has an important impact on the immune imbalance of lupus nephritis [4]. Thus, the balance of CD4^+^ T cells is critical to the pathogenesis of SLE. Moreover, searching for biomarkers regulating CD4^+^ T cell function and discussing how the potential biomarkers are involved in CD4^+^ T cell signaling events could provide new therapeutic options for SLE treatment.

The Butyrophilin 3A (BTN3A, also called CD277) subfamily, consisting of BTN3A1, BTN3A2, and BTN3A3, plays a key role in regulating immunity [5]. BTN3A receptors expressed on antigen‐presenting cells (APCs) can promote negative co‐stimulatory functions upon binding to ligands expressed on T cells. It has been demonstrated that BTN3A1 is significantly associated with T cell function in the tumor microenvironment [6, 7]. *BTN3A1*, a major histocompatibility complex‐associated gene, is located on the short arm of human chromosome 6 (6p22.1). It encodes a membrane protein with two extracellular immunoglobulin structural domains and an intracellular B30.2 structural domain [8]. The positively charged pocket formed by the B30.2 structural domain binds directly to a range of negatively charged small molecules, such as the associated phosphoantigen (pAg) [9]. BTN3A1 is expressed on the surface of some immune cell subpopulations, such as basophils, γδ T cells, memory CD8^+^ T cells, CD4^+^ T cells, and B cells [10]. In addition, expression of BTN3A1 was significantly positively correlated with infiltration of CD4^+^ T cells in gliomas [11]. Vγ9Vδ^2+^ T cells, as the main subset of γδ T cells in human peripheral blood, are involved in immune responses in infections, tumors, and inflammation, and their function is associated with BTN3A1 [12, 13]. The activation of BTN3A1 significantly enhances TCR‐induced T cell proliferation and the secretion of cytokines, and BTN3A1 plays a key role in the type I IFN response via the enhancement of interferon regulatory factor 3 (IRF3) phosphorylation and IFN‐γ secretion [14, 15].

BTN3A1 is closely associated with cancers, and its expression is strongly correlated with the clinical prognosis of cancers. For example, BTN3A1 is associated with tumor‐infiltrating immune cells and is co‐expressed with a variety of immune checkpoints in patients with breast cancer (BRCA) and non‐small cell lung cancer (NSCLC). Currently, there are limited studies on BTN3A1 and autoimmune diseases. Psoriasis patients had higher BTN3A1 expression in monocytes relative to the healthy subjects, with high expression of this factor in CD14^+^ cells of skin lesions [16]. A study discussed the rs3802604 polymorphism of *BTN3A1* gene, indicating that this locus is a risk polymorphism for type 1 diabetes [17]. In addition, our previous study has shown that the gene polymorphism of BTN3A1 is associated with the risk of SLE in the Chinese Han population [18]. Interleukin‐38 (IL‐38) has been shown to inhibit inflammation. For instance, IL‐38 inhibits Th17 cells and myeloid cells and then binds to IL‐1R6 to repress inflammation. THP‐1 monocyte cell line expresses low expression of IL‐6, TNF‐α, IL‐1β, IL‐17, and IL‐23 after IL‐38 stimulation [19]. In addition, IL‐38 has been shown to diminish the accumulation of eosinophils and the number of Th2 and Th17 cells, and to enhance the proportion of Treg cells [20]. Our previous studies showed that IL‐38 can alleviate liver, spleen, and kidney enlargement in lupus mice, reverse lupus‐like pathological changes, improve immune homeostasis imbalance, and inhibit the occurrence and development of lupus through regulating ERK1/2, JNK1/2, p38, NF‐κB p65, and STAT5 signaling pathways [21]. However, it remains to be elucidated whether BTN3A1 is involved in the pathogenesis of autoimmune diseases such as SLE by regulating IL‐38.

While BTN3A1 has been extensively investigated in cancers and γδ T cells, studies in CD4^+^ T cells and SLE are absent. The association between BTN3A1 and SLE pathogenesis remains to be discussed. In our study, we initially explored the expression of BTN3A1 in the peripheral blood of SLE patients, as well as the expression of BTN3A1 in various immune cells. Since the *BTN3A1* gene is not expressed in mouse, next, we knock in the *BTN3A1* gene into wild‐type (WT) C57BL/6 mice (BTN3A1^KI^) to clarify the role of BTN3A1 in inflammation and SLE occurrence. Moreover, our previous findings showed abnormal expression of IL‐38 in SLE patients, and IL‐38 inhibited lupus development. We then investigated the association of BTN3A1 and IL‐38, and discussed whether IL‐38 is involved in the BTN3A1‐mediated effects. These results highlight the implication of BTN3A1 for inflammation and SLE development, and suggest a novel mechanism by which BTN3A1 is involved in ferroptosis of CD4^+^ T cells through inhibition of IL‐38.

## 2 Results

### 2.1 Highly Expressed BTN3A1 in SLE Patients

**FIGURE 1:**
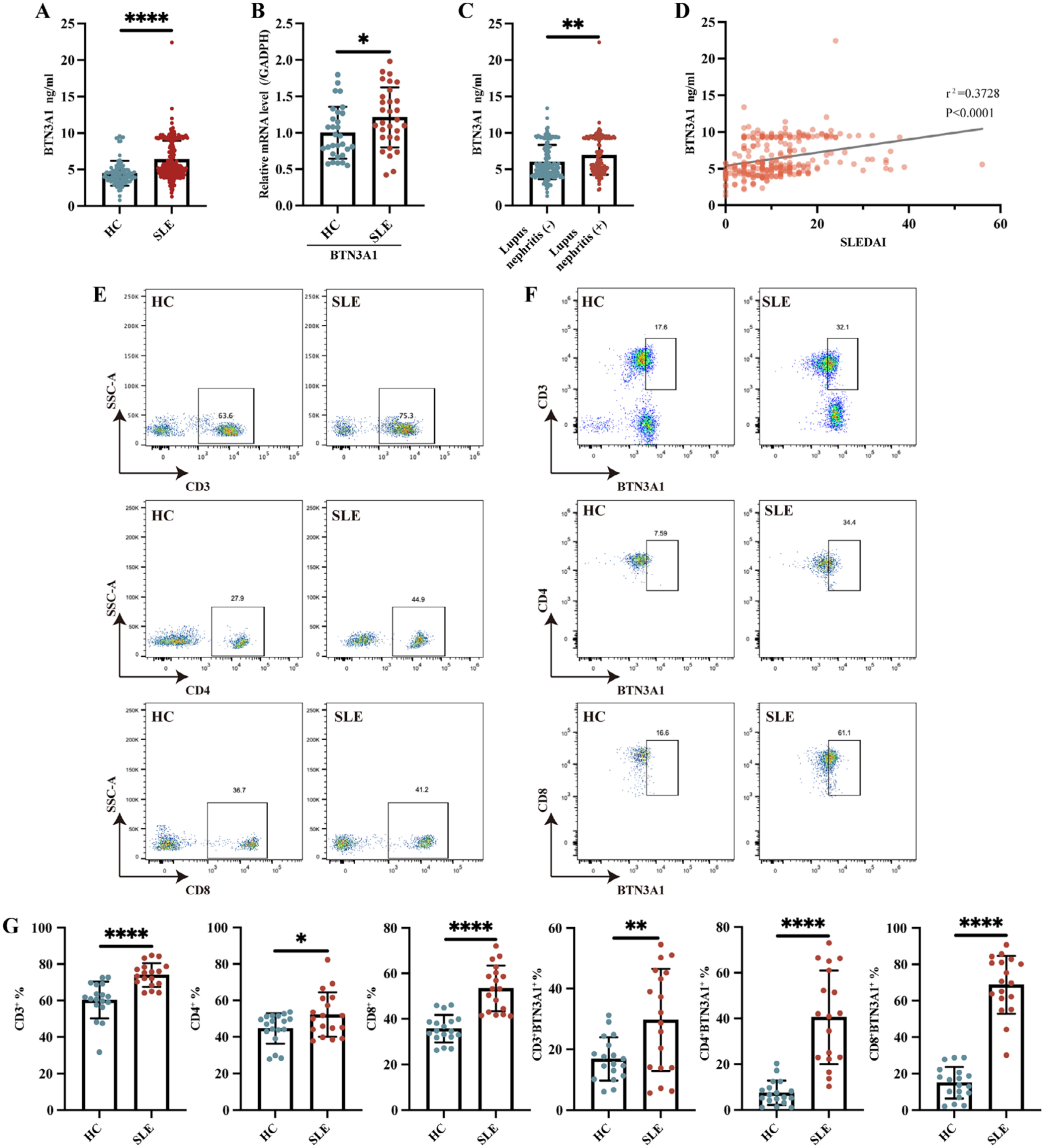
BTN3A1 expression in systemic lupus erythematosus patients (SLE). (A) Plasma levels of BTN3A1 from healthy controls (*N* = 100) and SLE patients (*N* = 280) were detected by ELISA. (B) Detection of mRNA expression of BTN3A1 in healthy controls (*N* = 30) and SLE patients (*N* = 30) by qRT‐PCR. (C) Plasma levels of BTN3A1 in SLE patients with lupus nephritis (*N* = 123) and without lupus nephritis (*N* = 157). (D) Relationship between BTN3A1 plasma levels and disease activity index score in SLE patients. (E, F) Representative flowchart of different T cells and BTN3A1 expression in different T cells from healthy controls (*N* = 18) and SLE patients (*N* = 18). (G) Statistical analysis of T cell subpopulations and BTN3A1 expression in different T cell subpopulations. *p < 0.05, **p < 0.01, and ****p < 0.0001.

To investigate whether expression of BTN3A1 was abnormal in SLE patients and whether expression of BTN3A1 was related to SLE pathogenesis, we examined plasma levels of BTN3A1 and BTN3A1 mRNA expression from PBMCs in SLE patients. Plasma levels of BTN3A1 were significantly increased in SLE patients compared with healthy controls (Figure 1A), and BTN3A1 mRNA expression was significantly increased in SLE patients compared with healthy controls (Figure 1B). There were higher plasma levels of BTN3A1 in SLE patients with lupus nephritis compared to patients without lupus nephritis (Figure 1C). The plasma levels of BTN3A1 were positively correlated with SLE disease activity index (SLEDAI) score (*r* ^2^ = 0.3728, *p* < 0.0001, Figure 1D). Subgroup analysis showed that plasma levels of BTN3A1 were significantly related to several clinical and laboratory features, such as vasculitis, arthritis, cylindruria (Figure S1A–H), and were significantly correlated with C3, RF expression (Figure S1I,J). Interestingly, the proportion of CD3^+^, CD4^+^, CD8^+^ T cells was higher in SLE patients compared with healthy controls (Figures 1E–G). The proportion of CD3^+^BTN3A1^+^, CD4^+^BTN3A1^+^, CD8^+^BTN3A1^+^ T cells was also higher in SLE patients (Figures 1E–G). These were similar to CD19^+^, CD14^+^, CD11b^+^, CD11c^+^ cells, where the proportion of CD19^+^, CD14^+^, CD11b^+^, CD11c^+^ cells was higher in SLE patients (Figure S2A), and proportion of CD19^+^BTN3A1^+^, CD14^+^BTN3A1^+^, CD11b^+^BTN3A1^+^, CD11c^+^BTN3A1^+^ cells was significantly higher in SLE patients compared with healthy controls, respectively (Figure S2B). The findings suggest that expression of BTN3A1 was highly expressed in SLE patients, and may correlate with SLE risk.

### 2.2 Knock‐in BTN3A1 Promotes Inflammation and Induces a Lupus‐Like Disease

**FIGURE 2:**
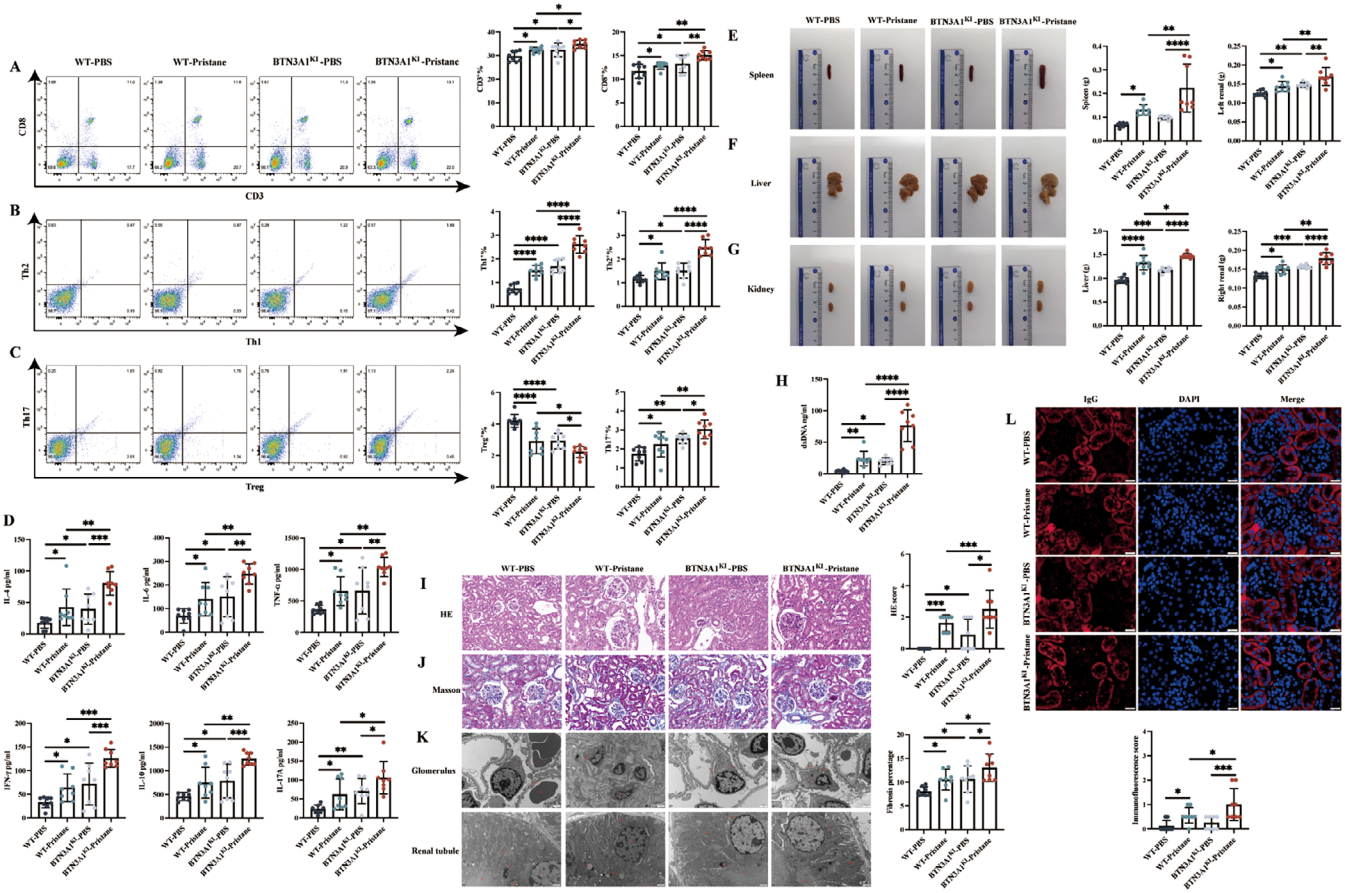
BTN3A1 promotes inflammation and induces lupus‐like features. (A–C) Representative flowchart and statistical analysis of CD3^+^, CD8^+^ T cells, Th1, Th2, Th17, Treg cells in mice from the wild‐type (WT)‐PBS (*N* = 8), WT‐Pristane (*N* = 8), BTN3A1^KI^‐PBS (*N* = 8) and BTN3A1^KI^‐Pristane groups (*N* = 8). (D) Inflammatory cytokines IL‐4, IL‐6, TNF‐α, IFN‐γ, IL‐10 and IL‐17A were detected by CBA kits in the serum of different groups of mice. (E–G) Representative figure and statistical analysis of mice spleen, liver and kidneys. (H) The dsDNA levels were detected by ELISA. (I, J) HE staining, Masson staining and statistical analysis of mice kidneys showed the extent of kidney damages and fibrosis. (K) Ultrastructure of glomerular and renal tubular damages in mice as revealed by transmission electron microscope (TEM). (L) Representative figure of IgG deposition in mice kidneys assessed by immunofluorescence intensity and statistical analysis. *p < 0.05, **p < 0.01, ***p < 0.001 and ****p < 0.0001.

Since the *BTN3A1* gene does not exist in WT mice, we constructed the human‐derived *BTN3A1* gene knock‐in (BTN3A1^KI^) mice (Figure S3A,B). First, we discussed whether the knock‐in *BTN3A1* gene may contribute to inflammation. Second, we evaluated whether the knock‐in *BTN3A1* gene may induce a lupus‐like disease (Figure S3C). As shown in Figure 2A–C and Figure S4A,B, the BTN3A1^KI^ mice injected with PBS had an upregulated proportion of CD3^+^, CD8^+^, Th1, Th2, Th17 cells, CD19^+^, CD14^+^, CD11b^+^, CD11c^+^ cells, and a downregulated proportion of Treg cells compared with the WT mice injected with PBS. Expression of inflammatory cytokines IL‐4, IL‐6, IL‐10, IL‐17A, IFN‐γ, and TNF‐α was increased in the BTN3A1^KI^‐PBS group as well (Figure 2D). After pristane injection, mice in the BTN3A1^KI^‐Pristane group showed a higher proportion of CD3^+^, CD8^+^, Th1, Th2, Th17 cells, CD19^+^, CD14^+^, CD11b^+^, CD11c^+^ cells, and a lower proportion of Treg cells compared with the WT‐Pristane group (Figure 2A–C and Figure S4A,B). Expression of the inflammatory cytokines (IL‐4, IL‐6, IL‐10, IL‐17A, IFN‐γ, and TNF‐α) was increased in the BTN3A1^KI^‐Pristane group (Figure 2D). Thus, the knock‐in *BTN3A1* gene contributed to inflammation.

We further discussed the role of the knock‐in *BTN3A1* gene in lupus development. Morphologically, the BTN3A1^KI^‐PBS group displayed significant splenomegaly, hepatomegaly, and bilateral renal enlargement (Figures 2E–G). Organ weight analysis revealed that the liver and kidneys of BTN3A1^KI^‐PBS mice were notably heavier than those of the WT‐PBS group. In addition, white spherical calcified particles were observed adhering to the hepatic lobules, further highlighting the pathological changes. These signs of splenomegaly, hepatomegaly, and renal enlargement were more pronounced in the BTN3A1^KI^‐Pristane group (Figures 2E–G). In terms of autoantibody expression, the BTN3A1^KI^‐PBS group exhibited a marked increase in dsDNA autoantibody levels compared to the WT‐PBS group, with BTN3A1^KI^ lupus mice showing the highest dsDNA expression (Figure 2H). Histopathological examination of the kidneys demonstrated that the BTN3A1^KI^‐PBS group had a significantly higher HE score and fibrosis percentage than the WT‐PBS group (Figures 2I,J). Microscopic analysis revealed glomerular segmental or global sclerosis, thickening of the renal capsule basement membrane, epithelial nuclear swelling and hyperplasia, and nephrogenic fibrosis. Transmission electron microscopy (TEM) further confirmed severe damage to the glomeruli and tubular structures in BTN3A1^KI^‐PBS mice (Figure 2K). Compared with the WT‐Pristane group, the BTN3A1^KI^‐Pristane group presented more severe renal injury (Figures 2I–K), accompanied by increased IgG deposits (Figure 2L). Collectively, these findings indicate that the *BTN3A1* gene knock‐in exacerbates inflammation and elicits lupus‐like manifestations.

### 2.3 BTN3A1 Induces the Lupus Development by Inhibiting IL‐38 Expression

**FIGURE 3:**
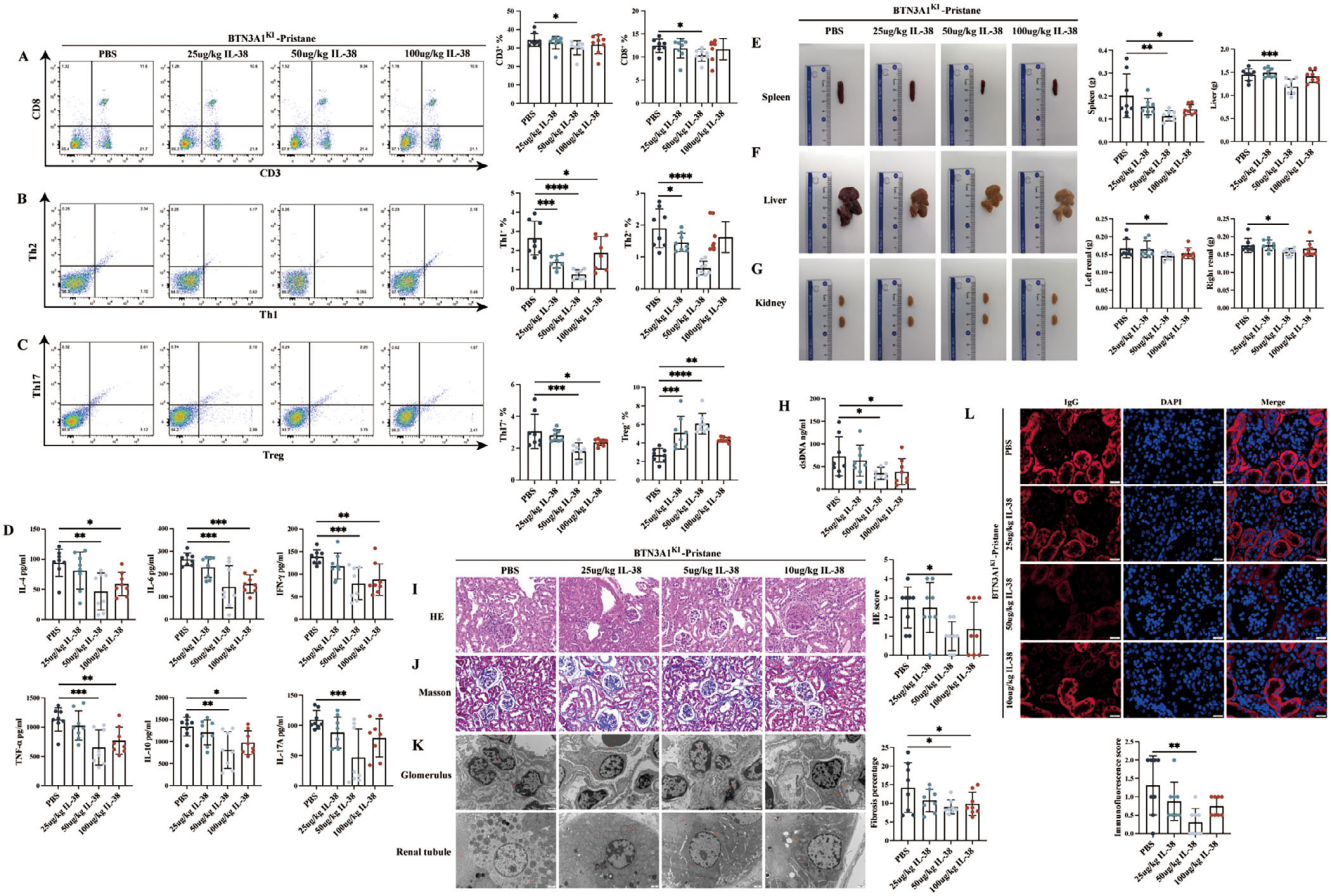
IL‐38 alleviates BTN3A1‐induced inflammation and the lupus‐like disease. (A–C) Representative flowchart and statistical analysis of CD3^+^, CD8^+^ T cells, Th1, Th2, Th17, Treg cells in mice from the BTN3A1^KI^‐Pristane‐PBS group (*N* = 8) and BTN3A1^KI^‐Pristane‐IL‐38 groups (with injection of different concentrations of IL‐38, *N* = 8/group). (D) The inflammatory cytokines IL‐4, IL‐6, TNF‐α, IFN‐γ, IL‐10 and IL‐17A were detected by the CBA kits in the serum from different groups of mice. (E–G) Representative figure and statistical analysis of mice spleen, liver, and kidneys. (H) The dsDNA levels in different groups of mice were detected by ELISA. (I, J) HE staining, Masson staining and statistical analysis of mice kidneys showed the extent of kidney damages and fibrosis. (K) Ultrastructure of glomerular and renal tubular damages in mice as revealed by TEM. (L) Representative figure of IgG deposition in mice kidneys assessed by immunofluorescence intensity and statistical analysis. *p < 0.05, **p < 0.01, ***p < 0.001 and ****p < 0.0001.

In our previous studies, we found higher plasma levels of IL‐38 and higher IL‐38 mRNA expression in PBMCs from SLE patients compared with healthy controls [21], and IL‐38 inhibited lupus development [22]. To discuss whether BTN3A1 may regulate IL‐38 expression, and then promote inflammation and induce the lupus‐like disease, mice in the BTN3A1^KI^‐Pristane group were injected with recombinant mouse IL‐38. First, we validated expression of IL‐38 in plasma and PBMCs from SLE patients, showing consistent results with previous findings (Figure S5A,B). Serum levels of IL‐38 were examined in the WT‐PBS, WT‐Pristane, BTN3A1^KI^‐PBS, and BTN3A1^KI^‐Pristane groups, and serum IL‐38 levels were lower in the BTN3A1^KI^‐Pristane group and BTN3A1^KI^‐PBS group than in the WT‐Pristane group and WT‐PBS group, respectively (Figure S6). This suggests that BTN3A1 inhibited IL‐38 expression in the mice with lupus‐like disease. Second, mice in the BTN3A1^KI^‐Pristane group were injected with different doses of recombinant mouse IL‐38. Proportion of inflammation‐related immune cells and expression of inflammatory cytokines were reversed by addition of IL‐38, evidenced by less proportion of CD3^+^, CD8^+^, Th1, Th2, Th17 cells, CD19^+^, CD14^+^, CD11b^+^, CD11c^+^ cells, more proportion of Treg cells, lower expression of the inflammatory cytokines (IL‐4, IL‐6, IL‐10, IL‐17A, IFN‐γ, and TNF‐α) in the BTN3A1^KI^‐Pristane‐IL‐38 group (Figures 3A–D, Figure S7A,B). Splenomegaly, hepatomegaly, and swelling of both kidneys was reversed in the BTN3A1^KI^‐Pristane‐IL‐38 group, along with less weight of the above organs (Figures 3E–G). Serum levels of autoantibody dsDNA were reduced in the BTN3A1^KI^‐Pristane‐IL‐38 group (Figure 3H). Interestingly, histopathology of the kidneys was improved in the BTN3A1^KI^‐Pristane‐IL‐38 group, evidenced by lower HE score, fibrosis percentage, and less structural damage in the kidneys (Figures 3I–K). IgG deposition in the kidneys was inhibited in the BTN3A1^KI^‐Pristane‐IL‐38 group (Figure 3L). Together, BTN3A1 inhibited IL‐38 expression and then contributed to inflammation and induced the lupus‐like disease.

### 2.4 Ferroptosis Is a Key Signaling Pathway in the BTN3A1‐IL‐38 Axis‐Mediated Effects

**FIGURE 4:**
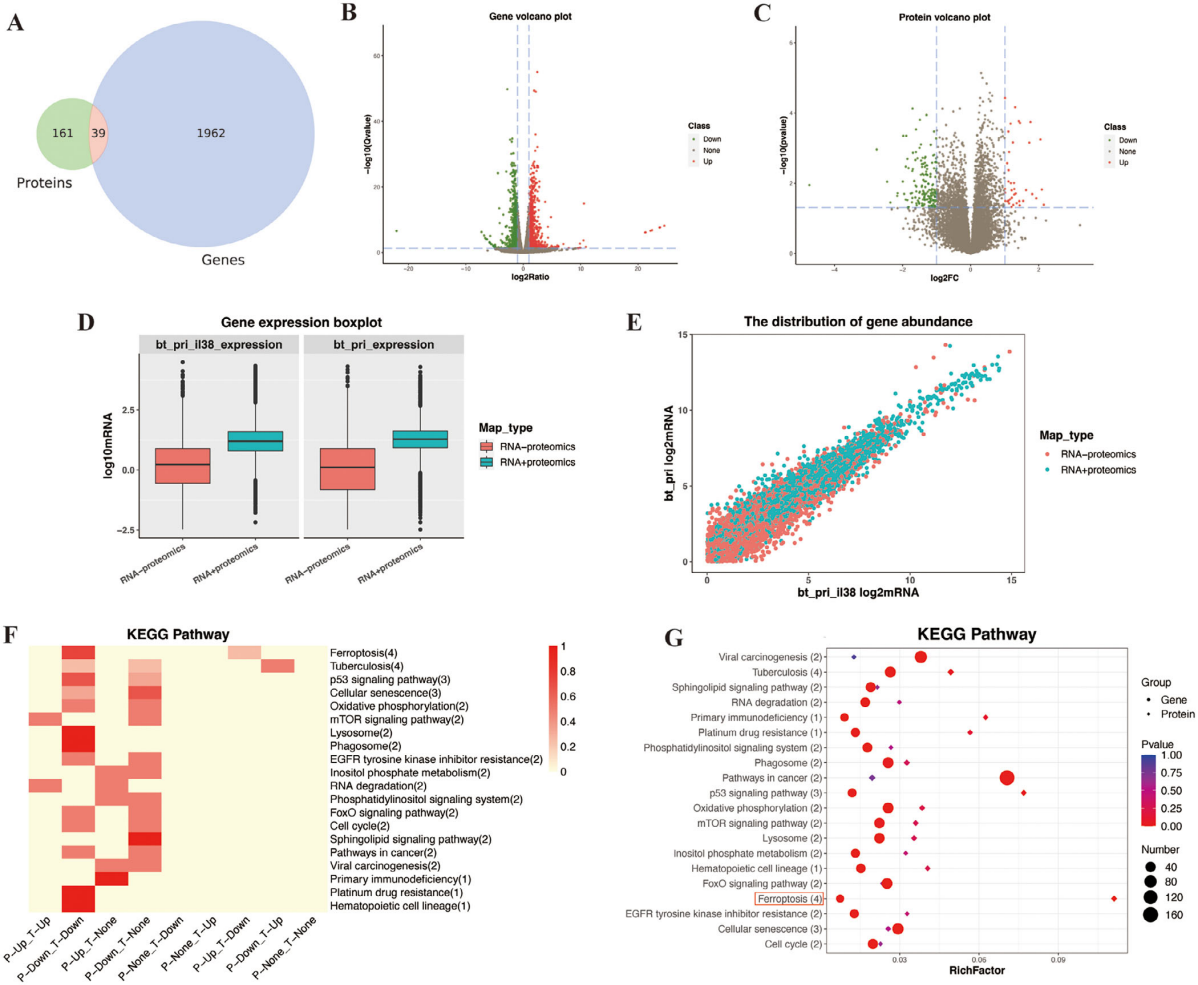
Transcriptomics and proteomics cross‐analysis explores signaling pathways regulated by the BTN3A1‐IL‐38 axis. (A) Overlap of differentially expressed proteins and differentially expressed genes in mice from the BTN3A1^KI^‐Pristane‐PBS group (*N* = 3) and the BTN3A1^KI^‐Pristane‐IL‐38 group (with injection of recombinant IL‐38 (50ug/kg), *N* = 3) (featured by Venn diagrams). (B, C) All differentially expressed genes and differentially expressed proteins visualized by Volcano plots. (D, E) Box plots and scatter plots visualized the abundance of genes associated with proteins in the BTN3A1^KI^‐Pristane‐PBS group and the BTN3A1^KI^‐Pristane‐IL‐38 groups. Red represents the distribution of gene abundance in transcriptomics associated to proteomics. Green represents the distribution of gene abundance in transcriptomics not associated to proteomics. (F, G) KEGG pathway enrichment showed key pathways, and KEGG enrichment heat map showed distribution of key genes and key proteins.

As discussed above, BTN3A1 contributed to inflammation and induced the lupus‐like disease by inhibiting IL‐38. We further discussed the mechanisms that the BTN3A1‐IL‐38 axis involves. Spleens of the different groups of mice were evaluated by transcriptomics and proteomics analysis. The number of differentially expressed proteins and genes was summarized in Table S1 when different group was compared with each other. Transcriptomics and proteomics correlation analysis showed that among the 200 differentially expressed proteins and 2001 differentially expressed genes, 39 key genes/proteins were shown by Venn diagrams (Figure 4A). The upregulation and downregulation of differentially expressed genes and proteins were shown in the Volcano plot (Figure 4B,C). Based on the transcriptomics data, the genes and proteins were categorized as RNA‐proteomics and RNA+proteomics, and the distribution of gene expression and gene abundance in the two subgroups was shown separately. There was higher expression and abundance of RNA+proteomics genes after association compared with RNA‐proteomics genes (Figures 4D,E). We subjected 39 key genes/proteins to KEGG pathway analysis, of which four key genes were enriched in ferroptosis, including downregulated Slc11a2, Steap3, and Tfrc in both transcriptomics and proteomics analysis, as well as downregulated Slc7a11 in transcriptomics and upregulated Slc7a11 in proteomics analysis (Figure 4F, Table S2). Ferroptosis was significantly different in KEGG pathway enrichment in both transcriptomics and proteomics analysis (Figure 4G, Table S2). Results of the other comparisons were shown in Figures S8–S10, Table S3.

### 2.5 T Cells Are Significantly Related to the BTN3A1 Inhibition of IL‐38

**FIGURE 5:**
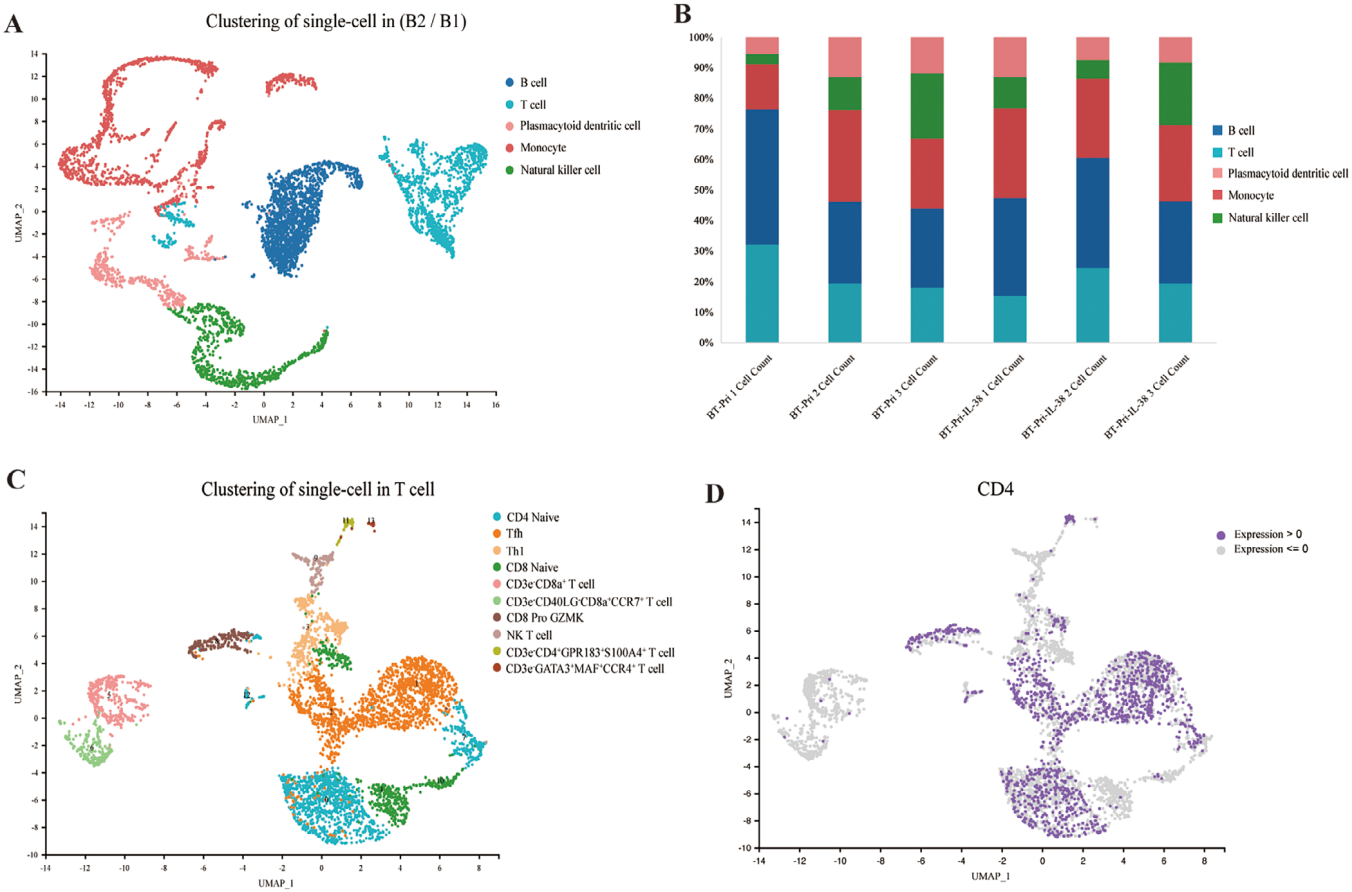
Single‐cell sequencing in mice from the BTN3A1^KI^‐Pristane‐PBS group and the BTN3A1^KI^‐Pristane‐IL‐38 group (50ug/kg IL‐38) showed significantly different T cells distribution. (A) UMAP plot showed the 5 major cell types identified based on marker genes in mice spleen tissues (*N* = 3/group). (B) Composite bar graphs showed percentages of T cells, B cells, natural killer cells, monocytes and pDCs in each sample. (C, D) T cells were clustered again, and the UMAP plot showed the results of distribution of all T cell subpopulations, as well as the expression of CD4 in these cluster species.

To better clarify which immune cells were regulated by the BTN3A1‐IL‐38 axis, single‐cell transcriptomics explored the differentially distributed immune cells. After removing damaged and duplicated cells from the data, we retained 47067 high‐quality cellular transcriptomes for further analysis. For different immune cells, we used the marker genes identified for the corresponding cells. The relevant marker genes and their sources are shown in Table S4. We used principal component analysis (PCA) and cluster analysis for the determination of cellular lineages. Comparing the immune cells in the BTN3A1^KI^‐Pristane‐PBS group and the BTN3A1^KI^‐Pristane‐IL‐38 group (injected with 50ug/kg recombinant mouse IL‐38), five cell populations were identified, namely T cells, B cells, monocytes, natural killer (NK) cells, and plasmacytoid dendritic (pDC) cells (Figure 5A). The distribution of these five cell populations in each sample was shown by composite bar graphs, and there is a significant difference in the distribution of T cells between the two groups (Figure 5B, Table S5). Therefore, we performed cluster analysis again for the T cells, and 10 T cell subpopulations were identified. It is notable that CD4 was the most expressed marker in these cells (Figures 5C,D; Table S6). Then, we explored the CD4^+^ T cells in further experiments. In addition, Slc11a2, Steap3, Tfrc, and Sle7a11 are presently expressed in T cells (Figure S11). For the other immune cells (B cells, NK cells, monocytes, pDC), we all performed clustering again, and the corresponding cell subpopulations and cell numbers were shown in the appendix (Figures S12–S15, Tables S7–S10).

### 2.6 BTN3A1 Inhibits the IL‐38‐Ferroptosis Pathway and Then Promotes T Cell Dysfunction

We transfected WT C57BL/6 mice with BTN3A1^+/+^ adenovirus and isolated naïve CD4^+^ T cells for the following analysis. Similarly, Jurkat T cells were infected with BTN3A1^+/+^ adenovirus. As shown in Figure S16A,B, infection of Jurkat T cells with the BTN3A1^+/+^ adenovirus (MOI = 200, Day 6) and injection of BTN3A1^+/+^ adenovirus (10^9^ PFU, Day 5) in WT mice showed the optimal expression of BTN3A1, respectively. Moreover, 100 ng/mL of IL‐38 stimulation showed the optimal effects on proliferation and apoptosis of CD4^+^ T cells (Figure S16C–E).

**FIGURE 6:**
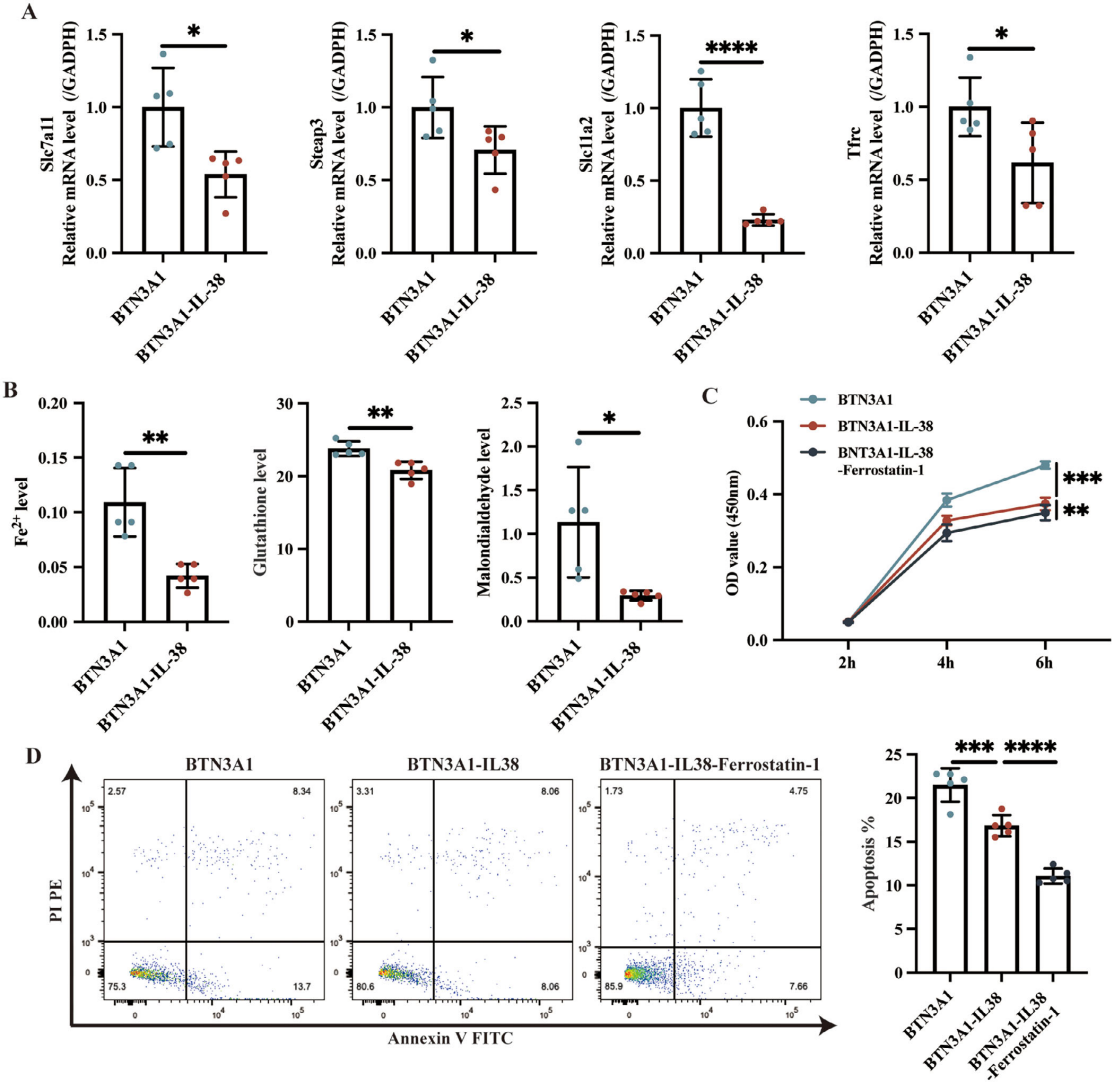
BTN3A1 inhibits the IL‐38‐ferroptosis axis and induces CD4^+^ T cells dysfunction. Naïve CD4^+^ T cells were isolated from spleens of the BTN3A1^+/+^ mice (*N* = 5/group), and then were activated. Part of the cells were further stimulated with 100 ng/mL of IL‐38. (A) The mRNA expression of ferroptosis‐related proteins in the cells was detected by qRT‐PCR. (B) Detection of Fe^2+^, glutathione and malondialdehyde levels in above cells reflects cellular ferroptosis. (C, D) The IL‐38‐treated CD4^+^BTN3A1^+/+^ T cells we intervened with/without Ferrostatin‐1 treatment. Cell proliferation was measured by CCK8 kits and cell apoptosis was measured. Representative flowchart and statistical analysis of apoptosis of different groups of cells are shown. *p < 0.05, **p < 0.01, ***p < 0.001 and ****p < 0.0001.

In CD4^+^BTN3A1^+/+^ T cells, the mRNA expression of ferroptosis‐related proteins (Scl7a11, Steap3, Slc11a2, Tfrc; Figure 6A) was significantly decreased after the addition of 100 ng/mL IL‐38. Similarly, 100 ng/mL IL‐38 stimulation was able to significantly inhibit the ferroptosis pathway, by which the cellular levels of Fe^2+^, glutathione, and malondialdehyde were significantly decreased (Figure 6B), suggesting that BTN3A1 inhibits IL‐38 and then regulates activation of the ferroptosis pathway. Moreover, CD4^+^BTN3A1^+/+^ T cells were treated with IL‐38 or IL‐38+Ferrostatin‐1 (an inhibitor of the ferroptosis pathway). Compared with CD4^+^BTN3A1^+/+^ T cells (Control group/Blank), addition of 100 ng/mL IL‐38 stimulation inhibited apoptosis and proliferation of the cells, which were further significantly reduced in the presence of Ferrostatin‐1 (Figure 6C,D).

In addition, the Jurkat T cells were used to validate the above mechanisms (Figure S17A–F). The results showed that the proliferation and apoptosis of BTN3A1^+/+^ Jurkat T cells were inhibited by 100 ng/mL IL‐38 stimulation (Figure S17A). Jurkat T cells transfected with BTN3A1^+/+^ adenovirus in the presence of 100 ng/mL IL‐38 had significantly reduced mRNA expression of ferroptosis‐related proteins Slc11a2, Steap3, Slc7a11, and Tfrc (Figure S17B), and cellular levels of Fe^2+^, glutathione, and malondialdehyde (Figure S17C). Moreover, the addition of Ferrostatin‐1 to IL‐38‐treated BTN3A1^+/+^ Jurkat T cells had less cell proliferation and apoptosis compared with IL‐38‐treated BTN3A1^+/+^ Jurkat T cells without Ferrostatin‐1 treatment (Figure S17D,E). Furthermore, BTN3A1 can promote ferroptosis, evidenced by increased expression of ferroptosis‐related proteins, which in turn lead to proliferation and apoptosis of Jurkat cells (Figure S17B–F). Thus, BTN3A1 not only directly promotes ferroptosis but also indirectly promotes ferroptosis by inhibiting the IL‐38 signaling. Together, BTN3A1 suppresses the IL‐38‐ferroptosis pathway and then contributes to T cell proliferation and apoptosis.

## 3 Discussion

In this study, we found that highly expressed BTN3A1 in SLE patients was significantly associated with severe clinical manifestations, such as lupus nephritis, and correlated with SLEDAI score. BTN3A1^KI^ mice showed inflammation and the lupus‐like inflammatory manifestations, including renal injuries, imbalance of T cell subpopulations, a burst of inflammatory cytokines, and decreased levels of serum IL‐38. In addition, BTN3A1 contributes to CD4^+^ T cells' proliferation and apoptosis by inhibiting the IL‐38‐ferroptosis signaling pathway. Collectively, BTN3A1 contributes to inflammation and induces the lupus‐like disease.

BTN3A1 has been widely studied in tumors and is abundantly expressed in the microenvironment of human epithelial ovarian cancer and BRCA [23]. Hypoxia‐stimulated dendritic cells revealed high expression of BTN3A1 [7]. Our study found that plasma levels of BTN3A1 and BTN3A1 mRNA expression in PBMCs were elevated in SLE patients. Similarly, BTN3A1 expression was elevated in different immune cells from SLE patients, including CD3^+^, CD4^+^, CD8^+^ T cells. CD14^+^ cells in psoriatic skin lesions highly expressed BTN3A1 and were regulated by IFN‐γ [16]. We also found highly expressed BTN3A1 in CD14^+^ cells from SLE patients. These data provide supportive evidence that BTN3A1 may be involved in the pathogenesis of SLE. To validate this hypothesis, BTN3A1^KI^ mice were constructed, and the lupus‐like disease was induced by pristane injection. We examined inflammatory immune cells and cytokines in the physiological status of BTN3A1^KI^ mice, suggesting an increased percentage of CD3^+^, CD8^+^ T cells, Th1, Th2, Th17 cells, and decreased Treg cells, as well as higher expression of IL‐4, IL‐6, IL‐10, IL‐17A, IFN‐γ, and TNF‐α. These inflammatory phenotypes were further exacerbated in the lupus‐like mice. It is accepted that an imbalance of Th1, Th2, Th17, Treg cells is the basis of the pathogenesis of SLE [24]. Therefore, BTN3A1 may contribute to T cell dysfunction, leading to inflammation. The kidneys are the most vulnerable organ in SLE [25]. Renal pathological analysis and TEM findings revealed renal structural damage in pristane‐induced lupus mice. Indeed, BTN3A1^KI^ mice were able to express the human‐derived *BTN3A1* gene [26], and the mouse model of lupus induced by pristane was able to develop dysregulated immune milieu and systemic organs damage similar to human SLE [27]. Thus, BTN3A1 can induce the lupus‐like disease.

IL‐38 demonstrates potent anti‐inflammatory effects in SLE [21]. IL‐38 competitively binds to IL‐36R, IL‐1R1, IL‐1 receptor accessory protein‐like‐1 (IL‐1RAPL1) to inhibit the activation and function of Th1 and Th17 cells, and promotes the proliferation of Treg cells, thus exerting anti‐inflammatory functions. This study also discussed whether BTN3A1 may regulate IL‐38 and then affect inflammation and SLE development. First, we examined IL‐38 expression in the plasma, PBMCs of SLE patients, and healthy volunteers, and the results were consistent with the previous findings [21]. We found lower serum levels of IL‐38 in pristane‐treated BTN3A1^KI^ mice. To test our hypothesis, we injected pristane‐treated BTN3A1^KI^ mice with recombinant IL‐38. Our data revealed amelioration of BTN3A1‐induced lupus‐like changes, such as improved degree of renal injuries and reduced percentage of CD3^+^, CD8^+^ T cells, Th1, Th2, Th17 cells, upregulated percentage of Treg cells, and lower expression of IL‐4, IL‐6, IL‐10, IL‐17A, IFN‐γ, TNF‐α, and dsDNA. However, our study showed that a 50 µg/kg IL‐38 dose was more effective than 100 µg/kg in reducing BTN3A1‐induced inflammation and lupus‐like phenotype, although both reversed the inflammatory responses and renal damages (higher doses were only significantly effective in some indicators). This may be due to high doses of IL‐38‐mediated inactive immunomodulatory effects or changes in pharmacokinetics, which will lead to uneven tissue distribution and decreased target site of IL‐38 concentration [28, 29]. We demonstrated that BTN3A1 inhibited IL‐38 secretion, and then promoted inflammation and induced lupus‐like alterations.

We conducted KEGG pathway enrichment analysis to discuss the mechanisms involved in the BTN3A1‐IL‐38 axis. The ferroptosis signaling pathway was significantly differentially expressed between the pristane‐treated BTN3A1^KI^ mice injected with/without IL‐38, indicating that the ferroptosis signaling may be a key to BTN3A1 inhibition of IL‐38. Indeed, expression of ferroptosis signaling is elevated in the glomeruli of patients with lupus nephritis [30]. Enhanced neutrophil ferroptosis leads to a breakdown of immune tolerance in SLE patients [31]. Moreover, our data revealed that CD4^+^ T cells were significantly differently distributed in the pristane‐treated BTN3A1^KI^ mice injected with/without IL‐38 by single‐cell sequencing. CD4^+^ T cells are involved in the pathogenesis of SLE by auto‐secreting cytokines and inducing B cells to secrete IL‐4, IL‐6, IL‐10 [32]. Therefore, it is hypothesized that CD4^+^ T cells may play an essential role in SLE through inhibition of the IL‐38‐ferroptosis axis by BTN3A1. Ferroptosis signaling‐associated proteins (Steap3, Slc7a11, Slc11a2, Tfrc), glutathione, and malondialdehyde are considered to be key features of ferroptosis [33]. Our results identified that these features were highly expressed in CD4^+^BTN3A1^+/+^ T cells, whereas addition of IL‐38 reduced expression of these proteins, and glutathione and malondialdehyde. Promotion of CD4^+^ T cells activation and imbalanced differentiation of effector T cells was related to aggravation of the lupus‐like features [34]. Iron overload was able to promote CD4^+^ T cell subpopulations proliferation, inflammatory cytokines generation, and autoantibodies generation in lupus mice, and the lupus mice treated with a high‐iron diet showed a higher proportion of CD4^+^ T cell subpopulations [35]. Thus, inhibition of CD4^+^ T cells' function such as proliferation, is essential for the pathogenesis of SLE. By isolating CD4^+^ T cells from BTN3A1^+/+^ mice, the addition of IL‐38 inhibited cell proliferation and apoptosis. IL‐38 can inhibit the conversion of CD4^+^ T cells into Th17, further enhance the immunosuppressive activity of Treg cells, and inhibit the inflammatory response [36]. The occurrence of ferroptosis in different subsets of CD4^+^ T cells is significantly associated with immune imbalance, which indicates that there is a potential relationship between IL‐38 and ferroptosis. These features were further confirmed by the addition of the ferroptosis inhibitor (Ferrostatin‐1), where proliferation and apoptosis of CD4^+^BTN3A1^+/+^ T cells were inhibited by treatment with IL‐38 in the presence of Ferrostatin‐1. To validate our findings, Jurkat T cells were used, and consistent findings were observed in Jurkat T cells. Overall, we confirmed that BTN3A1 inhibition of the IL‐38‐ferroptosis axis affects CD4^+^ T cell biological activity and is involved in lupus pathogenesis. Interestingly, a study showed that BTN3A1 overexpression in APCs inhibited the proliferation and IFN‐γ release from CD4^+^ T cells [26]. BTN3A1 inhibits αβ T cells by binding to N‐mannosylated residues in the human *CD45* gene, thereby eliminating effective TCR activation. However, we endogenously transfected with an adenovirus overexpressing BTN3A1, and this direct effect of overexpression of BTN3A1 for CD4^+^ T cells may have contributed to the accelerated CD4^+^ T cell proliferation. This was confirmed in our in vivo experiments in which BTN3A1 increased the proportion of CD4^+^ T cells (including a higher percentage of Th1, Th2, Th17 cells and a lower percentage of Treg cells) in the lupus‐like mice. Similarly, another study showed that upregulation of BTN3A1 in monocytes leads to activation and accelerated proliferation of Vγ9Vδ2^+^ T cells in patients with psoriasis vulgaris [16]. Thus, our findings strongly indicated that BTN3A1 is functionally indispensable in promoting CD4^+^ T cells proliferation and apoptosis in the lupus‐like mice.

Nevertheless, this study has several limitations. First, although it has been demonstrated that BTN3A1 can regulate the expression of IL‐38, the precise molecular mechanisms underlying this regulation remain to be fully elucidated. Future research should delve deeper into the signaling pathways and molecular interactions involved to clarify how BTN3A1 modulates IL‐38 expression. Second, the systemic manifestations of BTN3A1^KI^ mice require further investigation. Organs commonly affected in lupus, such as lymph nodes and joints, were not comprehensively evaluated in this study. In future studies, a more detailed assessment of these organs needs to be conducted to provide a more complete understanding of the disease phenotypes in BTN3A1^KI^ mice.

In summary, this study found that BTN3A1 expression was increased in SLE patients, and BTN3A1 inhibited the IL‐38‐ferroptosis axis, and then, promoted inflammation and induced lupus‐like disease. Our study also provided insights into the involvement of CD4^+^ T cells in the mechanisms of SLE disease pathogenesis and highlighted the potential role of BTN3A1 for SLE therapy. It is expected that in the future we will purify BTN3A1 for use in clinical trials for the treatment of SLE patients.

## 4 Materials and Methods

### 4.1 Patients

A total of 328 SLE patients from the Affiliated Hospital of Southwest Medical University were included to detect the plasma (*N* = 280), mRNA (*N* = 30) expression of BTN3A1, and BTN3A1 expression in different immune cells (*N* = 18). One hundred and forty‐eight healthy controls from the Affiliated Hospital of Traditional Chinese Medicine of Southwest Medical University were used as controls. Plasma (*N* = 100), mRNA (*N* = 30) expression of BTN3A1, and expression of BTN3A1 in different immune cells (*N* = 18) were detected in healthy controls. Detailed information on patients and controls is shown in Table S11 and Table S12. The diagnosis of SLE meets the 1997 American College of Rheumatology criteria. SLE patients with pregnancy, infections, tumors, and other rheumatic diseases were excluded. Blood samples were collected, and each participant signed an informed consent form. The study was approved by the Ethics Committee of the Affiliated Hospital of Southwest Medical University (KY2020180) and the Affiliated Hospital of Traditional Chinese Medicine of Southwest Medical University (BY2022005).

### 4.2 Animal Model

We engineered a knock‐in mouse expressing the human *BTN3A1* gene under the control of the mouse *Itgax/Cd11c* promoter (BTN3A1^KI^). In brief, we created a β‐globin‐BTN3A1‐β‐globin knock‐in at the locus of ROSA26 in C57BL/6 mice by CRISPR/Cas‐mediated genome engineering (Figure S3A,B). WT female C57BL/6 (7 weeks) mice were purchased from GemPharmatech (Chengdu, China). Experiments were conducted in two stages, and a lupus‐like disease was induced by intraperitoneal injection of 500ul pristane (Sigma Aldrich, St Louis, USA) for three months. In the first stage, BTN3A1^KI^ and WT mice were categorized into four groups (WT‐PBS group, WT‐Pristane group, BTN3A1^KI^‐PBS group, BTN3A1^KI^‐Pristane group). Mice in the WT group and BTN3A1^KI^ group were intraperitoneally injected with 500ul PBS at Week 8, and mice in the WT‐Pristane group and BTN3A1^KI^‐Pristane group were intraperitoneally injected with pristane at Week 8. Samples of venous blood from the inner canthus, liver, spleen, and kidneys were collected for subsequent analysis after 3 months’ observation. In the second stage, BTN3A1^KI^ mice were divided into four groups (BTN3A1^KI^‐Pristane‐PBS group, BTN3A1^KI^‐Pristane‐25ug/kg IL‐38 group, BTN3A1^KI^‐Pristane‐50ug/kg IL‐38 group, and BTN3A1^KI^‐Pristane‐100ug/kg IL‐38 group). Mice in the BTN3A1^KI^‐Pristane groups were injected with different doses of recombinant mouse IL‐38 (25, 50, and 100ug/kg, Sino Biological, Beijing, China) or PBS for a duration of two weeks (with intraperitoneal injection every other day). The mice were then monitored for one week and used for subsequent testing. A flowchart of the animal experiments was shown in Figure S3C. All animal experiments were approved by the Ethics Committee of Southwest Medical University (swmu20230093).

### 4.3 Enzyme‐Linked Immunosorbent Assay (ELISA)

BTN3A1 and IL‐38 levels in the plasma of SLE patients and healthy controls were detected by ELISA kits (Cusabio, Wuhan, China). Serum samples from mice were collected from venous blood of the inner canthus, and the levels of IL‐38 and dsDNA were detected by ELISA kits (Cusabio, Wuhan, China).

### 4.4 Flow Cytometry

Peripheral blood mononuclear cells (PBMCs) from SLE patients and healthy controls were obtained by lymphocyte separation medium with density gradient centrifugation (TBDscience, Tianjing, China). CD3^+^ (CD3‐APC‐Cy7), CD4^+^ (CD4‐BB515), CD8^+^ (CD8‐APC) T cells, CD19^+^ (CD19‐APC), CD14^+^ (CD14‐BV421), CD11b^+^ (CD11b‐FITC), CD11c^+^ (CD11c‐BV421) cells in PBMCs were recognized by flow cytometry. In addition, expression of BTN3A1 (BTN3A1‐PE) was detected in all of the immune cells as described above.

Mouse spleen was ground on a 70‐micrometer cell strainer (Novbio, China) and then lysed with erythrocyte lysate (Beyotime, Shanghai, China) on ice. CD3^+^ (CD3‐FITC), CD4^+^ (CD4‐FITC), CD8^+^ (CD8‐APC) T cells, CD19^+^ (CD19‐APC), CD14^+^ (CD14‐FITC), CD11b^+^ (CD11b‐APC), CD11c^+^ (CD11c‐FITC) cells were detected directly by flow cytometry. After labeling CD4^+^ T cells, the cells were fixed (RD system, Minnesota, USA) and membrane‐broken (RD system, Minnesota, USA), and finally, fluorescently labeled with Th1 (IFN‐γ‐PE‐CF594), Th2 (IL‐4‐APC), Th17 (IL‐17A‐APC‐CyTM7), and Treg (Foxp3‐PE) cells.

All flow cytometry antibodies were purchased from BD Biosciences (California, USA), and data were assayed under a BD FACSCalibur flow cytometer (Becton, USA).

### 4.5 Histopathology

Kidneys of mice were fixed using 4% formaldehyde and then embedded in paraffin, cut with a thickness of 5 um sections. The kidneys were stained with hematoxylin‐eosin staining (HE) and Ponceau, Fuchsin, and Aniline blue staining (Masson), respectively. HE score (Table S13) and Masson assay were performed to assess the extent of renal injury and renal fibrosis, respectively. Immune complexes deposited in the kidneys were evaluated by FITC‐coupled anti‐mouse IgG with immunofluorescence. Moreover, kidneys were pre‐fixed with 3% glutaraldehyde, re‐fixed with 1% osmium tetroxide, dehydrated and embedded, and sectioned. Ultrastructural alterations of renal tubular epithelial cells and glomeruli were visualized under TEM after staining with uranyl acetate and lead citrate.

### 4.6 Cytometric Bead Array System (CBA)

Serum concentrations of cytokines IL‐4, IL‐6, IL‐10, IL‐17A, IFN‐γ, and TNF‐α in different groups of mice were measured by the CBA Mouse Th1/Th2/Th17 Cytokine Kit (BD Biosciences, San Diego, CA) on a cellular Counting Bead Array (CBA).

### 4.7 Single‐Cell RNA Sequencing

The library was established after the mice spleen tissue was taken and a single cell suspension was prepared. During library construction, the number of genes identified by the cells was filtered out to be less than 200 or greater than the maximum number of genes ×90%. The proportion of mitochondrial reads of the cells was filtered out to be sorted in the top 15%, and to correct for the effect of the cell cycle. Final sequencing was performed using Combinatorial Probe‐Anchor Synthesis (cPAS).

### 4.8 Transcriptomics and Proteomics

For transcriptomics, spleen tissue from mice was taken for RNA purification and collection, and the products were amplified and sequenced. The raw data obtained from sequencing were filtered to remove reads containing junctions (junction contamination); reads with unknown base N content greater than 5%; and low‐quality reads (reads in which the proportion of bases with a mass value of less than 15 to the total number of bases in the read is greater than 20% are considered to be low‐quality reads), and then the clean data were obtained.

Regarding proteomics, spleen tissue was milled on the ice and tissue was lysed using RIPA lysis solution. Lysis was further assisted by the treatment of ultrasound. Samples of protein solution were obtained by centrifugation. Then, the samples were proteolytically digested and evaluated by DIA mass spectrometry.

### 4.9 CD4^+^ T Cells Isolation and Culture

Wild‐type C57BL/6 mice were subjected to human *BTN3A1* gene overexpression (BTN3A1^+/+^) adenovirus injection (Genechem, Shanghai, China). Five days later, naïve CD4^+^ T cells were obtained from spleens by magnetic bead sorting (Miltenyi Biotec, Germany). Naïve CD4^+^ T cells (10^6^/500uL) were cultured in 10% fetal bovine serum (FBS) (Gibco, Massachusetts, USA), 1% penicillin‐streptomycin antibody (Beyotime, Chengdu, China) and activated with anti‐CD3 (2ug/ml) (Invitrogen, Massachusetts, USA), anti‐CD28 (2ug/mL) (Invitrogen, Massachusetts, USA), IL‐2 (2 ng/mL) (Peprotech, New Jersey, USA) for 24 h. Then, the cells were stimulated with recombinant mouse IL‐38 stimulation (24 h), and used for the following studies.

In addition, the Jurkat T cells were cultured in 10% FBS, 1% penicillin‐streptomycin antibody. After BTN3A1 overexpressed adenovirus transfection (5 days) and then recombinant human IL‐38 stimulation (24 h), the cells were collected for the following studies.

### 4.10 Detection of Fe^2+^, Glutathione, Malondialdehyde Levels

CD4^+^BTN3A1^+/+^ T cells and BTN3A1^+/+^ Jurkat T cells were collected and lysed by cell sonication. Then, the cells were assayed for levels of Fe^2+^ (Abcam, Cambridge, UK), glutathione (Beyotime, Chengdu, China), and malondialdehyde (Beyotime, Chengdu, China), respectively.

### 4.11 Cell Proliferation and Apoptosis

Cell proliferation of CD4^+^BTN3A1^+/+^ T cells and BTN3A1^+/+^ Jurkat T cells was determined by CCK8 kits (Abmole, Houston, USA) in the presence or absence of inhibitors of ferroptosis (Ferrostatin‐1, 2uM) (Monmouth Junction, NJ, USA) for 24 h.

Similarly, CD4^+^BTN3A1^+/+^ T cells and BTN3A1^+/+^ Jurkat T cells were treated with/without Ferrostatin‐1 for 24 h, and cell apoptosis was detected using FITC Annexin V Apoptosis Detection Kit I (BD Biosciences, California, USA) and APC Annexin V Apoptosis Detection Kit (Vazyme, Nanjing, China).

### 4.12 Reverse Transcription‐Quantitative PCR

RNA was extracted from the PBMCs of SLE patients and healthy controls, CD4^+^ T cells, and Jurkat T cells using the EZ‐press RNA Purification Kit (HiFoBio, Shanghai, China). cDNA was constructed by PrimeScript RT Master Mix (Vazyme, Nanjing, China). qPCR was performed under the following thermal cycling conditions: 95°C for 30s, followed by 40 cycles of 95°C for 10s and 60°C for 30s. GAPDH was used as an internal reference. Primer sequences are shown in Table S14.

### 4.13 Statistical Analysis

Normally distributed quantitative data are presented as mean±standard deviation, and were compared by Student's *t*‐test or one‐way analysis of variance. The Mann‐Whitney U test was used for quantitative data that were not normally distributed. Correlation analysis was used for both metrics. All analyses were performed using GraphPad Prism 5.0.

## Author Contributions

Study conception and design: WDX, DCW, YYT, QH, LCS, AFH. Acquisition of data, analysis and interpretation of data: WDX, DCW, YYT, QH, SYF, LF, YYC, LQY, LCS, AFH. Drafting the article: WDX, DCW, YYT, QH, LCS, AFH. Final approval of the version of the article to be published: all authors, and that all authors agree to be accountable for all aspects of the work.

## Ethics Statement

All animal experiments were approved by the Ethics Committee of Southwest Medical University (swmu20230093). The study was approved by the Ethics Committee of the Affiliated Hospital of Southwest Medical University (KY2020180) and the Affiliated Hospital of Traditional Chinese Medicine of Southwest Medical University (BY2022005).

## Conflicts of Interest

All authors declare no conflicts of interest.

## Supporting information



Supporting Information

## Data Availability

Datasets are available from the corresponding author on reasonable request. The mass spectrometry proteomics data have been deposited to the ProteomeXchange Consortium via the PRIDE partner repository with the dataset identifier PXD064490, and we have uploaded the raw data of RNAseq, sc‐RNAseq to NCBI‐SRA with project PRJNA1267837 and PRJNA1269121, respectively.
