# Supplementary material for: Butyrophilin 3A1 Contributes to Inflammation and Induces a Lupus‐Like Disease by Inhibiting the IL‐38‐Ferroptosis Axis
Source: MedComm (2020). 2025 Sep 14;6(9):e70356. doi: 10.1002/mco2.70356 (PMC12433891; doi:10.1002/mco2.70356)
Supplement: Supplementary file 1 — Supporting Information [file MCO2-6-e70356-s001.docx]

**Butyrophilin 3A1 contributes to inflammation and induces a lupus-like disease by inhibiting the IL-38-ferroptosis axis**

Wang-Dong Xu^1#^, Da-Cheng Wang^1#^, Yang-Yang Tang^1#^, Qi Huang^2#^, Lu Fu^3^, You-Yue Chen^1^, Lu-Qi Yang^1^, Si-Yu Feng^1^, Lin-Chong Su^4*^, An-Fang Huang^5**^

^1^Department of Evidence-Based Medicine, School of Public health, Southwest Medical University, 1 Xianglin Road, Luzhou, 646000, Sichuan, China.

^2^Vanke School of Public Health, Tsinghua University, 30 Shuangqing Road, Haidian District, 100084, Beijing, China.

^3^Laboratory Animal Center, Southwest Medical University, 1 Xianglin Road, Luzhou, 646000, Sichuan, China.

^4^Hubei Provincial Key Laboratory of Occurrence and Intervention of Rheumatic diseases, Affiliated Minda Hospital of Hubei Minzu University, 2 Wufengshan Road, Enshi, 445000, Hubei, China; Department of Rheumatology and Immunology, Affiliated Minda Hospital of Hubei Minzu University, 2 Wufengshan Road, Enshi, 445000, Hubei, China.

^5^Department of Rheumatology and Immunology, the Affiliated Hospital, Southwest Medical University, 25 Taiping Road, Luzhou, 646000, Sichuan, China.

^#^Wang-Dong Xu, Da-Cheng Wang, Yang-Yang Tang, and Qi Huang contributed equally.

^*^Corresponding author: Lin-Chong Su, E-mail: linchong2019@163.com

^**^Corresponding author: An-Fang Huang, E-mail address: loutch211@163.com

**Supplementary Figures**


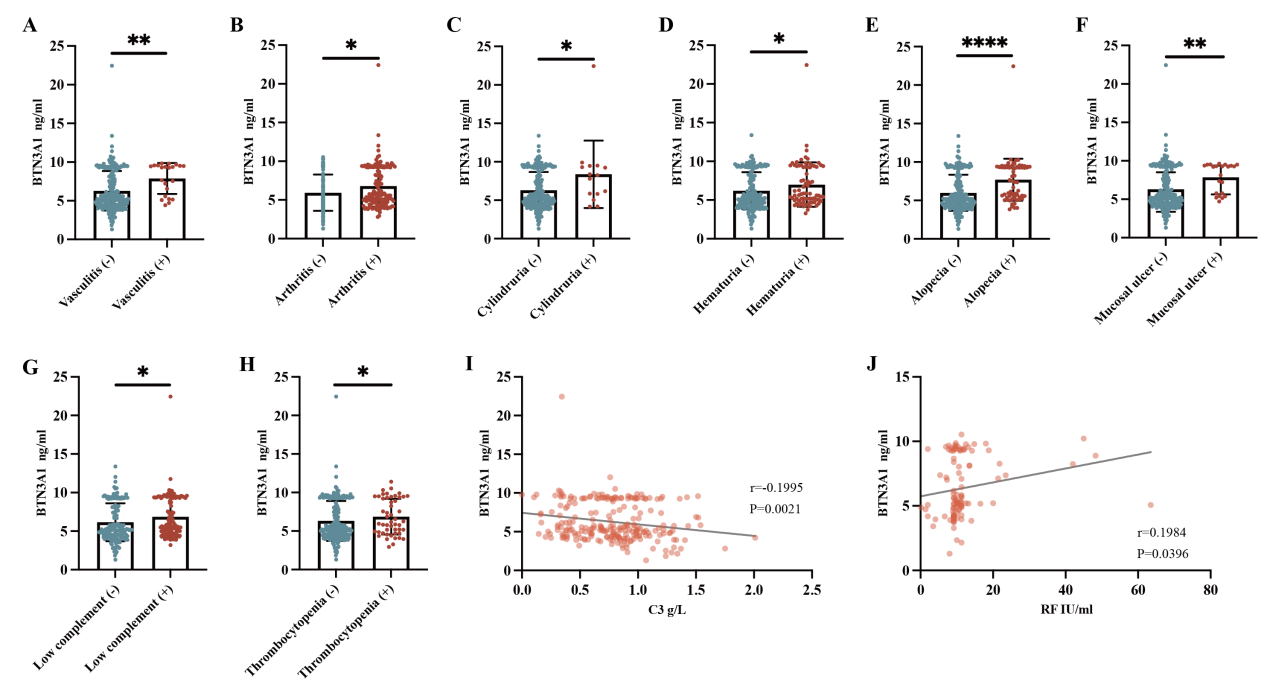


**Figure S1 Plasma levels of BTN3A1 correlated with different clinical, laboratory characteristics in SLE patients.** Comparison of plasma levels of BTN3A1 in SLE patients was discussed in different subgroups. (A) Vasculitis (+) (N=22); (B) Arthritis (+) (N=153); (C) Cylindruria (+) (N=15); (D) Hematuria (+) (N=77); (E) Alopecia (+) (N=69); (F) Mucosal ulcer (+) (N=22); (G) Low complement (+) (N=110); (H) Thrombocytopenia (+) (N=50). (I) Correlation analysis of C3 expression and BTN3A1 plasma levels in SLE patients. (J) Correlation analysis of rheumatoid factor (RF) expression and BTN3A1 plasma levels in SLE patients. *P<0.05, **P<0.01, and ****P<0.0001.


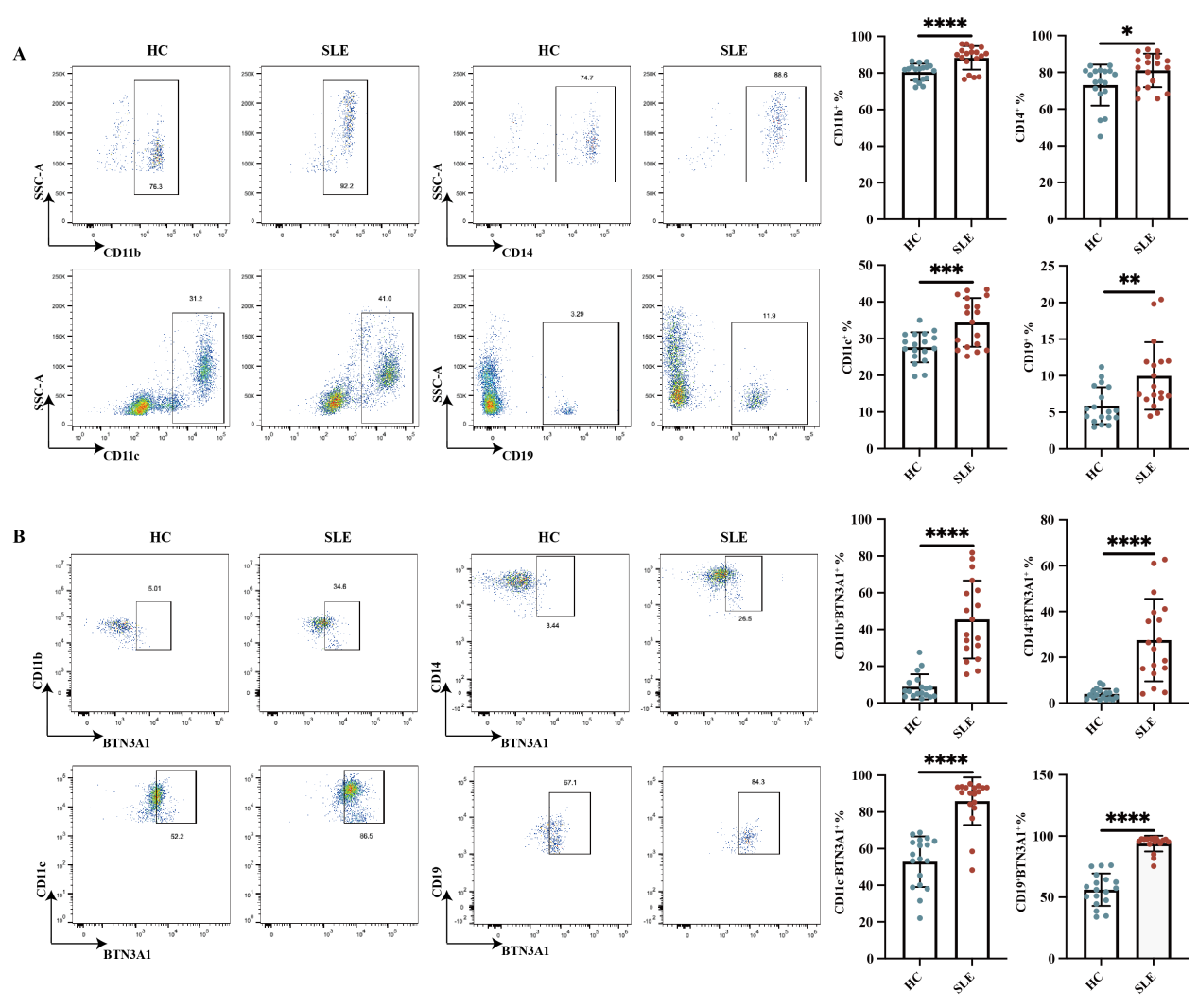


**Figure S2 Different proportion of immune cells and BTN3A1 expression in immune cells between SLE patients and healthy controls.** (A) Representative flowchart and statistical analysis of CD14^+^, CD11b^+^, CD19^+^, CD11c^+^ cells in peripheral blood of healthy controls (N=18) and SLE patients (N=18). (B) Representative flowchart and statistical analysis of BTN3A1 expression in CD14^+^, CD11b^+^, CD19^+^, CD11c^+^ cells in peripheral blood of healthy controls (N=18) and SLE patients (N=18). *P<0.05, **P<0.01, ***P<0.001 and ****P<0.0001.


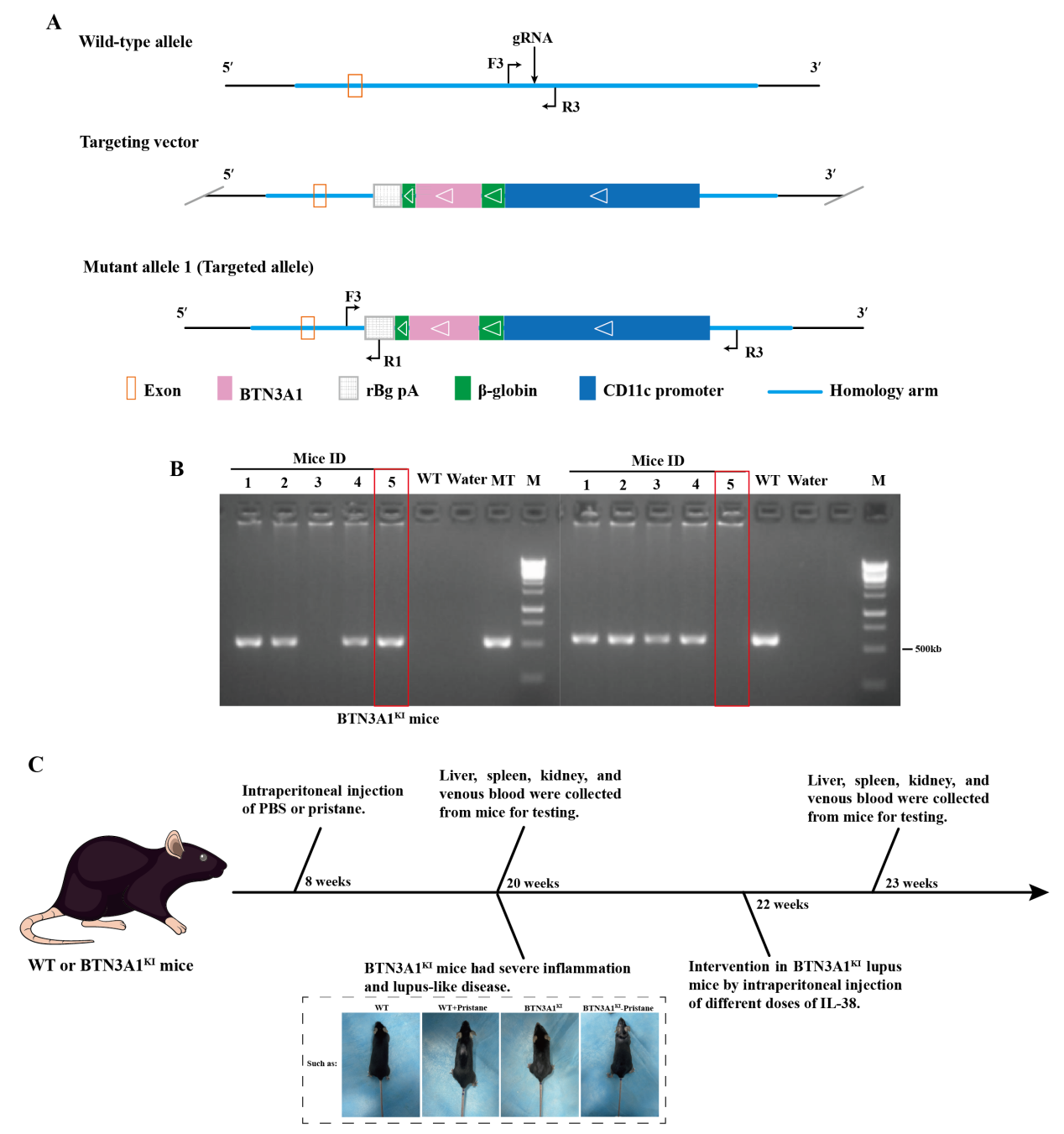


**Figure S3 Construction of BTN3A1^KI^ mouse model and flow chart of animal experiments.** (A) Overview of the targeting strategy for BTN3A1^KI^ mouse model. Creating a β-globin-BTN3A1-β-globin knock-in at the locus of ROSA26 in C57BL/6 mice by CRISPR/Cas-mediated genome engineering. (B) Establishment of BTN3A1^KI^ mouse model was verified by qRT-PCR. Primer 1 (F1: 5’-AAGCACGTTTCCGACTTGAGTTG-3’; R1: 5’-CCATAGAAAAGCCTTGACTTGAGGTT-3’) and Primer 2 (F1: 5’-AAGCACGTTTCCGACTTGAGTTG-3’; R1: 5’-GGGTGAGCATGTCTTTAATCTACC-3’) were used to detect different genotypes, with one 505 bp band for homozygotes, one 505 bp band and one 607 bp band for heterozygotes, and one 607 bp band for wild-type allele. (C) Animal experiments schedule.


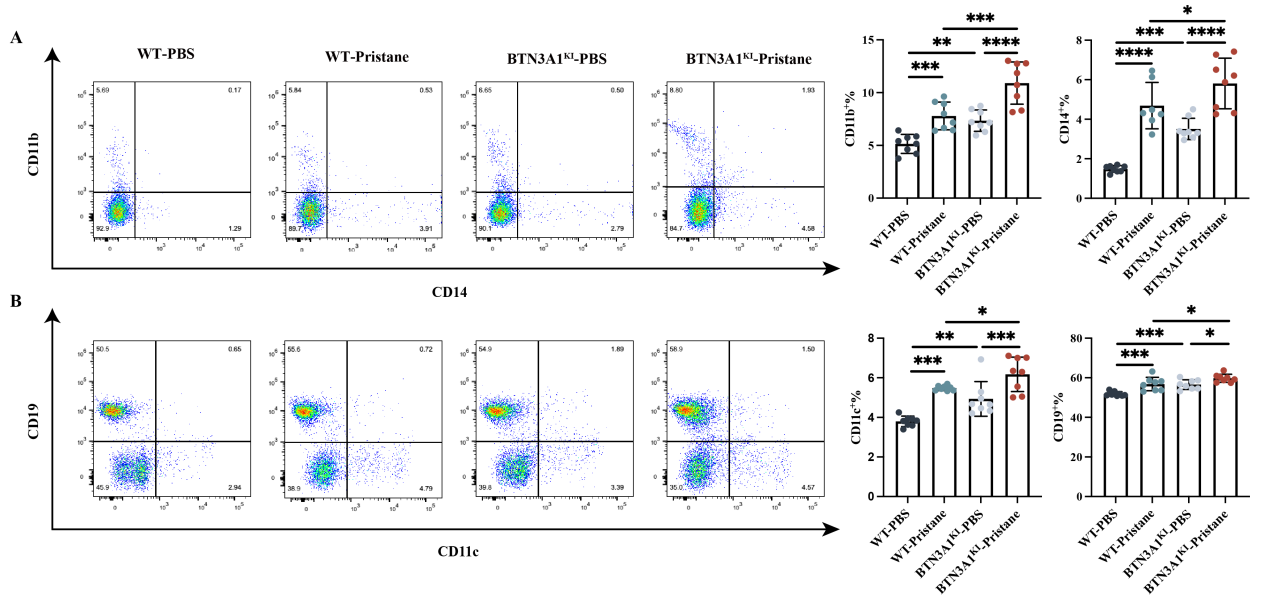


**Figure S4 BTN3A1 promotes abnormalities in immune cells.** (A-B) Representative flowchart and statistical analysis of CD14^+^, CD11b^+^, CD19^+^, CD11c^+^ cells from mice in the wild-type (WT)-PBS (N=8), WT-Pristane (N=8), BTN3A1^KI^-PBS (N=8), and BTN3A1^KI^-Pristane groups (N=8). *P<0.05, **P<0.01, ***P<0.001 and ****P<0.0001.


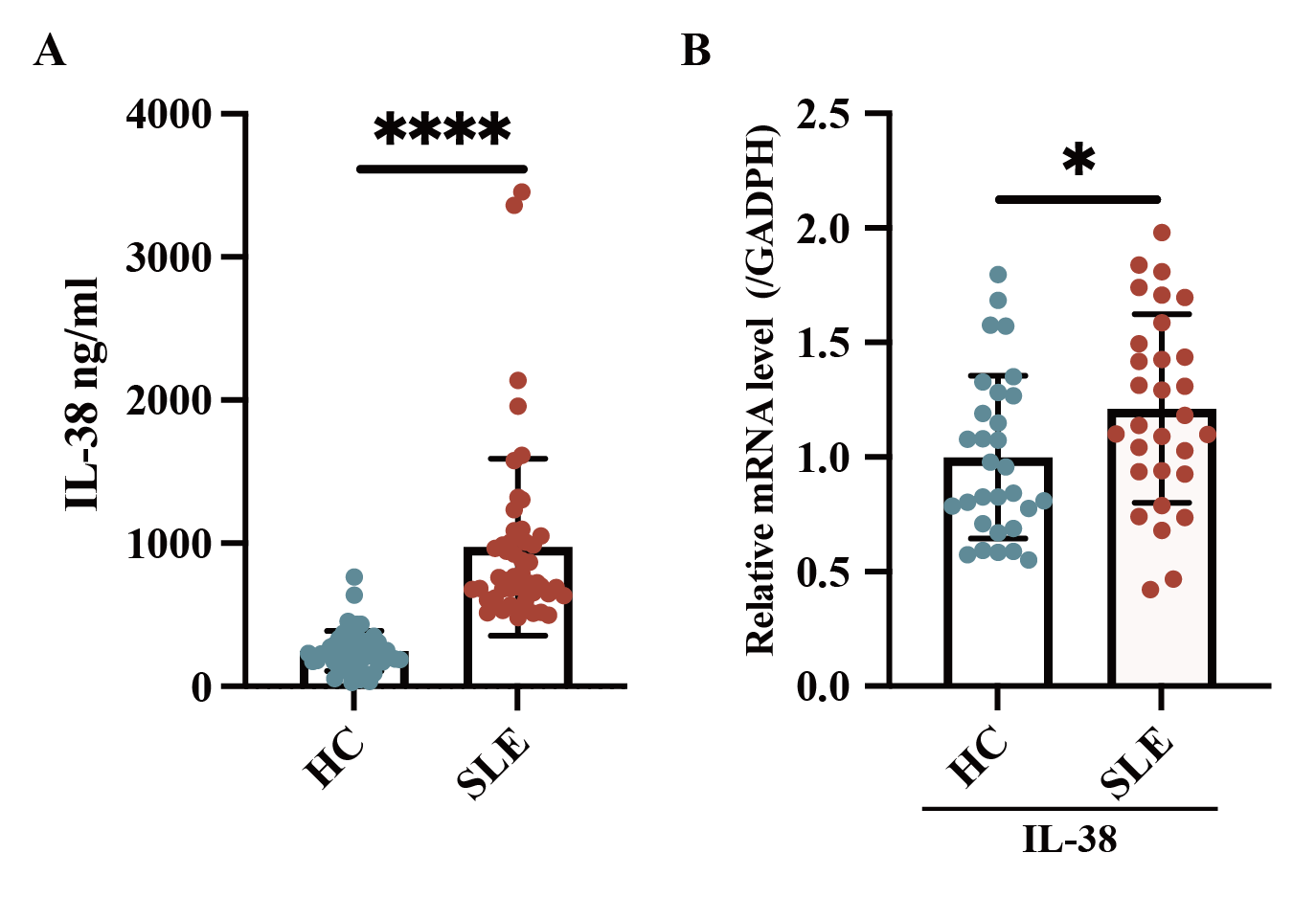


**Figure S5 Plasma levels of IL-38 and mRNA expression of IL-38 in SLE patients.** (A) Plasma levels of IL-38 in healthy controls (N=50) and SLE patients (N=80) were detected by ELISA. (B) Detection of mRNA expression of IL-38 in healthy controls (N=30) and SLE (N=30) patients by qRT-PCR. *P<0.001, and ****P<0.0001.


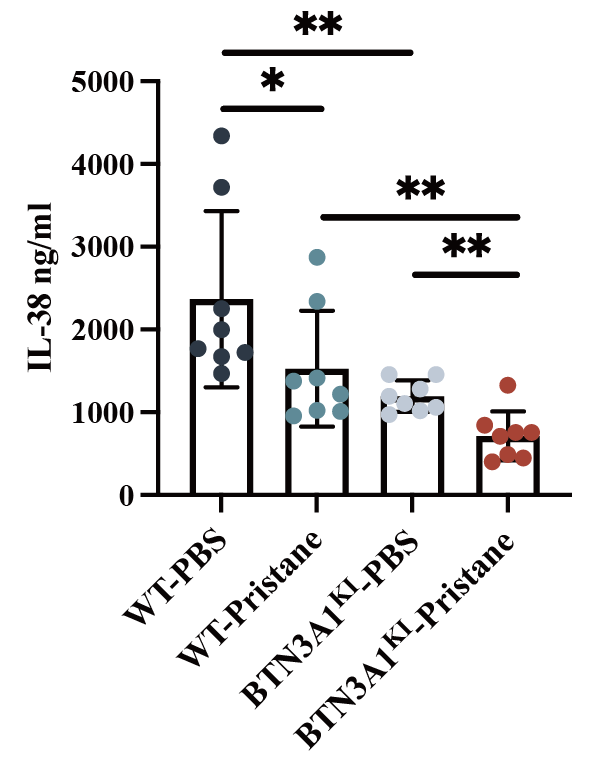


**Figure S6 BTN3A1 inhibits IL-38 expression.** Serum levels of IL-38 were detected by ELISA in mice from the wild-type (WT)-PBS (N=8), WT-Pristane (N=8), BTN3A1^KI^-PBS (N=8), and BTN3A1^KI^-Pristane groups (N=8). *P<0.05, and **P<0.01.


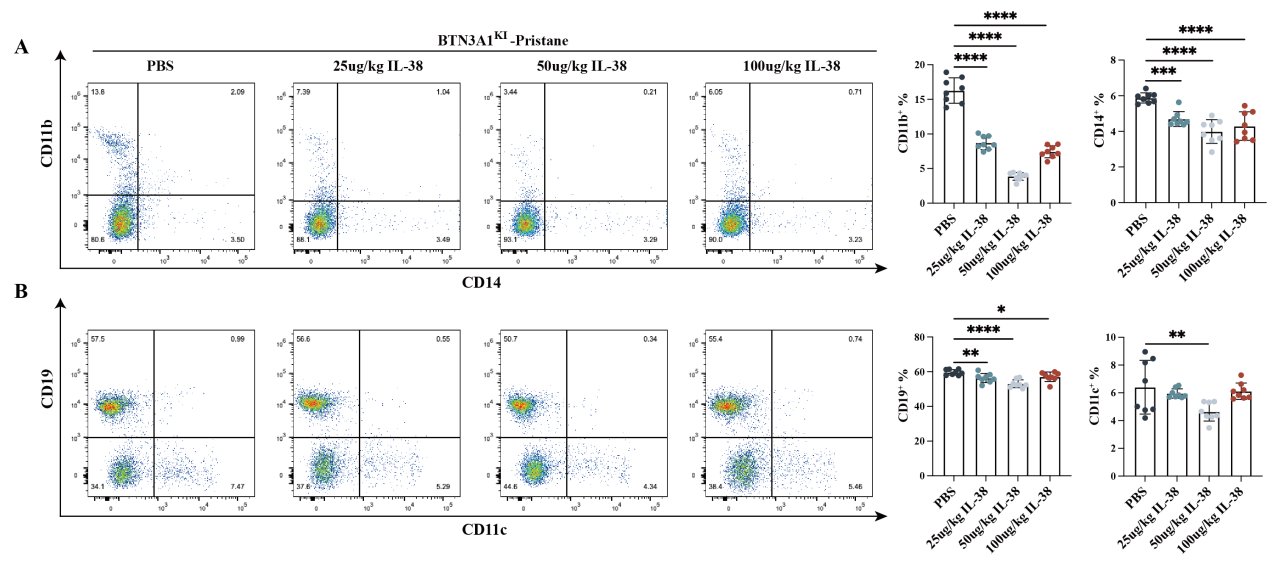


**Figure S7 IL-38 attenuates the highly expressed immune cells in mice from the BTN3A1^KI^-Pristane group.** (A-B) Representative flowchart and statistical analysis of CD14^+^, CD11b^+^, CD19^+^, CD11c^+^ cells from mice in the BTN3A1^KI^-Pristane groups (with/without injection of different concentrations of IL-38, N=8/group). *P<0.05, **P<0.01, ***P<0.001 and ****P<0.0001.


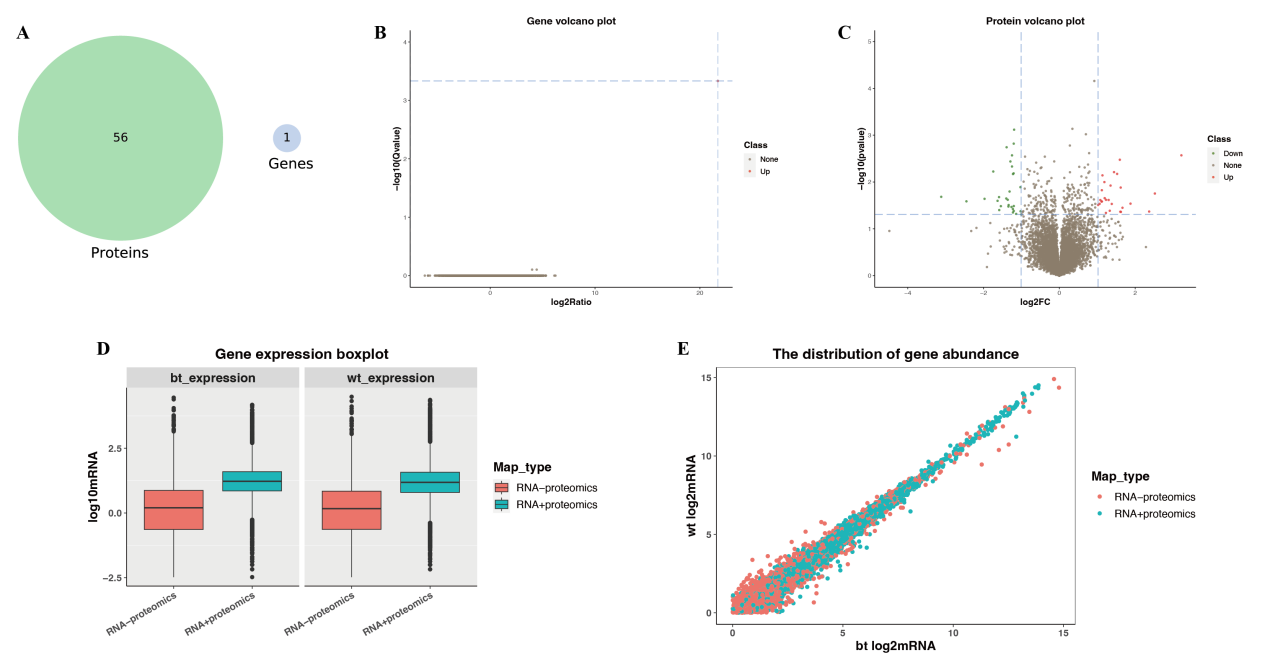


**Figure S8 Cross-analysis of transcriptomics and proteomics data when comparing mice in the WT-PBS group to BTN3A1^KI^-PBS group.** (A) Venn diagram visualized the intersection of differentially expressed proteins and differentially expressed genes (N=3/group). (B-C) Volcano diagram visualized differentially expressed genes and differentially expressed proteins for transcriptomics and proteomics, respectively (N=3/group). (D-E) Box plots and scatter plots visualized the abundance of genes associated with proteins in mice from the wild-type (WT)-PBS group (N=3) and BTN3A1^KI^-PBS group (N=3). Red represents the distribution of gene abundance in transcriptomics associated to proteomics. Green represents the distribution of gene abundance in transcriptomics not associated to proteomics.


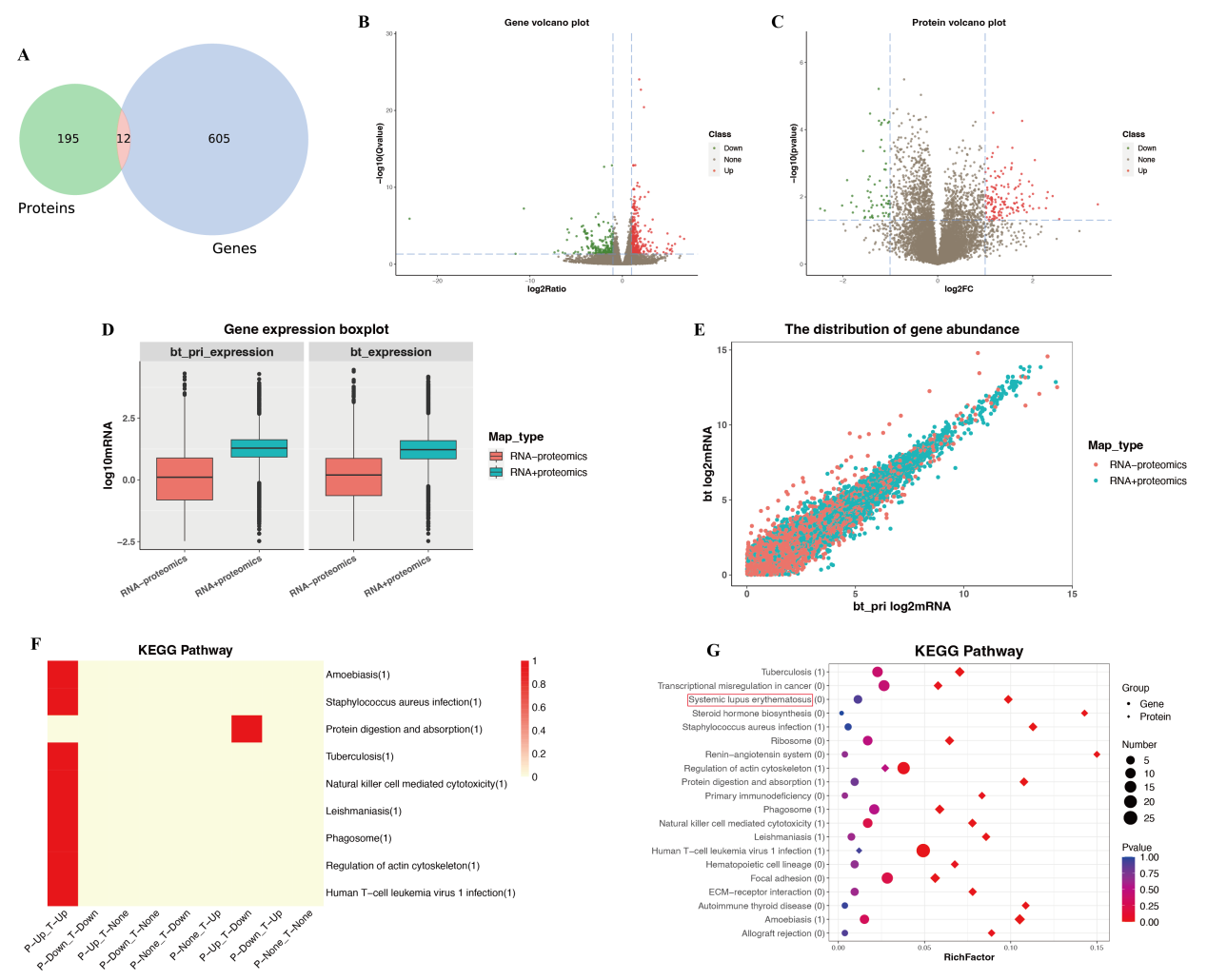


**Figure S9** **Cross-analysis of transcriptomics and proteomics data when comparing mice in the BTN3A1^KI^-PBS group to BTN3A1^KI^-Pristane group.** (A) Venn diagram visualized the intersection of differentially expressed proteins and differentially expressed genes (N=3/group). (B-C) Volcano diagram visualized the differentially expressed genes and differentially expressed proteins for transcriptomics and proteomics, respectively (N=3/group). (D-E) Box plots and scatter plots visualized the abundance of genes associated with proteins in mice from the BTN3A1^KI^-PBS group (N=3) and BTN3A1^KI^-Prisane group (N=3). Red represents the distribution of gene abundance in transcriptomics associated to proteomics. Green represents the distribution of gene abundance in transcriptomics not associated to proteomics. (F-G) KEGG pathway enrichment showed key pathways, and KEGG enrichment heatmap showed distribution of key genes and key proteins.


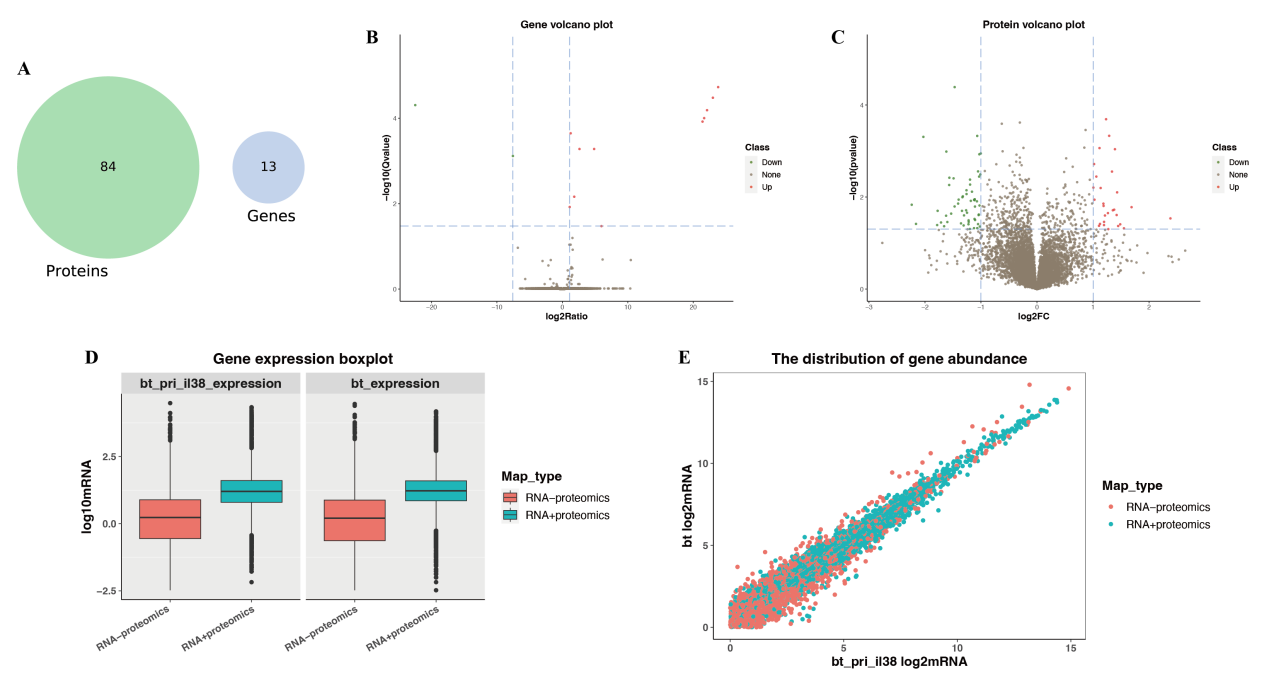


**Figure S10** **Cross-analysis of transcriptomics and proteomics data when comparing mice in the BTN3A1^KI^-PBS group to BTN3A1^KI^-Pristane-IL-38 group.** (A) Venn diagram visualized the intersection of differentially expressed proteins and differentially expressed genes (N=3/group). (B-C) Volcano diagram visualized the differentially expressed genes and differentially expressed proteins for transcriptomics and proteomics, respectively (N=3/group). (D-E) Box plots and scatter plots visualized the abundance of genes associated with proteins in mice from the BTN3A1^KI^-PBS group (N=3) to BTN3A1^KI^-Pristane-IL-38 group (N=3). Red represents the distribution of gene abundance in transcriptomics associated to proteomics. Green represents the distribution of gene abundance in transcriptomics not associated to proteomics.


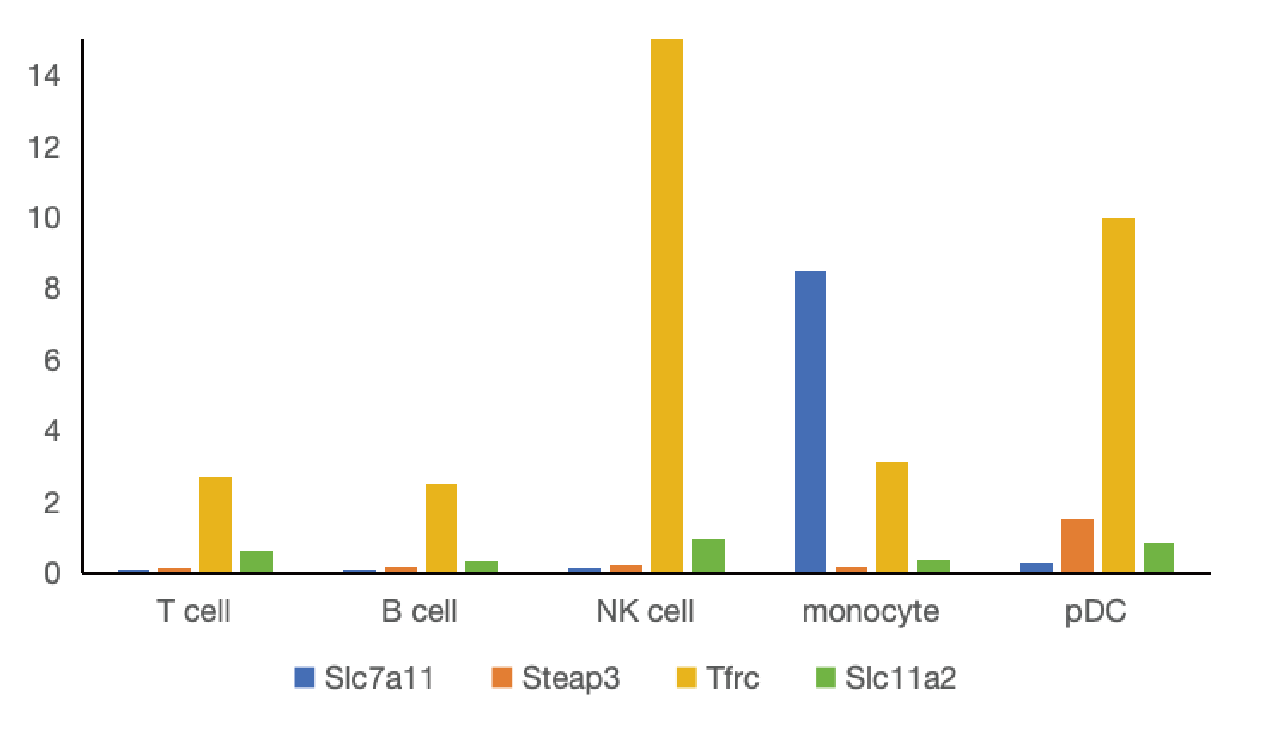


**Figure S11 Levels of ferroptosis-related proteins in five major immune cells.** Single-cell sequencing of T cells, B cells, monocytes, NK cells, pDC cells showed differently expressed gene expression of the ferroptosis-related proteins (Slc11a2, Slc7a11, Steap3, Tfrc) in BTN3A1^KI^-Pristane-PBS (N=3) vs BTN3A1^KI^-Pristane-IL-38 group (N=3).


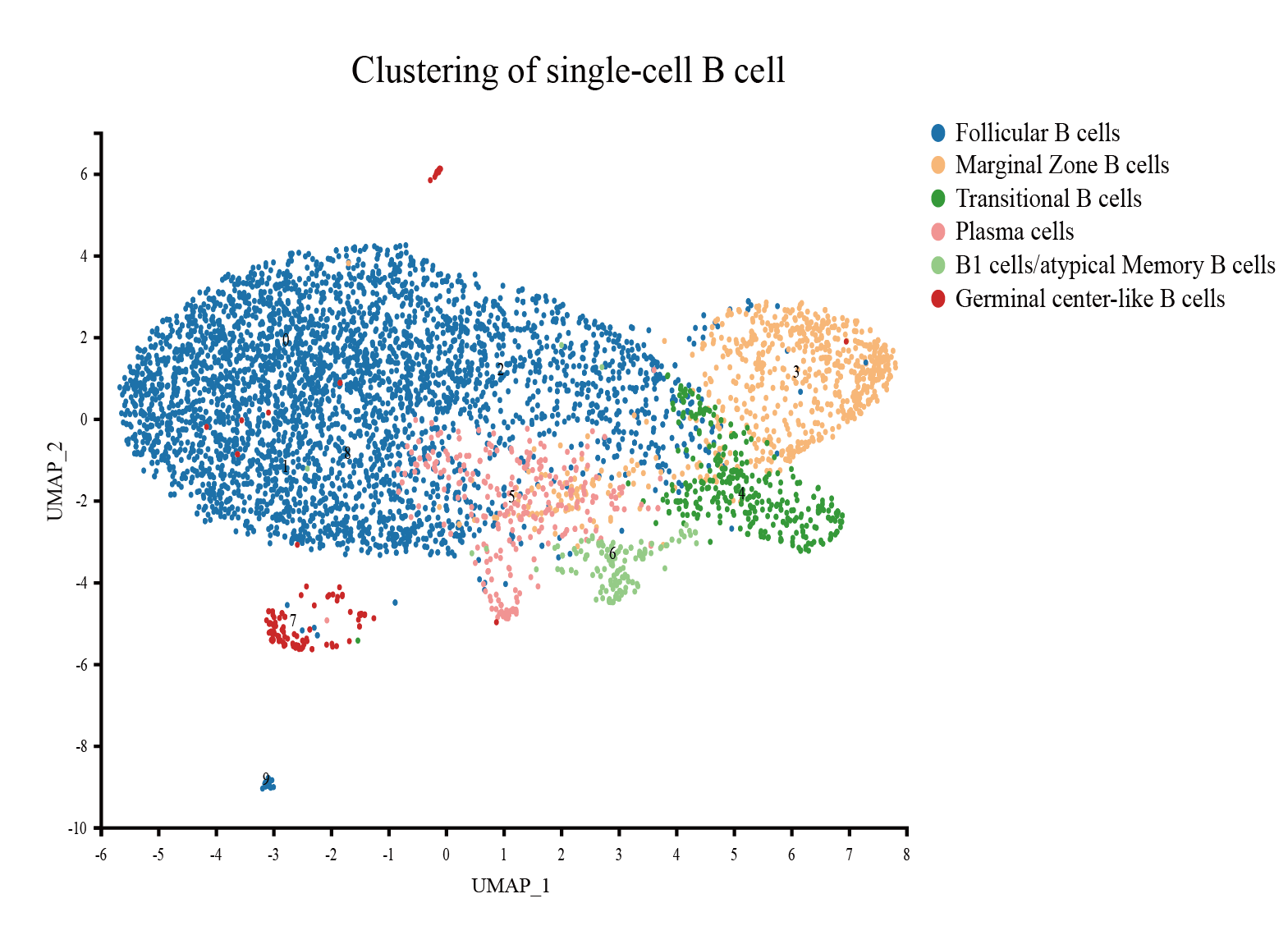


**Figure S12 Re-clustering of B cells in single-cell sequencing.** UMAP plot showed B cell subpopulations distribution in BTN3A1^KI^-Pristane-PBS (N=3) vs BTN3A1^KI^-Pristane-IL-38 group (50ug/kg IL-38, N=3).


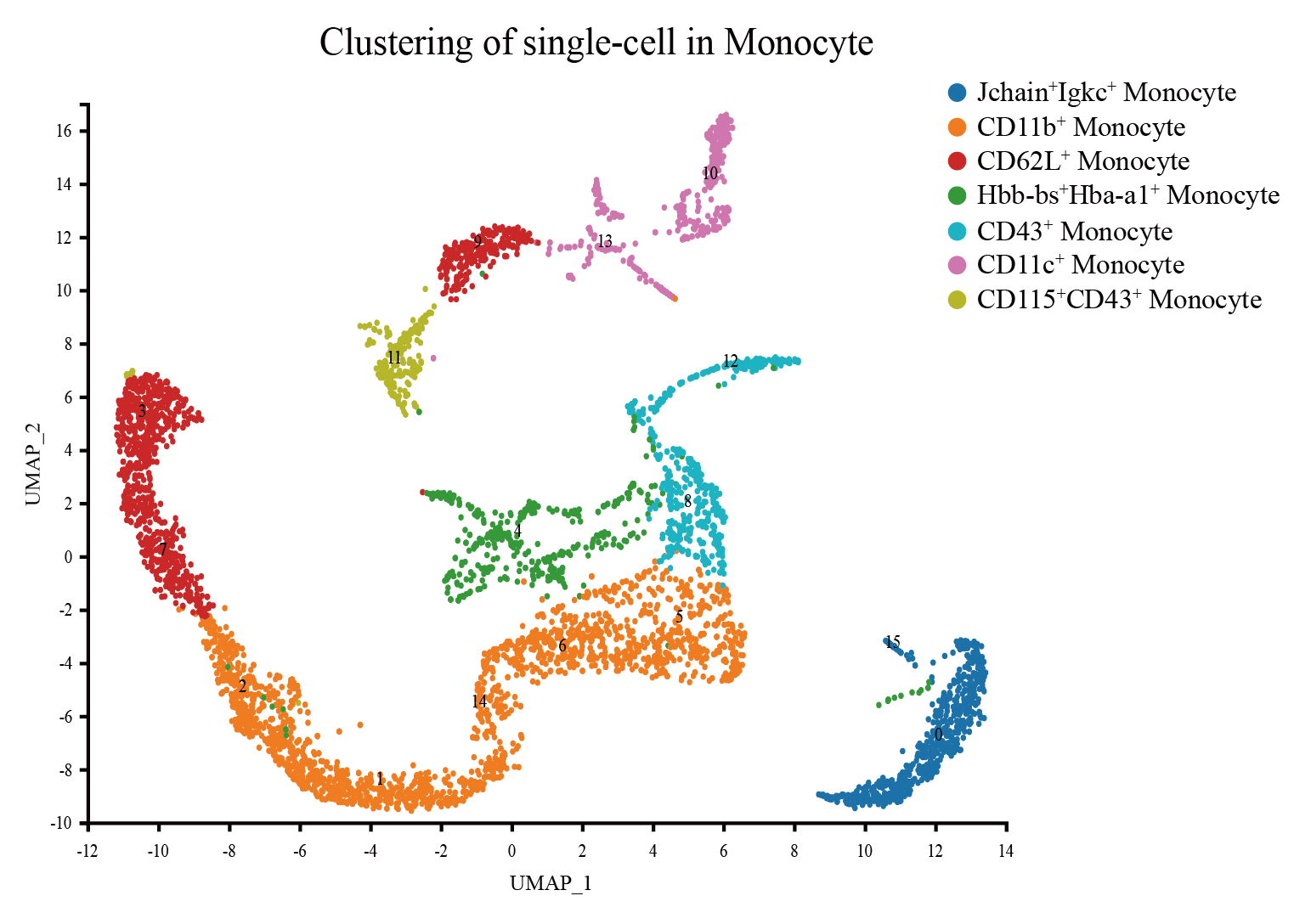


**Figure S13 Re-clustering of monocytes in single-cell sequencing.** UMAP plot showed monocyte subpopulations distribution in BTN3A1^KI^-Pristane-PBS (N=3) vs BTN3A1^KI^-Pristane- IL-38 group (50ug/kg IL-38, N=3).


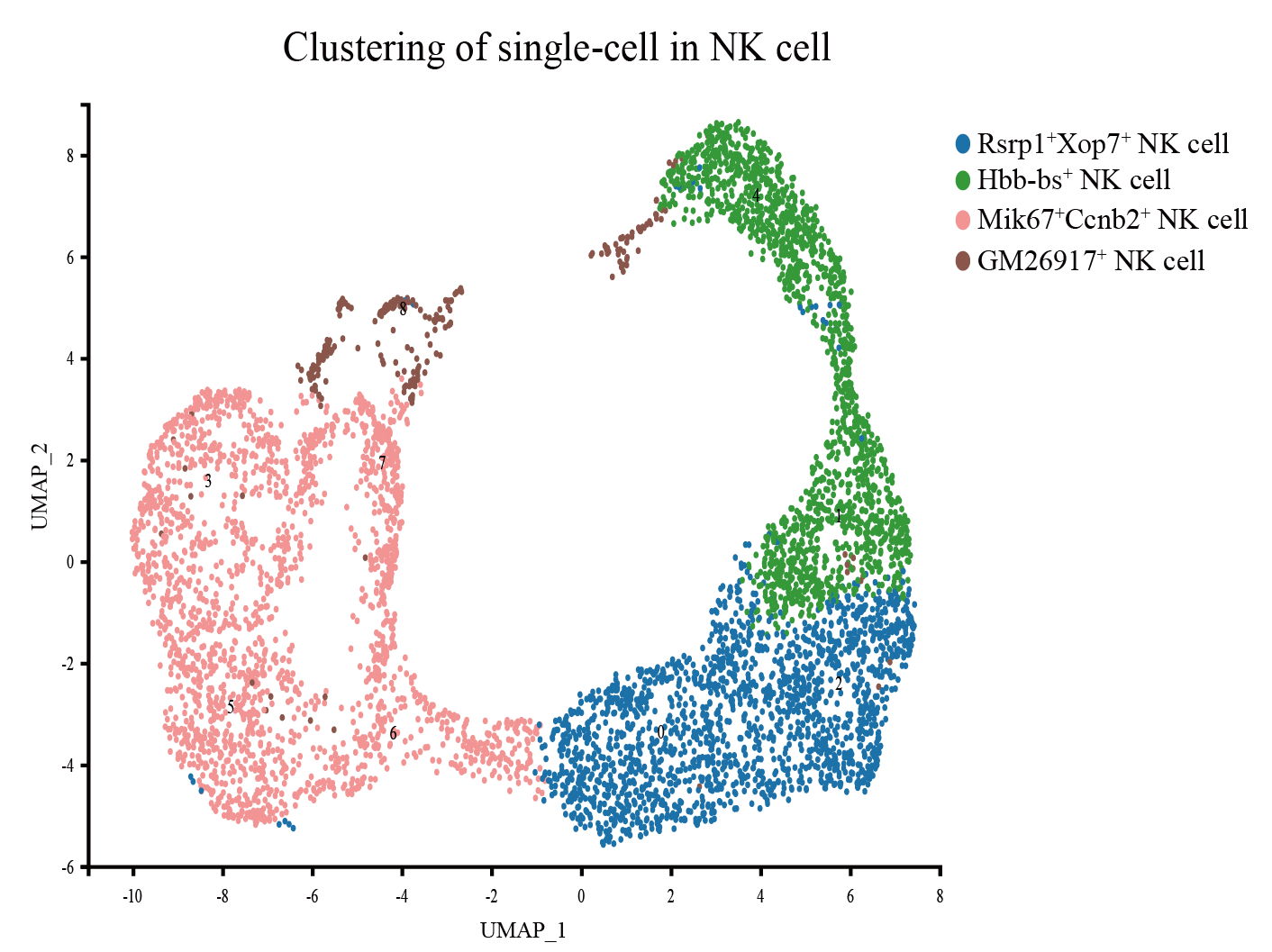


**Figure S14 Re-clustering of natural killer cells in single-cell sequencing.** UMAP plot showed natural killer cell subpopulations distribution in BTN3A1^KI^-Pristane-PBS (N=3) vs BTN3A1^KI^- Pristane-IL-38 group (50ug/kg IL-38, N=3).


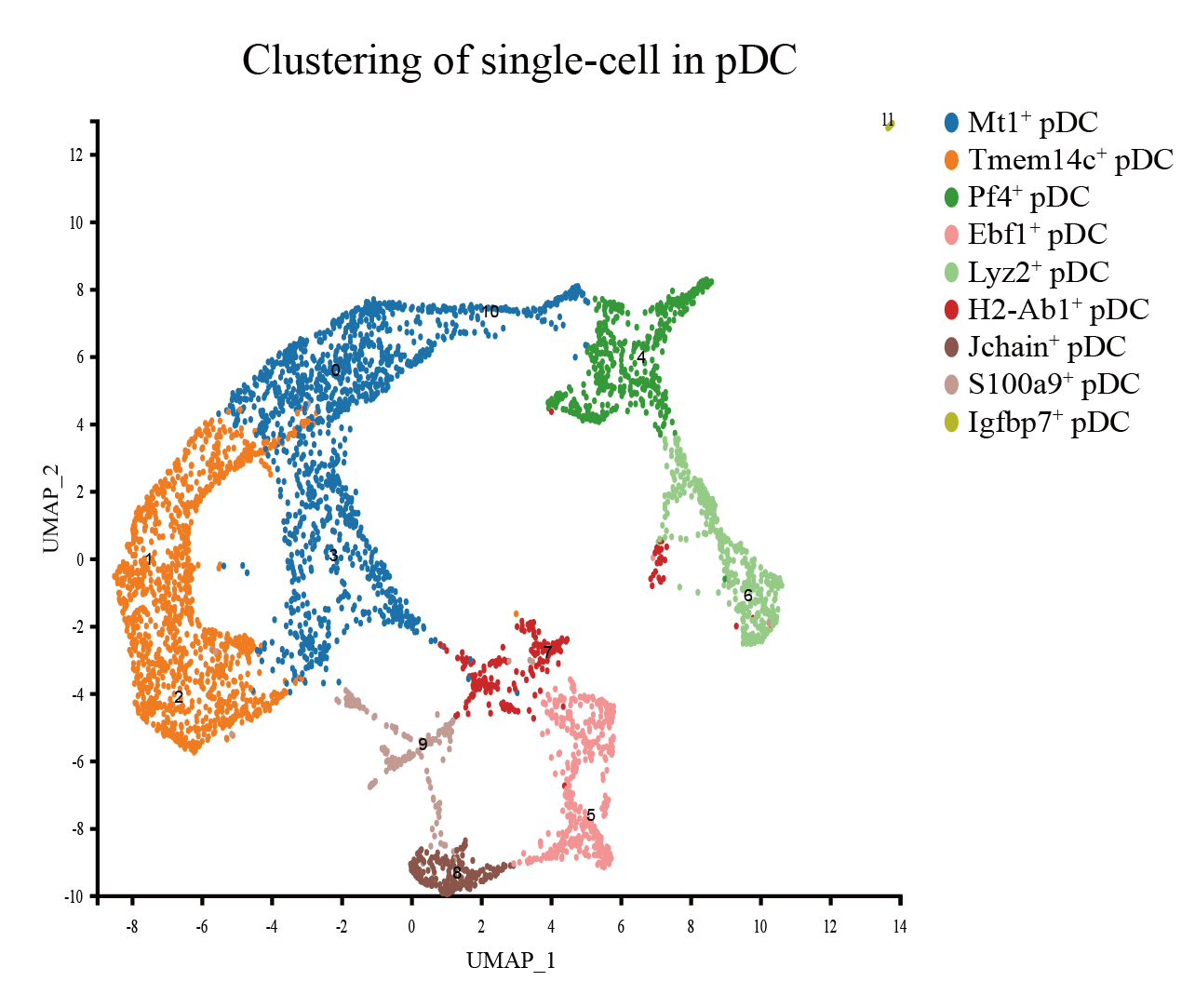


**Figure S15 Re-clustering of pDCs in single-cell sequencing.** UMAP plot showed plasmacytoid dendritic cells (pDCs) subpopulations distribution in BTN3A1^KI^-Pristane-PBS (N=3) vs BTN3A1^KI^-Pristane-IL-38 group (50ug/kg IL-38, N=3).


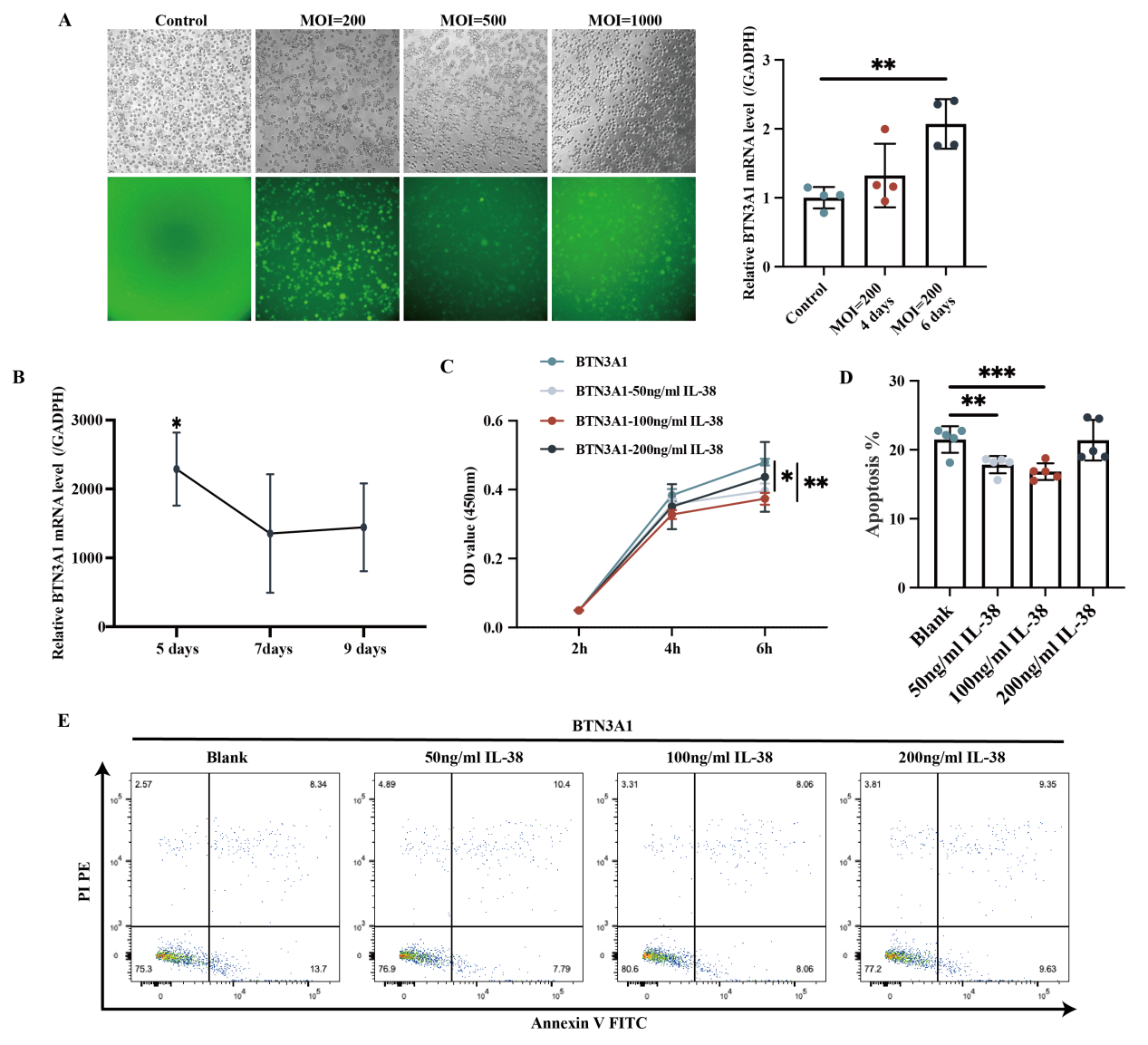


**Figure S16 BTN3A1 overexpressing adenovirus infection in wild-type mice and Jurkat T cells, and screening of optimal concentration of IL-38.** To find out optimal infection efficiency of the BTN3A1 overexpressing (BTN3A1^+/+^) adenovirus in wild-type (WT) mice and Jurkat T cells, we explored the amount and (or) time of virus infection. (A) Transfection of different doses of BTN3A1^+/+^ adenovirus in the Jurkat T cells (N=4), and different infection time by the GFP fluorescence intensity analysis. (B) Time-trend changes in mRNA expression of BTN3A1 in spleen of WT mice (N=3) injected with BTN3A1^+/+^ adenovirus. (C-E) Different concentrations of IL-38 treated naïve CD4^+^BTN3A1^+/+^ T cells. Naïve CD4^+^ T cells were isolated from WT mice injected with BTN3A1^+/+^ adenovirus, and then, were activated and stimulated with different doses of recombinant mouse IL-38 for detection of proliferation and apoptosis of CD4^+^ T cells (N=5). The cell proliferation was detected by CCK8 kits, and flow cytometry showed apoptosis of the cells. *P<0.05, **P<0.01, and ***P<0.001.


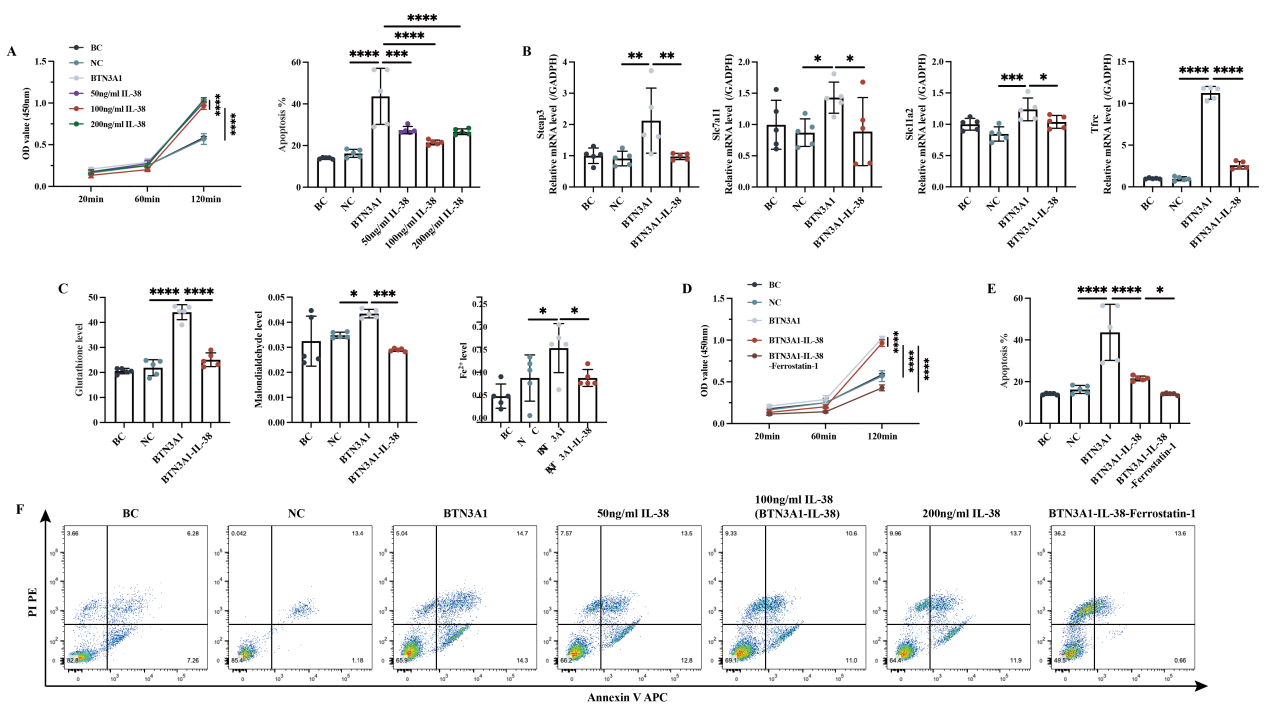


**Figure S17 BTN3A1 promotes Jurkat T cells proliferation and apoptosis by interacting with the IL-38-ferroptosis axis.** (A) Optimal concentration of recombinant human IL-38 protein screening determined by cell proliferation and apoptosis in *BTN3A1* gene overexpressed adenovirus transfected Jurkat T cells. Cell proliferation was detected by CCK8 kits. (B) The mRNA expression of ferroptosis-related proteins in the Jurkat T cells was detected by qRT-PCR. (C) Detection of Fe^2+^, glutathione and malondialdehyde levels in Jurkat cells reflects ferroptosis of Jurkat T cells. (D-E) Detection of proliferation and apoptosis of Jurkat T cells with/without the treatment of Ferrostatin-1. (F) Representative flowchart of Jurkat T cells apoptosis. BC, blank control. NC, adenovirus negative control. *P<0.05, **P<0.01, ***P<0.001 and ****P<0.0001.

**Supplementary Tables**

Table S1 The quantitative relationship between proteins and genes that can be associated in the two dimensions of quantitative and significant differences.

|  |  | Number of proteins | Number of genes | Number of correlations |
| --- | --- | --- | --- | --- |
| BTN3A1^KI^-PBS vs WT-PBS | Quantitative | 7816 | 16871 | 7675 |
|  | Differentially expressed | 56 | 1 | 0 |
| BTN3A1^KI^-Pristane vs BTN3A1^KI^-PBS | Quantitative | 7836 | 17272 | 7707 |
|  | Differentially expressed | 207 | 617 | 12 |
| BTN3A1^KI^-Pristane-IL-38 vs BTN3A1^KI^-Pristane-PBS | Quantitative | 7800 | 17217 | 7678 |
|  | Differentially expressed | 200 | 2001 | 39 |
| BTN3A1^KI^-Pristane-IL-38 vs BTN3A1^KI^-PBS | Quantitative | 7784 | 16799 | 7648 |
|  | Differentially expressed | 84 | 13 | 0 |

WT, wild-type.

| Table S2 KEGG enrichment-related pathway proteins and genes (BTN3A1^KI^-Pristane-IL-38 vs BTN3A1^KI^-Pristane-PBS). | | | | | | |
| --- | --- | --- | --- | --- | --- | --- |
| Pathway | Protein num | Gene num | Cor count | Protein P value | Gene P value | Cor mapID |
| Ferroptosis | 4 | 18 | 4 | 0.010 | 0.014 | P49282\|NRAM2,Q62351\|TFR1,Q8CI59\|STEA3,Q9WTR6\|XCT |
| Complement and coagulation cascades | 5 | 10 | 0 | 0.024 | 1.000 |  |
| p53 signaling pathway | 4 | 29 | 3 | 0.035 | 0.013 | O08586\|PTEN,P24860\|CCNB1,Q8CI59\|STEA3 |
| Tuberculosis | 7 | 64 | 4 | 0.053 | 0.011 | A2AIV8\|CARD9,P20491\|FCERG,Q61805\|LBP,Q9R1Q9\|VAS1 |
| Primary immunodeficiency | 3 | 22 | 1 | 0.106 | 0.000 | Q9WVS0\|ICOS |
| Platinum drug resistance | 3 | 32 | 1 | 0.132 | 0.011 | Q9CPU4\|MGST3 |
| Notch signaling pathway | 2 | 24 | 0 | 0.221 | 0.025 |  |
| Oxidative phosphorylation | 4 | 62 | 2 | 0.236 | 0.000 | P43024\|CX6A1,Q9R1Q9\|VAS1 |
| Hematopoietic cell lineage | 3 | 37 | 1 | 0.259 | 0.007 | Q62351\|TFR1 |
| mTOR signaling pathway | 4 | 54 | 2 | 0.273 | 0.032 | O08586\|PTEN,Q8CF66\|LTOR4 |
| Lysosome | 4 | 54 | 2 | 0.284 | 0.001 | P49282\|NRAM2,Q9R1Q9\|VAS1 |
| Phagosome | 5 | 62 | 2 | 0.302 | 0.025 | Q62351\|TFR1,Q9R1Q9\|VAS1 |
| PI3K-Akt signaling pathway | 7 | 115 | 1 | 0.341 | 0.034 | O08586\|PTEN |
| Longevity regulating pathway-worm | 2 | 44 | 1 | 0.371 | 0.000 | O08586\|PTEN |
| Lysine degradation | 2 | 26 | 1 | 0.413 | 0.016 | Q8BVE8\|NSD2 |
| EGFR tyrosine kinase inhibitor resistance | 2 | 31 | 2 | 0.430 | 0.016 | O08586\|PTEN,Q04690\|NF1 |
| Inositol phosphate metabolism | 2 | 32 | 2 | 0.438 | 0.002 | O08586\|PTEN,Q80XI4\|PI42B |
| RNA degradation | 2 | 41 | 2 | 0.478 | 0.000 | P21550\|ENOB,P62322\|LSM5 |
| Apoptosis-multiple species | 1 | 14 | 0 | 0.492 | 0.036 |  |
| Parkinson disease | 3 | 110 | 1 | 0.497 | 0.000 | P43024\|CX6A1 |
| Fanconi anemia pathway | 1 | 23 | 0 | 0.504 | 0.006 |  |
| Progesterone-mediated oocyte maturation | 2 | 38 | 1 | 0.508 | 0.003 | P24860\|CCNB1 |
| Cellular senescence | 3 | 71 | 3 | 0.532 | 0.001 | O08586\|PTEN,P24860\|CCNB1,Q64701\|RBL1 |
| Phosphatidylinositol signaling system | 2 | 43 | 2 | 0.538 | 0.000 | O08586\|PTEN,Q80XI4\|PI42B |
| Fc epsilon RI signaling pathway | 2 | 28 | 1 | 0.545 | 0.007 | P20491\|FCERG |
| ECM-receptor interaction | 2 | 33 | 0 | 0.552 | 0.027 |  |
| Ubiquitin-mediated proteolysis | 3 | 58 | 0 | 0.561 | 0.001 |  |
| Small cell lung cancer | 2 | 40 | 1 | 0.580 | 0.001 | O08586\|PTEN |
| FoxO signaling pathway | 2 | 61 | 2 | 0.606 | 0.000 | O08586\|PTEN,P24860\|CCNB1 |
| Cell cycle | 2 | 48 | 2 | 0.625 | 0.005 | P24860\|CCNB1,Q64701\|RBL1 |
| B cell receptor signaling pathway | 2 | 35 | 1 | 0.631 | 0.001 | Q9D103\|IFM1 |
| Sphingolipid signaling pathway | 2 | 46 | 2 | 0.655 | 0.013 | O08586\|PTEN,P20491\|FCERG |
| Retrograde endocannabinoid signaling | 2 | 51 | 0 | 0.667 | 0.016 |  |
| Thermogenesis | 4 | 106 | 1 | 0.670 | 0.000 | P43024\|CX6A1 |
| Fatty acid metabolism | 1 | 27 | 0 | 0.672 | 0.005 |  |
| Glycerolipid metabolism | 1 | 26 | 0 | 0.680 | 0.010 |  |
| NF-kappa B signaling pathway | 2 | 50 | 1 | 0.694 | 0.000 | Q61805\|LBP |
| Endometrial cancer | 1 | 23 | 1 | 0.695 | 0.031 | O08586\|PTEN |
| Glioma | 1 | 29 | 1 | 0.709 | 0.020 | O08586\|PTEN |
| MicroRNAs in cancer | 2 | 58 | 1 | 0.710 | 0.013 | O08586\|PTEN |
| Pathways in cancer | 6 | 171 | 2 | 0.747 | 0.021 | O08586\|PTEN,Q9CPU4\|MGST3 |
| Parathyroid hormone synthesis, secretion and action | 1 | 42 | 1 | 0.766 | 0.007 | Q01063\|PDE4D |
| Measles | 2 | 52 | 0 | 0.769 | 0.013 |  |
| Autophagy-animal | 2 | 57 | 1 | 0.773 | 0.001 | O08586\|PTEN |
| Prostate cancer | 1 | 37 | 1 | 0.783 | 0.021 | O08586\|PTEN |
| Platelet activation | 2 | 51 | 1 | 0.785 | 0.001 | P20491\|FCERG |
| Transcriptional misregulation in cancer | 2 | 72 | 1 | 0.789 | 0.036 | Q8BVE8\|NSD2 |
| Phospholipase D signaling pathway | 2 | 54 | 1 | 0.797 | 0.013 | P20491\|FCERG |
| Salmonella infection | 1 | 99 | 0 | 0.798 | 0.000 |  |
| Ribosome | 2 | 101 | 1 | 0.801 | 0.000 | P62855\|RS26 |
| Focal adhesion | 3 | 71 | 1 | 0.803 | 0.010 | O08586\|PTEN |
| Glycerophospholipid metabolism | 1 | 40 | 0 | 0.803 | 0.003 |  |
| Human immunodeficiency virus 1 infection | 3 | 86 | 1 | 0.815 | 0.002 | P24860\|CCNB1 |
| Hepatitis B | 2 | 71 | 1 | 0.815 | 0.000 | Q9R1Q9\|VAS1 |
| Leishmaniasis | 1 | 29 | 0 | 0.817 | 0.009 |  |
| Regulation of actin cytoskeleton | 3 | 76 | 1 | 0.820 | 0.014 | Q80XI4\|PI42B |
| Shigellosis | 1 | 103 | 0 | 0.830 | 0.000 |  |
| Kaposi sarcoma-associated herpesvirus infection | 2 | 74 | 0 | 0.834 | 0.034 |  |
| Toxoplasmosis | 1 | 44 | 0 | 0.890 | 0.003 |  |
| Apelin signaling pathway | 1 | 51 | 0 | 0.890 | 0.009 |  |
| Viral carcinogenesis | 2 | 92 | 2 | 0.895 | 0.000 | Q64701\|RBL1,Q9D0D5\|T2EA |
| Osteoclast differentiation | 1 | 50 | 0 | 0.896 | 0.001 |  |
| T cell receptor signaling pathway | 1 | 50 | 1 | 0.898 | 0.000 | Q9WVS0\|ICOS |
| Human cytomegalovirus infection | 2 | 93 | 0 | 0.917 | 0.001 |  |
| Fc gamma R-mediated phagocytosis | 1 | 39 | 0 | 0.934 | 0.002 |  |
| Epstein-Barr virus infection | 2 | 86 | 0 | 0.946 | 0.001 |  |
| Apoptosis | 1 | 52 | 0 | 0.949 | 0.004 |  |
| Influenza A | 1 | 57 | 0 | 0.950 | 0.046 |  |
| Alzheimer disease | 1 | 144 | 1 | 0.962 | 0.000 | P43024\|CX6A1 |
| Chemokine signaling pathway | 1 | 69 | 0 | 0.9644857 | 0.005889595 |  |
| Huntington disease | 1 | 125 | 1 | 0.9653481 | 4.93E-09 | P43024\|CX6A1 |
| Spliceosome | 1 | 67 | 1 | 0.9748343 | 1.22E-08 | P62322\|LSM5 |
| Human T-cell leukemia virus 1 infection | 1 | 98 | 1 | 0.9826129 | 2.64E-05 | O08586\|PTEN |
| Endocytosis | 2 | 97 | 1 | 0.9838341 | 0.001529838 | Q62351\|TFR1 |
| MAPK signaling pathway | 1 | 110 | 1 | 0.9888543 | 0.000130136 | Q04690\|NF1 |

Num, number; Cor, correlative; mTOR, mammalian target of rapamycin; PI3K-Akt, phosphatidylinositol-4,5-bisphosphate 3-kinase-protein kinase B; EGFR, epidermal growth factor receptor; ECM, extracellular matrix; FoxO, Fork head boxO; MAPK, mitogen-activated protein kinase.

| Table S3 KEGG enrichment-related pathway proteins and genes (BTN3A1^KI^-Pristane vs BTN3A1^KI^-PBS). | | | | | | |
| --- | --- | --- | --- | --- | --- | --- |
| Pathway | Protein num | Gene num | Cor count | P value (Protein) | P value (Gene) | Cor mapID |
| Amoebiasis | 12 | 8 | 1 | 0.000 | 0.307 | Q3UV74\|ITB2L |
| Staphylococcus aureus infection | 7 | 3 | 1 | 0.001 | 0.975 | Q3UV74\|ITB2L |
| Protein digestion and absorption | 7 | 5 | 1 | 0.001 | 0.787 | Q80X19\|COEA1 |
| Systemic lupus erythematosus | 7 | 6 | 0 | 0.002 | 0.889 |  |
| Tuberculosis | 10 | 12 | 1 | 0.003 | 0.401 | Q3UV74\|ITB2L |
| Autoimmune thyroid disease | 5 | 2 | 0 | 0.006 | 0.951 |  |
| Natural killer cell-mediated cytotoxicity | 7 | 9 | 1 | 0.008 | 0.232 | Q3UV74\|ITB2L |
| Leishmaniasis | 6 | 4 | 1 | 0.008 | 0.615 | Q3UV74\|ITB2L |
| ECM-receptor interaction | 6 | 5 | 0 | 0.013 | 0.618 |  |
| Ribosome | 8 | 9 | 0 | 0.013 | 0.487 |  |
| Renin-angiotensin system | 3 | 2 | 0 | 0.013 | 0.662 |  |
| Focal adhesion | 10 | 15 | 0 | 0.015 | 0.230 |  |
| Steroid hormone biosynthesis | 3 | 1 | 0 | 0.015 | 0.996 |  |
| Phagosome | 9 | 11 | 1 | 0.015 | 0.525 | Q3UV74\|ITB2L |
| Allograft rejection | 4 | 2 | 0 | 0.026 | 0.890 |  |
| Primary immunodeficiency | 4 | 2 | 0 | 0.032 | 0.647 |  |
| Transcriptional misregulation in cancer | 7 | 14 | 0 | 0.033 | 0.428 |  |
| Hematopoietic cell lineage | 5 | 5 | 0 | 0.039 | 0.667 |  |
| Neuroactive ligand-receptor interaction | 4 | 11 | 0 | 0.047 | 0.998 |  |
| Metabolism of xenobiotics by cytochrome P450 | 3 | 9 | 0 | 0.050 | 0.028 |  |
| Drug metabolism-cytochrome P450 | 2 | 9 | 0 | 0.157 | 0.024 |  |
| TGF-beta signaling pathway | 2 | 13 | 0 | 0.184 | 0.004 |  |
| Adherens junction | 3 | 9 | 0 | 0.294 | 0.026 |  |
| Relaxin signaling pathway | 3 | 13 | 0 | 0.445 | 0.047 |  |
| NF-kappa B signaling pathway | 3 | 12 | 0 | 0.465 | 0.024 |  |
| MicroRNAs in cancer | 3 | 16 | 0 | 0.485 | 0.035 |  |
| Regulation of actin cytoskeleton | 5 | 20 | 1 | 0.499 | 0.042 | Q3UV74\|ITB2L |
| Parkinson disease | 3 | 32 | 0 | 0.535 | 0.000 |  |
| Regulation of lipolysis in adipocytes | 1 | 7 | 0 | 0.548 | 0.050 |  |
| Notch signaling pathway | 1 | 8 | 0 | 0.613 | 0.026 |  |
| Cardiac muscle contraction | 1 | 13 | 0 | 0.651 | 0.001 |  |
| Alzheimer disease | 3 | 42 | 0 | 0.663 | 0.000 |  |
| Pathways in cancer | 7 | 44 | 0 | 0.663 | 0.025 |  |
| Circadian entrainment | 1 | 11 | 0 | 0.716 | 0.033 |  |
| Salivary secretion | 1 | 10 | 0 | 0.730 | 0.033 |  |
| Oxidative phosphorylation | 2 | 22 | 0 | 0.744 | 0.000 |  |
| Lysine degradation | 1 | 8 | 0 | 0.780 | 0.037 |  |
| Thermogenesis | 3 | 31 | 0 | 0.864 | 0.000 |  |
| Protein processing in endoplasmic reticulum | 2 | 21 | 0 | 0.895 | 0.002 |  |
| Apelin signaling pathway | 1 | 14 | 0 | 0.904 | 0.036 |  |
| Fluid shear stress and atherosclerosis | 1 | 15 | 0 | 0.918 | 0.028 |  |
| cGMP-PKG signaling pathway | 1 | 18 | 0 | 0.922 | 0.016 |  |
| Human T-cell leukemia virus 1 infection | 2 | 26 | 1 | 0.926 | 0.004 | Q3UV74\|ITB2L |
| Axon guidance | 1 | 17 | 0 | 0.936 | 0.045 |  |
| Huntington disease | 1 | 33 | 0 | 0.972 | 0.000 |  |

Num, number; Cor, correlative; ECM, extracellular matrix; TGF, transforming growth factor; cGMP-PKG, cyclic guanosine monophosphate-dependent protein kinase.

| Table S4 Marker genes for all immune cells and resources. | | | | | | | | |
| --- | --- | --- | --- | --- | --- | --- | --- | --- |
| Species type | Tissue type | Cell type | Cell name | Cell ontology ID | Gene | Marker resource | PMID | Weight |
| Mouse | Spleen | Normal cell | CD4^+^ T follicular helper cell | CL_0002038 | Nt5e | Experiment | 24030473 | 1 |
| Mouse | Spleen | Normal cell | CD4^+^ T follicular helper cell | CL_0002038 | Izumo1r | Experiment | 24030473 | 1 |
| Mouse | Spleen | Normal cell | T1 (Transitional) B cell | CL_0000958 | Cr2 | Experiment | 25015269 | 1 |
| Mouse | Spleen | Normal cell | T1 (Transitional) B cell | CL_0000958 | Fcer2a | Experiment | 25015269 | 1 |
| Mouse | Spleen | Normal cell | T1 (Transitional) B cell | CL_0000958 | Cd40lg | Experiment | 25015269 | 1 |
| Mouse | Spleen | Normal cell | Marginal zone B cell | CL_0000845 | Cr2 | Experiment | 25015269 | 1 |
| Mouse | Spleen | Normal cell | Marginal zone B cell | CL_0000845 | Fcer2a | Experiment | 25015269 | 1 |
| Mouse | Spleen | Normal cell | Marginal zone B cell | CL_0000845 | IgD family | Experiment | 25015269 | 1 |
| Mouse | Spleen | Normal cell | Marginal zone B cell | CL_0000845 | Cd40lg | Experiment | 25015269 | 1 |
| Mouse | Spleen | Normal cell | Germinal center B cell | CL_0000844 | Fasn | Experiment | 19716372 | 1 |
| Mouse | Spleen | Normal cell | Germinal center B cell | CL_0000844 | Prss57 | Experiment | 19716372 | 1 |
| Mouse | Spleen | Normal cell | Germinal center B cell | CL_0000844 | IgD family | Experiment | 19716372 | 1 |
| Mouse | Spleen | Normal cell | Regulatory T cell | CL_0000815 | Il2ra | Experiment | 26590312 | 1 |
| Mouse | Spleen | Normal cell | Regulatory T cell | CL_0000815 | Cd4 | Experiment | 26590312 | 1 |
| Mouse | Spleen | Normal cell | Regulatory T cell | CL_0000815 | Itga2 | Experiment | 26590312 | 1 |
| Mouse | Spleen | Normal cell | CD8^+^ T cell | CL_0000794 | Cd3d | Company | Company | 1 |
| Mouse | Spleen | Normal cell | CD8^+^ T cell | CL_0000794 | Cd3e | Company | Company | 1 |
| Mouse | Spleen | Normal cell | CD8^+^ T cell | CL_0000794 | Cd3g | Company | Company | 1 |
| Mouse | Spleen | Normal cell | CD8^+^ T cell | CL_0000794 | Ptprc | Company | Company | 1 |
| Mouse | Spleen | Normal cell | CD8^+^ T cell | CL_0000794 | Cd8a | Company | Company | 1 |
| Mouse | Spleen | Normal cell | CD4^+^CD25^+^ regulatory T cell | CL_0000792 | Il2ra | Company | Company | 1 |
| Mouse | Spleen | Normal cell | CD4^+^CD25^+^ regulatory T cell | CL_0000792 | Cd3d | Company | Company | 1 |
| Mouse | Spleen | Normal cell | CD4^+^CD25^+^ regulatory T cell | CL_0000792 | Cd3e | Company | Company | 1 |
| Mouse | Spleen | Normal cell | CD4^+^CD25^+^ regulatory T cell | CL_0000792 | Cd3g | Company | Company | 1 |
| Mouse | Spleen | Normal cell | CD4^+^CD25^+^ regulatory T cell | CL_0000792 | Cd4 | Company | Company | 1 |
| Mouse | Spleen | Normal cell | CD4^+^CD25^+^ regulatory T cell | CL_0000792 | Ptprc | Company | Company | 1 |
| Mouse | Spleen | Normal cell | CD4^+^CD25^+^ regulatory T cell | CL_0000792 | Nrp1 | Company | Company | 1 |
| Mouse | Spleen | Normal cell | Mature naive B cell | CL_0000788 | Cr2 | Experiment | 19716372 | 1 |
| Mouse | Spleen | Normal cell | Mature naive B cell | CL_0000788 | Fcer2a | Experiment | 19716372 | 1 |
| Mouse | Spleen | Normal cell | Mature naive B cell | CL_0000788 | Cd40lg | Experiment | 19716372 | 1 |
| Mouse | Spleen | Normal cell | Plasma cell | CL_0000786 | Sdc1 | Experiment | 19716372 | 1 |
| Mouse | Spleen | Normal cell | Plasmacytoid dendritic cell | CL_0000784 | Mcemp1 | Single-cell sequencing | 24531970 | 1 |
| Mouse | Spleen | Normal cell | Plasmacytoid dendritic cell | CL_0000784 | Cox14 | Single-cell sequencing | 24531970 | 1 |
| Mouse | Spleen | Normal cell | Plasmacytoid dendritic cell | CL_0000784 | Pagr1a | Single-cell sequencing | 24531970 | 1 |
| Mouse | Spleen | Normal cell | Plasmacytoid dendritic cell | CL_0000784 | Efcab14 | Single-cell sequencing | 24531970 | 1 |
| Mouse | Spleen | Normal cell | Plasmacytoid dendritic cell | CL_0000784 | Babam1 | Single-cell sequencing | 24531970 | 1 |
| Mouse | Spleen | Normal cell | Plasmacytoid dendritic cell | CL_0000784 | Acp5 | Single-cell sequencing | 24531970 | 1 |
| Mouse | Spleen | Normal cell | Plasmacytoid dendritic cell | CL_0000784 | Uqcc3 | Single-cell sequencing | 24531970 | 1 |
| Mouse | Spleen | Normal cell | Plasmacytoid dendritic cell | CL_0000784 | Akr1a1 | Single-cell sequencing | 24531970 | 1 |
| Mouse | Spleen | Normal cell | Plasmacytoid dendritic cell | CL_0000784 | Alas1 | Single-cell sequencing | 24531970 | 1 |
| Mouse | Spleen | Normal cell | Plasmacytoid dendritic cell | CL_0000784 | Ankrd13a | Single-cell sequencing | 24531970 | 1 |
| Mouse | Spleen | Normal cell | Plasmacytoid dendritic cell | CL_0000784 | Ano6 | Single-cell sequencing | 24531970 | 1 |
| Mouse | Spleen | Normal cell | Plasmacytoid dendritic cell | CL_0000784 | Arl6ip4 | Single-cell sequencing | 24531970 | 1 |
| Mouse | Spleen | Normal cell | Plasmacytoid dendritic cell | CL_0000784 | Atox1 | Single-cell sequencing | 24531970 | 1 |
| Mouse | Spleen | Normal cell | Plasmacytoid dendritic cell | CL_0000784 | Atp2a3 | Single-cell sequencing | 24531970 | 1 |
| Mouse | Spleen | Normal cell | Plasmacytoid dendritic cell | CL_0000784 | Atp5c1 | Single-cell sequencing | 24531970 | 1 |
| Mouse | Spleen | Normal cell | Plasmacytoid dendritic cell | CL_0000784 | Atp5j2 | Single-cell sequencing | 24531970 | 1 |
| Mouse | Spleen | Normal cell | Plasmacytoid dendritic cell | CL_0000784 | Dmac2 | Single-cell sequencing | 24531970 | 1 |
| Mouse | Spleen | Normal cell | Plasmacytoid dendritic cell | CL_0000784 | Atpif1 | Single-cell sequencing | 24531970 | 1 |
| Mouse | Spleen | Normal cell | Plasmacytoid dendritic cell | CL_0000784 | Avpi1 | Single-cell sequencing | 24531970 | 1 |
| Mouse | Spleen | Normal cell | Plasmacytoid dendritic cell | CL_0000784 | Dst | Single-cell sequencing | 24531970 | 1 |
| Mouse | Spleen | Normal cell | Plasmacytoid dendritic cell | CL_0000784 | C1qa | Single-cell sequencing | 24531970 | 1 |
| Mouse | Spleen | Normal cell | Plasmacytoid dendritic cell | CL_0000784 | C1qc | Single-cell sequencing | 24531970 | 1 |
| Mouse | Spleen | Normal cell | Plasmacytoid dendritic cell | CL_0000784 | Capns1 | Single-cell sequencing | 24531970 | 1 |
| Mouse | Spleen | Normal cell | Plasmacytoid dendritic cell | CL_0000784 | Cd68 | Single-cell sequencing | 24531970 | 1 |
| Mouse | Spleen | Normal cell | Plasmacytoid dendritic cell | CL_0000784 | Cd74 | Single-cell sequencing | 24531970 | 1 |
| Mouse | Spleen | Normal cell | Plasmacytoid dendritic cell | CL_0000784 | Cdc42ep3 | Single-cell sequencing | 24531970 | 1 |
| Mouse | Spleen | Normal cell | Plasmacytoid dendritic cell | CL_0000784 | Chchd3 | Single-cell sequencing | 24531970 | 1 |
| Mouse | Spleen | Normal cell | Plasmacytoid dendritic cell | CL_0000784 | Coro1b | Single-cell sequencing | 24531970 | 1 |
| Mouse | Spleen | Normal cell | Plasmacytoid dendritic cell | CL_0000784 | Coro7 | Single-cell sequencing | 24531970 | 1 |
| Mouse | Spleen | Normal cell | Plasmacytoid dendritic cell | CL_0000784 | Cstb | Single-cell sequencing | 24531970 | 1 |
| Mouse | Spleen | Normal cell | Plasmacytoid dendritic cell | CL_0000784 | Rsrp1 | Single-cell sequencing | 24531970 | 1 |
| Mouse | Spleen | Normal cell | Plasmacytoid dendritic cell | CL_0000784 | Dnajb9 | Single-cell sequencing | 24531970 | 1 |
| Mouse | Spleen | Normal cell | Plasmacytoid dendritic cell | CL_0000784 | Ehbp1l1 | Single-cell sequencing | 24531970 | 1 |
| Mouse | Spleen | Normal cell | Plasmacytoid dendritic cell | CL_0000784 | Eif3f | Single-cell sequencing | 24531970 | 1 |
| Mouse | Spleen | Normal cell | Plasmacytoid dendritic cell | CL_0000784 | Eif4ebp1 | Single-cell sequencing | 24531970 | 1 |
| Mouse | Spleen | Normal cell | Plasmacytoid dendritic cell | CL_0000784 | Elof1 | Single-cell sequencing | 24531970 | 1 |
| Mouse | Spleen | Normal cell | Plasmacytoid dendritic cell | CL_0000784 | Exoc2 | Single-cell sequencing | 24531970 | 1 |
| Mouse | Spleen | Normal cell | Plasmacytoid dendritic cell | CL_0000784 | Fam173a | Single-cell sequencing | 24531970 | 1 |
| Mouse | Spleen | Normal cell | Plasmacytoid dendritic cell | CL_0000784 | Fam32a | Single-cell sequencing | 24531970 | 1 |
| Mouse | Spleen | Normal cell | Plasmacytoid dendritic cell | CL_0000784 | Tpgs1 | Single-cell sequencing | 24531970 | 1 |
| Mouse | Spleen | Normal cell | Plasmacytoid dendritic cell | CL_0000784 | Gm5617 | Single-cell sequencing | 24531970 | 1 |
| Mouse | Spleen | Normal cell | Plasmacytoid dendritic cell | CL_0000784 | Gngt2 | Single-cell sequencing | 24531970 | 1 |
| Mouse | Spleen | Normal cell | Plasmacytoid dendritic cell | CL_0000784 | Gpx4 | Single-cell sequencing | 24531970 | 1 |
| Mouse | Spleen | Normal cell | Plasmacytoid dendritic cell | CL_0000784 | Gusb | Single-cell sequencing | 24531970 | 1 |
| Mouse | Spleen | Normal cell | Plasmacytoid dendritic cell | CL_0000784 | H2-Aa | Single-cell sequencing | 24531970 | 1 |
| Mouse | Spleen | Normal cell | Plasmacytoid dendritic cell | CL_0000784 | H2-Ab1 | Single-cell sequencing | 24531970 | 1 |
| Mouse | Spleen | Normal cell | Plasmacytoid dendritic cell | CL_0000784 | H2-Eb1 | Single-cell sequencing | 24531970 | 1 |
| Mouse | Spleen | Normal cell | Plasmacytoid dendritic cell | CL_0000784 | Haao | Single-cell sequencing | 24531970 | 1 |
| Mouse | Spleen | Normal cell | Plasmacytoid dendritic cell | CL_0000784 | Haus8 | Single-cell sequencing | 24531970 | 1 |
| Mouse | Spleen | Normal cell | Plasmacytoid dendritic cell | CL_0000784 | Hint1 | Single-cell sequencing | 24531970 | 1 |
| Mouse | Spleen | Normal cell | Plasmacytoid dendritic cell | CL_0000784 | Hsd17b10 | Single-cell sequencing | 24531970 | 1 |
| Mouse | Spleen | Normal cell | Plasmacytoid dendritic cell | CL_0000784 | Idh2 | Single-cell sequencing | 24531970 | 1 |
| Mouse | Spleen | Normal cell | Plasmacytoid dendritic cell | CL_0000784 | Ifi30 | Single-cell sequencing | 24531970 | 1 |
| Mouse | Spleen | Normal cell | Plasmacytoid dendritic cell | CL_0000784 | Il10rb | Single-cell sequencing | 24531970 | 1 |
| Mouse | Spleen | Normal cell | Plasmacytoid dendritic cell | CL_0000784 | Il12b | Single-cell sequencing | 24531970 | 1 |
| Mouse | Spleen | Normal cell | Plasmacytoid dendritic cell | CL_0000784 | Inpp4a | Single-cell sequencing | 24531970 | 1 |
| Mouse | Spleen | Normal cell | Plasmacytoid dendritic cell | CL_0000784 | Ip6k1 | Single-cell sequencing | 24531970 | 1 |
| Mouse | Spleen | Normal cell | Plasmacytoid dendritic cell | CL_0000784 | Itgax | Single-cell sequencing | 24531970 | 1 |
| Mouse | Spleen | Normal cell | Plasmacytoid dendritic cell | CL_0000784 | Itm2b | Single-cell sequencing | 24531970 | 1 |
| Mouse | Spleen | Normal cell | Plasmacytoid dendritic cell | CL_0000784 | Lamp1 | Single-cell sequencing | 24531970 | 1 |
| Mouse | Spleen | Normal cell | Plasmacytoid dendritic cell | CL_0000784 | Map1lc3b | Single-cell sequencing | 24531970 | 1 |
| Mouse | Spleen | Normal cell | Plasmacytoid dendritic cell | CL_0000784 | Mcm6 | Single-cell sequencing | 24531970 | 1 |
| Mouse | Spleen | Normal cell | Plasmacytoid dendritic cell | CL_0000784 | Mdh2 | Single-cell sequencing | 24531970 | 1 |
| Mouse | Spleen | Normal cell | Plasmacytoid dendritic cell | CL_0000784 | Mrpl34 | Single-cell sequencing | 24531970 | 1 |
| Mouse | Spleen | Normal cell | Plasmacytoid dendritic cell | CL_0000784 | Ms4a7 | Single-cell sequencing | 24531970 | 1 |
| Mouse | Spleen | Normal cell | Plasmacytoid dendritic cell | CL_0000784 | Ncor1 | Single-cell sequencing | 24531970 | 1 |
| Mouse | Spleen | Normal cell | Plasmacytoid dendritic cell | CL_0000784 | Ndufa10 | Single-cell sequencing | 24531970 | 1 |
| Mouse | Spleen | Normal cell | Plasmacytoid dendritic cell | CL_0000784 | Ndufa9 | Single-cell sequencing | 24531970 | 1 |
| Mouse | Spleen | Normal cell | Plasmacytoid dendritic cell | CL_0000784 | Ndufb8 | Single-cell sequencing | 24531970 | 1 |
| Mouse | Spleen | Normal cell | Plasmacytoid dendritic cell | CL_0000784 | Neurl3 | Single-cell sequencing | 24531970 | 1 |
| Mouse | Spleen | Normal cell | Plasmacytoid dendritic cell | CL_0000784 | Nudt3 | Single-cell sequencing | 24531970 | 1 |
| Mouse | Spleen | Normal cell | Plasmacytoid dendritic cell | CL_0000784 | P4hb | Single-cell sequencing | 24531970 | 1 |
| Mouse | Spleen | Normal cell | Plasmacytoid dendritic cell | CL_0000784 | Pip5k1c | Single-cell sequencing | 24531970 | 1 |
| Mouse | Spleen | Normal cell | Plasmacytoid dendritic cell | CL_0000784 | Pisd | Single-cell sequencing | 24531970 | 1 |
| Mouse | Spleen | Normal cell | Plasmacytoid dendritic cell | CL_0000784 | Pkn1 | Single-cell sequencing | 24531970 | 1 |
| Mouse | Spleen | Normal cell | Plasmacytoid dendritic cell | CL_0000784 | Pkp3 | Single-cell sequencing | 24531970 | 1 |
| Mouse | Spleen | Normal cell | Plasmacytoid dendritic cell | CL_0000784 | Plaur | Single-cell sequencing | 24531970 | 1 |
| Mouse | Spleen | Normal cell | Plasmacytoid dendritic cell | CL_0000784 | Plod3 | Single-cell sequencing | 24531970 | 1 |
| Mouse | Spleen | Normal cell | Plasmacytoid dendritic cell | CL_0000784 | Plxnb2 | Single-cell sequencing | 24531970 | 1 |
| Mouse | Spleen | Normal cell | Plasmacytoid dendritic cell | CL_0000784 | Plxnd1 | Single-cell sequencing | 24531970 | 1 |
| Mouse | Spleen | Normal cell | Plasmacytoid dendritic cell | CL_0000784 | Ppp1r12c | Single-cell sequencing | 24531970 | 1 |
| Mouse | Spleen | Normal cell | Plasmacytoid dendritic cell | CL_0000784 | Pqlc1 | Single-cell sequencing | 24531970 | 1 |
| Mouse | Spleen | Normal cell | Plasmacytoid dendritic cell | CL_0000784 | Psen2 | Single-cell sequencing | 24531970 | 1 |
| Mouse | Spleen | Normal cell | Plasmacytoid dendritic cell | CL_0000784 | Psmc4 | Single-cell sequencing | 24531970 | 1 |
| Mouse | Spleen | Normal cell | Plasmacytoid dendritic cell | CL_0000784 | Ptcd2 | Single-cell sequencing | 24531970 | 1 |
| Mouse | Spleen | Normal cell | Plasmacytoid dendritic cell | CL_0000784 | Pycr2 | Single-cell sequencing | 24531970 | 1 |
| Mouse | Spleen | Normal cell | Plasmacytoid dendritic cell | CL_0000784 | Qdpr | Single-cell sequencing | 24531970 | 1 |
| Mouse | Spleen | Normal cell | Plasmacytoid dendritic cell | CL_0000784 | Rasa3 | Single-cell sequencing | 24531970 | 1 |
| Mouse | Spleen | Normal cell | Plasmacytoid dendritic cell | CL_0000784 | Rbbp7 | Single-cell sequencing | 24531970 | 1 |
| Mouse | Spleen | Normal cell | Plasmacytoid dendritic cell | CL_0000784 | Rbm25 | Single-cell sequencing | 24531970 | 1 |
| Mouse | Spleen | Normal cell | Plasmacytoid dendritic cell | CL_0000784 | Rogdi | Single-cell sequencing | 24531970 | 1 |
| Mouse | Spleen | Normal cell | Plasmacytoid dendritic cell | CL_0000784 | Rplp0 | Single-cell sequencing | 24531970 | 1 |
| Mouse | Spleen | Normal cell | Plasmacytoid dendritic cell | CL_0000784 | Rplp1 | Single-cell sequencing | 24531970 | 1 |
| Mouse | Spleen | Normal cell | Plasmacytoid dendritic cell | CL_0000784 | Rps20 | Single-cell sequencing | 24531970 | 1 |
| Mouse | Spleen | Normal cell | Plasmacytoid dendritic cell | CL_0000784 | Rxra | Single-cell sequencing | 24531970 | 1 |
| Mouse | Spleen | Normal cell | Plasmacytoid dendritic cell | CL_0000784 | Sae1 | Single-cell sequencing | 24531970 | 1 |
| Mouse | Spleen | Normal cell | Plasmacytoid dendritic cell | CL_0000784 | Scamp4 | Single-cell sequencing | 24531970 | 1 |
| Mouse | Spleen | Normal cell | Plasmacytoid dendritic cell | CL_0000784 | Senp6 | Single-cell sequencing | 24531970 | 1 |
| Mouse | Spleen | Normal cell | Plasmacytoid dendritic cell | CL_0000784 | Slc8b1 | Single-cell sequencing | 24531970 | 1 |
| Mouse | Spleen | Normal cell | Plasmacytoid dendritic cell | CL_0000784 | Slc29a3 | Single-cell sequencing | 24531970 | 1 |
| Mouse | Spleen | Normal cell | Plasmacytoid dendritic cell | CL_0000784 | Slc43a2 | Single-cell sequencing | 24531970 | 1 |
| Mouse | Spleen | Normal cell | Plasmacytoid dendritic cell | CL_0000784 | Slc44a2 | Single-cell sequencing | 24531970 | 1 |
| Mouse | Spleen | Normal cell | Plasmacytoid dendritic cell | CL_0000784 | Slc9a3r1 | Single-cell sequencing | 24531970 | 1 |
| Mouse | Spleen | Normal cell | Plasmacytoid dendritic cell | CL_0000784 | Snrpd3 | Single-cell sequencing | 24531970 | 1 |
| Mouse | Spleen | Normal cell | Plasmacytoid dendritic cell | CL_0000784 | Snrpf | Single-cell sequencing | 24531970 | 1 |
| Mouse | Spleen | Normal cell | Plasmacytoid dendritic cell | CL_0000784 | Spns3 | Single-cell sequencing | 24531970 | 1 |
| Mouse | Spleen | Normal cell | Plasmacytoid dendritic cell | CL_0000784 | Srp14 | Single-cell sequencing | 24531970 | 1 |
| Mouse | Spleen | Normal cell | Plasmacytoid dendritic cell | CL_0000784 | Ssrp1 | Single-cell sequencing | 24531970 | 1 |
| Mouse | Spleen | Normal cell | Plasmacytoid dendritic cell | CL_0000784 | Syf2 | Single-cell sequencing | 24531970 | 1 |
| Mouse | Spleen | Normal cell | Plasmacytoid dendritic cell | CL_0000784 | Tfeb | Single-cell sequencing | 24531970 | 1 |
| Mouse | Spleen | Normal cell | Plasmacytoid dendritic cell | CL_0000784 | Tmem176a | Single-cell sequencing | 24531970 | 1 |
| Mouse | Spleen | Normal cell | Plasmacytoid dendritic cell | CL_0000784 | Tmem176b | Single-cell sequencing | 24531970 | 1 |
| Mouse | Spleen | Normal cell | Plasmacytoid dendritic cell | CL_0000784 | Tspan14 | Single-cell sequencing | 24531970 | 1 |
| Mouse | Spleen | Normal cell | Plasmacytoid dendritic cell | CL_0000784 | Ube2j1 | Single-cell sequencing | 24531970 | 1 |
| Mouse | Spleen | Normal cell | Plasmacytoid dendritic cell | CL_0000784 | Usp48 | Single-cell sequencing | 24531970 | 1 |
| Mouse | Spleen | Normal cell | Plasmacytoid dendritic cell | CL_0000784 | Vac14 | Single-cell sequencing | 24531970 | 1 |
| Mouse | Spleen | Normal cell | Plasmacytoid dendritic cell | CL_0000784 | Bud23 | Single-cell sequencing | 24531970 | 1 |
| Mouse | Spleen | Normal cell | Plasmacytoid dendritic cell | CL_0000784 | Wdr89 | Single-cell sequencing | 24531970 | 1 |
| Mouse | Spleen | Normal cell | Plasmacytoid dendritic cell | CL_0000784 | Ywhah | Single-cell sequencing | 24531970 | 1 |
| Mouse | Spleen | Normal cell | Plasmacytoid dendritic cell | CL_0000784 | Zbtb7a | Single-cell sequencing | 24531970 | 1 |
| Mouse | Spleen | Normal cell | Plasmacytoid dendritic cell | CL_0000784 | Zcchc24 | Single-cell sequencing | 24531970 | 1 |
| Mouse | Spleen | Normal cell | Plasmacytoid dendritic cell | CL_0000784 | Zeb2 | Single-cell sequencing | 24531970 | 1 |
| Mouse | Spleen | Normal cell | Neutrophil | CL_0000775 | Ly6g | Experiment | 22213571 | 1 |
| Mouse | Spleen | Normal cell | Natural killer cell | CL_0000623 | Klrb1c | Company | Company | 1 |
| Mouse | Spleen | Normal cell | Natural killer cell | CL_0000623 | Ncr1 | Company | Company | 1 |
| Mouse | Spleen | Normal cell | Natural killer T cell | CL_0000623 | Fcgr3 | Company | Company | 1 |
| Mouse | Spleen | Normal cell | Natural killer T cell | CL_0000623 | Cd3d | Company | Company | 1 |
| Mouse | Spleen | Normal cell | Natural killer T cell | CL_0000623 | Cd3e | Company | Company | 1 |
| Mouse | Spleen | Normal cell | Natural killer T cell | CL_0000623 | Cd3g | Company | Company | 1 |
| Mouse | Spleen | Normal cell | Natural killer T cell | CL_0000623 | Ptprc | Company | Company | 1 |
| Mouse | Spleen | Normal cell | Natural killer T cell | CL_0000623 | Ncam1 | Company | Company | 1 |
| Mouse | Spleen | Normal cell | Natural killer T cell | CL_0000623 | Klrb1c | Company | Company | 1 |
| Mouse | Spleen | Normal cell | Natural killer cell | CL_0000623 | 2310011J03Rik | Single-cell sequencing | 24531970 | 1 |
| Mouse | Spleen | Normal cell | Natural killer cell | CL_0000623 | 2610001J05Rik | Single-cell sequencing | 24531970 | 1 |
| Mouse | Spleen | Normal cell | Natural killer cell | CL_0000623 | Acot7 | Single-cell sequencing | 24531970 | 1 |
| Mouse | Spleen | Normal cell | Natural killer cell | CL_0000623 | Adam19 | Single-cell sequencing | 24531970 | 1 |
| Mouse | Spleen | Normal cell | Natural killer cell | CL_0000623 | Agpat3 | Single-cell sequencing | 24531970 | 1 |
| Mouse | Spleen | Normal cell | Natural killer cell | CL_0000623 | Anapc4 | Single-cell sequencing | 24531970 | 1 |
| Mouse | Spleen | Normal cell | Natural killer cell | CL_0000623 | Arf3 | Single-cell sequencing | 24531970 | 1 |
| Mouse | Spleen | Normal cell | Natural killer cell | CL_0000623 | Atp6v0a2 | Single-cell sequencing | 24531970 | 1 |
| Mouse | Spleen | Normal cell | Natural killer cell | CL_0000623 | Auh | Single-cell sequencing | 24531970 | 1 |
| Mouse | Spleen | Normal cell | Natural killer cell | CL_0000623 | Baiap3 | Single-cell sequencing | 24531970 | 1 |
| Mouse | Spleen | Normal cell | Natural killer cell | CL_0000623 | BC005624 | Single-cell sequencing | 24531970 | 1 |
| Mouse | Spleen | Normal cell | Natural killer cell | CL_0000623 | Capg | Single-cell sequencing | 24531970 | 1 |
| Mouse | Spleen | Normal cell | Natural killer cell | CL_0000623 | Cd27 | Single-cell sequencing | 24531970 | 1 |
| Mouse | Spleen | Normal cell | Natural killer cell | CL_0000623 | Cd300c | Single-cell sequencing | 24531970 | 1 |
| Mouse | Spleen | Normal cell | Natural killer cell | CL_0000623 | Cd4 | Single-cell sequencing | 24531970 | 1 |
| Mouse | Spleen | Normal cell | Natural killer cell | CL_0000623 | Cd6 | Single-cell sequencing | 24531970 | 1 |
| Mouse | Spleen | Normal cell | Natural killer cell | CL_0000623 | Cd72 | Single-cell sequencing | 24531970 | 1 |
| Mouse | Spleen | Normal cell | Natural killer cell | CL_0000623 | Cd8a | Single-cell sequencing | 24531970 | 1 |
| Mouse | Spleen | Normal cell | Natural killer cell | CL_0000623 | Chd8 | Single-cell sequencing | 24531970 | 1 |
| Mouse | Spleen | Normal cell | Natural killer cell | CL_0000623 | Clec10a | Single-cell sequencing | 24531970 | 1 |
| Mouse | Spleen | Normal cell | Natural killer cell | CL_0000623 | Cnot8 | Single-cell sequencing | 24531970 | 1 |
| Mouse | Spleen | Normal cell | Natural killer cell | CL_0000623 | Cyth4 | Single-cell sequencing | 24531970 | 1 |
| Mouse | Spleen | Normal cell | Natural killer cell | CL_0000623 | Dalrd3 | Single-cell sequencing | 24531970 | 1 |
| Mouse | Spleen | Normal cell | Natural killer cell | CL_0000623 | Ddx28 | Single-cell sequencing | 24531970 | 1 |
| Mouse | Spleen | Normal cell | Natural killer cell | CL_0000623 | Ddx50 | Single-cell sequencing | 24531970 | 1 |
| Mouse | Spleen | Normal cell | Natural killer cell | CL_0000623 | Dennd1c | Single-cell sequencing | 24531970 | 1 |
| Mouse | Spleen | Normal cell | Natural killer cell | CL_0000623 | Dera | Single-cell sequencing | 24531970 | 1 |
| Mouse | Spleen | Normal cell | Natural killer cell | CL_0000623 | Dgka | Single-cell sequencing | 24531970 | 1 |
| Mouse | Spleen | Normal cell | Natural killer cell | CL_0000623 | Dis3l2 | Single-cell sequencing | 24531970 | 1 |
| Mouse | Spleen | Normal cell | Natural killer cell | CL_0000623 | Dtx1 | Single-cell sequencing | 24531970 | 1 |
| Mouse | Spleen | Normal cell | Natural killer cell | CL_0000623 | Efhd2 | Single-cell sequencing | 24531970 | 1 |
| Mouse | Spleen | Normal cell | Natural killer cell | CL_0000623 | Eif3l | Single-cell sequencing | 24531970 | 1 |
| Mouse | Spleen | Normal cell | Natural killer cell | CL_0000623 | Elk4 | Single-cell sequencing | 24531970 | 1 |
| Mouse | Spleen | Normal cell | Natural killer cell | CL_0000623 | Emg1 | Single-cell sequencing | 24531970 | 1 |
| Mouse | Spleen | Normal cell | Natural killer cell | CL_0000623 | Epsti1 | Single-cell sequencing | 24531970 | 1 |
| Mouse | Spleen | Normal cell | Natural killer cell | CL_0000623 | Ercc3 | Single-cell sequencing | 24531970 | 1 |
| Mouse | Spleen | Normal cell | Natural killer cell | CL_0000623 | Faf2 | Single-cell sequencing | 24531970 | 1 |
| Mouse | Spleen | Normal cell | Natural killer cell | CL_0000623 | Fam117a | Single-cell sequencing | 24531970 | 1 |
| Mouse | Spleen | Normal cell | Natural killer cell | CL_0000623 | Fbxo7 | Single-cell sequencing | 24531970 | 1 |
| Mouse | Spleen | Normal cell | Natural killer cell | CL_0000623 | Ffar2 | Single-cell sequencing | 24531970 | 1 |
| Mouse | Spleen | Normal cell | Natural killer cell | CL_0000623 | Galnt2 | Single-cell sequencing | 24531970 | 1 |
| Mouse | Spleen | Normal cell | Natural killer cell | CL_0000623 | Ganab | Single-cell sequencing | 24531970 | 1 |
| Mouse | Spleen | Normal cell | Natural killer cell | CL_0000623 | Gfm1 | Single-cell sequencing | 24531970 | 1 |
| Mouse | Spleen | Normal cell | Natural killer cell | CL_0000623 | Gimap1 | Single-cell sequencing | 24531970 | 1 |
| Mouse | Spleen | Normal cell | Natural killer cell | CL_0000623 | Gstt2 | Single-cell sequencing | 24531970 | 1 |
| Mouse | Spleen | Normal cell | Natural killer cell | CL_0000623 | Gtf2a2 | Single-cell sequencing | 24531970 | 1 |
| Mouse | Spleen | Normal cell | Natural killer cell | CL_0000623 | H2-M2 | Single-cell sequencing | 24531970 | 1 |
| Mouse | Spleen | Normal cell | Natural killer cell | CL_0000623 | Hexb | Single-cell sequencing | 24531970 | 1 |
| Mouse | Spleen | Normal cell | Natural killer cell | CL_0000623 | Hopx | Single-cell sequencing | 24531970 | 1 |
| Mouse | Spleen | Normal cell | Natural killer cell | CL_0000623 | Ikzf3 | Single-cell sequencing | 24531970 | 1 |
| Mouse | Spleen | Normal cell | Natural killer cell | CL_0000623 | Ipo11 | Single-cell sequencing | 24531970 | 1 |
| Mouse | Spleen | Normal cell | Natural killer cell | CL_0000623 | Kctd10 | Single-cell sequencing | 24531970 | 1 |
| Mouse | Spleen | Normal cell | Natural killer cell | CL_0000623 | Kif21b | Single-cell sequencing | 24531970 | 1 |
| Mouse | Spleen | Normal cell | Natural killer cell | CL_0000623 | Lars2 | Single-cell sequencing | 24531970 | 1 |
| Mouse | Spleen | Normal cell | Natural killer cell | CL_0000623 | Lmo4 | Single-cell sequencing | 24531970 | 1 |
| Mouse | Spleen | Normal cell | Natural killer cell | CL_0000623 | Lrrc47 | Single-cell sequencing | 24531970 | 1 |
| Mouse | Spleen | Normal cell | Natural killer cell | CL_0000623 | Mad2l1bp | Single-cell sequencing | 24531970 | 1 |
| Mouse | Spleen | Normal cell | Natural killer cell | CL_0000623 | Nfatc3 | Single-cell sequencing | 24531970 | 1 |
| Mouse | Spleen | Normal cell | Natural killer cell | CL_0000623 | Nin | Single-cell sequencing | 24531970 | 1 |
| Mouse | Spleen | Normal cell | Natural killer cell | CL_0000623 | Nrbp1 | Single-cell sequencing | 24531970 | 1 |
| Mouse | Spleen | Normal cell | Natural killer cell | CL_0000623 | Otud5 | Single-cell sequencing | 24531970 | 1 |
| Mouse | Spleen | Normal cell | Natural killer cell | CL_0000623 | Parp3 | Single-cell sequencing | 24531970 | 1 |
| Mouse | Spleen | Normal cell | Natural killer cell | CL_0000623 | Pcbp2 | Single-cell sequencing | 24531970 | 1 |
| Mouse | Spleen | Normal cell | Natural killer cell | CL_0000623 | Pik3cd | Single-cell sequencing | 24531970 | 1 |
| Mouse | Spleen | Normal cell | Natural killer cell | CL_0000623 | Plec | Single-cell sequencing | 24531970 | 1 |
| Mouse | Spleen | Normal cell | Natural killer cell | CL_0000623 | Pnrc1 | Single-cell sequencing | 24531970 | 1 |
| Mouse | Spleen | Normal cell | Natural killer cell | CL_0000623 | Pop4 | Single-cell sequencing | 24531970 | 1 |
| Mouse | Spleen | Normal cell | Natural killer cell | CL_0000623 | Ppp1r14b | Single-cell sequencing | 24531970 | 1 |
| Mouse | Spleen | Normal cell | Natural killer cell | CL_0000623 | Prpf6 | Single-cell sequencing | 24531970 | 1 |
| Mouse | Spleen | Normal cell | Natural killer cell | CL_0000623 | Prr14 | Single-cell sequencing | 24531970 | 1 |
| Mouse | Spleen | Normal cell | Natural killer cell | CL_0000623 | Psmc1 | Single-cell sequencing | 24531970 | 1 |
| Mouse | Spleen | Normal cell | Natural killer cell | CL_0000623 | Rab43 | Single-cell sequencing | 24531970 | 1 |
| Mouse | Spleen | Normal cell | Natural killer cell | CL_0000623 | Rad50 | Single-cell sequencing | 24531970 | 1 |
| Mouse | Spleen | Normal cell | Natural killer cell | CL_0000623 | Raf1 | Single-cell sequencing | 24531970 | 1 |
| Mouse | Spleen | Normal cell | Natural killer cell | CL_0000623 | Rhof | Single-cell sequencing | 24531970 | 1 |
| Mouse | Spleen | Normal cell | Natural killer cell | CL_0000623 | Riok3 | Single-cell sequencing | 24531970 | 1 |
| Mouse | Spleen | Normal cell | Natural killer cell | CL_0000623 | Rnf115 | Single-cell sequencing | 24531970 | 1 |
| Mouse | Spleen | Normal cell | Natural killer cell | CL_0000623 | Sars | Single-cell sequencing | 24531970 | 1 |
| Mouse | Spleen | Normal cell | Natural killer cell | CL_0000623 | Scaf1 | Single-cell sequencing | 24531970 | 1 |
| Mouse | Spleen | Normal cell | Natural killer cell | CL_0000623 | Scamp3 | Single-cell sequencing | 24531970 | 1 |
| Mouse | Spleen | Normal cell | Natural killer cell | CL_0000623 | Scand1 | Single-cell sequencing | 24531970 | 1 |
| Mouse | Spleen | Normal cell | Natural killer cell | CL_0000623 | Sdhc | Single-cell sequencing | 24531970 | 1 |
| Mouse | Spleen | Normal cell | Natural killer cell | CL_0000623 | Selplg | Single-cell sequencing | 24531970 | 1 |
| Mouse | Spleen | Normal cell | Natural killer cell | CL_0000623 | Sema4d | Single-cell sequencing | 24531970 | 1 |
| Mouse | Spleen | Normal cell | Natural killer cell | CL_0000623 | Smap1 | Single-cell sequencing | 24531970 | 1 |
| Mouse | Spleen | Normal cell | Natural killer cell | CL_0000623 | Smarca2 | Single-cell sequencing | 24531970 | 1 |
| Mouse | Spleen | Normal cell | Natural killer cell | CL_0000623 | Snrnp200 | Single-cell sequencing | 24531970 | 1 |
| Mouse | Spleen | Normal cell | Natural killer cell | CL_0000623 | Spn | Single-cell sequencing | 24531970 | 1 |
| Mouse | Spleen | Normal cell | Natural killer cell | CL_0000623 | Ssu72 | Single-cell sequencing | 24531970 | 1 |
| Mouse | Spleen | Normal cell | Natural killer cell | CL_0000623 | Sulf2 | Single-cell sequencing | 24531970 | 1 |
| Mouse | Spleen | Normal cell | Natural killer cell | CL_0000623 | Tcra | Single-cell sequencing | 24531970 | 1 |
| Mouse | Spleen | Normal cell | Natural killer cell | CL_0000623 | Thy1 | Single-cell sequencing | 24531970 | 1 |
| Mouse | Spleen | Normal cell | Natural killer cell | CL_0000623 | Trpc4ap | Single-cell sequencing | 24531970 | 1 |
| Mouse | Spleen | Normal cell | Natural killer cell | CL_0000623 | Tspan31 | Single-cell sequencing | 24531970 | 1 |
| Mouse | Spleen | Normal cell | Natural killer cell | CL_0000623 | Urod | Single-cell sequencing | 24531970 | 1 |
| Mouse | Spleen | Normal cell | Natural killer cell | CL_0000623 | Uso1 | Single-cell sequencing | 24531970 | 1 |
| Mouse | Spleen | Normal cell | Natural killer cell | CL_0000623 | Vapa | Single-cell sequencing | 24531970 | 1 |
| Mouse | Spleen | Normal cell | Natural killer cell | CL_0000623 | Yipf1 | Single-cell sequencing | 24531970 | 1 |
| Mouse | Spleen | Normal cell | Monocyte | CL_0000576 | Ly6c1 | Experiment | 22213571 | 1 |
| Mouse | Spleen | Normal cell | Monocyte | CL_0000576 | Ly6g | Experiment | 22213571 | 1 |
| Mouse | Spleen | Normal cell | Monocyte | CL_0000576 | Itgam | Experiment | 26627738 | 1 |
| Mouse | Spleen | Normal cell | Monocyte | CL_0000576 | Cd19 | Experiment | 26627738 | 1 |
| Mouse | Spleen | Normal cell | Monocyte | CL_0000576 | Cd3d | Experiment | 26627738 | 1 |
| Mouse | Spleen | Normal cell | Monocyte | CL_0000576 | Cd3e | Experiment | 26627738 | 1 |
| Mouse | Spleen | Normal cell | Monocyte | CL_0000576 | Cd3g | Experiment | 26627738 | 1 |
| Mouse | Spleen | Normal cell | Monocyte | CL_0000576 | Ly6g | Experiment | 26627738 | 1 |
| Mouse | Spleen | Normal cell | Monocyte | CL_0000576 | Klrb1c | Experiment | 26627738 | 1 |
| Mouse | Spleen | Normal cell | Monocyte | CL_0000576 | Ccr2 | Company | Company | 1 |
| Mouse | Spleen | Normal cell | Monocyte | CL_0000576 | Csf1r | Company | Company | 1 |
| Mouse | Spleen | Normal cell | Monocyte | CL_0000576 | Itgam | Company | Company | 1 |
| Mouse | Spleen | Normal cell | Monocyte | CL_0000576 | Ly6c1 | Company | Company | 1 |
| Mouse | Spleen | Normal cell | Monocyte | CL_0000576 | Pithd1 | Single-cell sequencing | 24531970 | 1 |
| Mouse | Spleen | Normal cell | Monocyte | CL_0000576 | Ppp1r18 | Single-cell sequencing | 24531970 | 1 |
| Mouse | Spleen | Normal cell | Monocyte | CL_0000576 | 2310061I04Rik | Single-cell sequencing | 24531970 | 1 |
| Mouse | Spleen | Normal cell | Monocyte | CL_0000576 | Abcd1 | Single-cell sequencing | 24531970 | 1 |
| Mouse | Spleen | Normal cell | Monocyte | CL_0000576 | Abr | Single-cell sequencing | 24531970 | 1 |
| Mouse | Spleen | Normal cell | Monocyte | CL_0000576 | Acap2 | Single-cell sequencing | 24531970 | 1 |
| Mouse | Spleen | Normal cell | Monocyte | CL_0000576 | Adcy7 | Single-cell sequencing | 24531970 | 1 |
| Mouse | Spleen | Normal cell | Monocyte | CL_0000576 | Add1 | Single-cell sequencing | 24531970 | 1 |
| Mouse | Spleen | Normal cell | Monocyte | CL_0000576 | Add3 | Single-cell sequencing | 24531970 | 1 |
| Mouse | Spleen | Normal cell | Monocyte | CL_0000576 | Adipor1 | Single-cell sequencing | 24531970 | 1 |
| Mouse | Spleen | Normal cell | Monocyte | CL_0000576 | AI467606 | Single-cell sequencing | 24531970 | 1 |
| Mouse | Spleen | Normal cell | Monocyte | CL_0000576 | Alkbh7 | Single-cell sequencing | 24531970 | 1 |
| Mouse | Spleen | Normal cell | Monocyte | CL_0000576 | Ap1m1 | Single-cell sequencing | 24531970 | 1 |
| Mouse | Spleen | Normal cell | Monocyte | CL_0000576 | App | Single-cell sequencing | 24531970 | 1 |
| Mouse | Spleen | Normal cell | Monocyte | CL_0000576 | Arhgap1 | Single-cell sequencing | 24531970 | 1 |
| Mouse | Spleen | Normal cell | Monocyte | CL_0000576 | Arhgdib | Single-cell sequencing | 24531970 | 1 |
| Mouse | Spleen | Normal cell | Monocyte | CL_0000576 | Arhgef18 | Single-cell sequencing | 24531970 | 1 |
| Mouse | Spleen | Normal cell | Monocyte | CL_0000576 | Arpp19 | Single-cell sequencing | 24531970 | 1 |
| Mouse | Spleen | Normal cell | Monocyte | CL_0000576 | Aurkaip1 | Single-cell sequencing | 24531970 | 1 |
| Mouse | Spleen | Normal cell | Monocyte | CL_0000576 | B9d2 | Single-cell sequencing | 24531970 | 1 |
| Mouse | Spleen | Normal cell | Monocyte | CL_0000576 | Bin1 | Single-cell sequencing | 24531970 | 1 |
| Mouse | Spleen | Normal cell | Monocyte | CL_0000576 | Brd4 | Single-cell sequencing | 24531970 | 1 |
| Mouse | Spleen | Normal cell | Monocyte | CL_0000576 | Bri3 | Single-cell sequencing | 24531970 | 1 |
| Mouse | Spleen | Normal cell | Monocyte | CL_0000576 | Calm2 | Single-cell sequencing | 24531970 | 1 |
| Mouse | Spleen | Normal cell | Monocyte | CL_0000576 | Ccnd1 | Single-cell sequencing | 24531970 | 1 |
| Mouse | Spleen | Normal cell | Monocyte | CL_0000576 | Cd180 | Single-cell sequencing | 24531970 | 1 |
| Mouse | Spleen | Normal cell | Monocyte | CL_0000576 | Cd7 | Single-cell sequencing | 24531970 | 1 |
| Mouse | Spleen | Normal cell | Monocyte | CL_0000576 | Cdc42se2 | Single-cell sequencing | 24531970 | 1 |
| Mouse | Spleen | Normal cell | Monocyte | CL_0000576 | Cdk5rap3 | Single-cell sequencing | 24531970 | 1 |
| Mouse | Spleen | Normal cell | Monocyte | CL_0000576 | Cfp | Single-cell sequencing | 24531970 | 1 |
| Mouse | Spleen | Normal cell | Monocyte | CL_0000576 | Chka | Single-cell sequencing | 24531970 | 1 |
| Mouse | Spleen | Normal cell | Monocyte | CL_0000576 | Chst12 | Single-cell sequencing | 24531970 | 1 |
| Mouse | Spleen | Normal cell | Monocyte | CL_0000576 | Clic1 | Single-cell sequencing | 24531970 | 1 |
| Mouse | Spleen | Normal cell | Monocyte | CL_0000576 | Clta | Single-cell sequencing | 24531970 | 1 |
| Mouse | Spleen | Normal cell | Monocyte | CL_0000576 | Commd7 | Single-cell sequencing | 24531970 | 1 |
| Mouse | Spleen | Normal cell | Monocyte | CL_0000576 | Cox8a | Single-cell sequencing | 24531970 | 1 |
| Mouse | Spleen | Normal cell | Monocyte | CL_0000576 | Crip1 | Single-cell sequencing | 24531970 | 1 |
| Mouse | Spleen | Normal cell | Monocyte | CL_0000576 | Csf1r | Single-cell sequencing | 24531970 | 1 |
| Mouse | Spleen | Normal cell | Monocyte | CL_0000576 | Csk | Single-cell sequencing | 24531970 | 1 |
| Mouse | Spleen | Normal cell | Monocyte | CL_0000576 | Csnk1g2 | Single-cell sequencing | 24531970 | 1 |
| Mouse | Spleen | Normal cell | Monocyte | CL_0000576 | Cst3 | Single-cell sequencing | 24531970 | 1 |
| Mouse | Spleen | Normal cell | Monocyte | CL_0000576 | Ctnnb1 | Single-cell sequencing | 24531970 | 1 |
| Mouse | Spleen | Normal cell | Monocyte | CL_0000576 | Cux1 | Single-cell sequencing | 24531970 | 1 |
| Mouse | Spleen | Normal cell | Monocyte | CL_0000576 | Cxcr3 | Single-cell sequencing | 24531970 | 1 |
| Mouse | Spleen | Normal cell | Monocyte | CL_0000576 | Cyp27a1 | Single-cell sequencing | 24531970 | 1 |
| Mouse | Spleen | Normal cell | Monocyte | CL_0000576 | Dcaf15 | Single-cell sequencing | 24531970 | 1 |
| Mouse | Spleen | Normal cell | Monocyte | CL_0000576 | Dctpp1 | Single-cell sequencing | 24531970 | 1 |
| Mouse | Spleen | Normal cell | Monocyte | CL_0000576 | Dcxr | Single-cell sequencing | 24531970 | 1 |
| Mouse | Spleen | Normal cell | Monocyte | CL_0000576 | Ddb1 | Single-cell sequencing | 24531970 | 1 |
| Mouse | Spleen | Normal cell | Monocyte | CL_0000576 | Ddx42 | Single-cell sequencing | 24531970 | 1 |
| Mouse | Spleen | Normal cell | Monocyte | CL_0000576 | Def6 | Single-cell sequencing | 24531970 | 1 |
| Mouse | Spleen | Normal cell | Monocyte | CL_0000576 | Dhx57 | Single-cell sequencing | 24531970 | 1 |
| Mouse | Spleen | Normal cell | Monocyte | CL_0000576 | Dscr3 | Single-cell sequencing | 24531970 | 1 |
| Mouse | Spleen | Normal cell | Monocyte | CL_0000576 | Emp3 | Single-cell sequencing | 24531970 | 1 |
| Mouse | Spleen | Normal cell | Monocyte | CL_0000576 | Etfb | Single-cell sequencing | 24531970 | 1 |
| Mouse | Spleen | Normal cell | Monocyte | CL_0000576 | Fam168b | Single-cell sequencing | 24531970 | 1 |
| Mouse | Spleen | Normal cell | Monocyte | CL_0000576 | Fkbp15 | Single-cell sequencing | 24531970 | 1 |
| Mouse | Spleen | Normal cell | Monocyte | CL_0000576 | Foxo1 | Single-cell sequencing | 24531970 | 1 |
| Mouse | Spleen | Normal cell | Monocyte | CL_0000576 | Fxyd5 | Single-cell sequencing | 24531970 | 1 |
| Mouse | Spleen | Normal cell | Monocyte | CL_0000576 | Gimap6 | Single-cell sequencing | 24531970 | 1 |
| Mouse | Spleen | Normal cell | Monocyte | CL_0000576 | Gpi1 | Single-cell sequencing | 24531970 | 1 |
| Mouse | Spleen | Normal cell | Monocyte | CL_0000576 | Grap | Single-cell sequencing | 24531970 | 1 |
| Mouse | Spleen | Normal cell | Monocyte | CL_0000576 | Gtf3a | Single-cell sequencing | 24531970 | 1 |
| Mouse | Spleen | Normal cell | Monocyte | CL_0000576 | H2-Ke6 | Single-cell sequencing | 24531970 | 1 |
| Mouse | Spleen | Normal cell | Monocyte | CL_0000576 | Hadhb | Single-cell sequencing | 24531970 | 1 |
| Mouse | Spleen | Normal cell | Monocyte | CL_0000576 | Hagh | Single-cell sequencing | 24531970 | 1 |
| Mouse | Spleen | Normal cell | Monocyte | CL_0000576 | Hdac5 | Single-cell sequencing | 24531970 | 1 |
| Mouse | Spleen | Normal cell | Monocyte | CL_0000576 | Herpud1 | Single-cell sequencing | 24531970 | 1 |
| Mouse | Spleen | Normal cell | Monocyte | CL_0000576 | Hip1r | Single-cell sequencing | 24531970 | 1 |
| Mouse | Spleen | Normal cell | Monocyte | CL_0000576 | Hmgcl | Single-cell sequencing | 24531970 | 1 |
| Mouse | Spleen | Normal cell | Monocyte | CL_0000576 | Hnrnpl | Single-cell sequencing | 24531970 | 1 |
| Mouse | Spleen | Normal cell | Monocyte | CL_0000576 | Hspa1b | Single-cell sequencing | 24531970 | 1 |
| Mouse | Spleen | Normal cell | Monocyte | CL_0000576 | Ifnar2 | Single-cell sequencing | 24531970 | 1 |
| Mouse | Spleen | Normal cell | Monocyte | CL_0000576 | Igsf8 | Single-cell sequencing | 24531970 | 1 |
| Mouse | Spleen | Normal cell | Monocyte | CL_0000576 | Il17ra | Single-cell sequencing | 24531970 | 1 |
| Mouse | Spleen | Normal cell | Monocyte | CL_0000576 | Itgb7 | Single-cell sequencing | 24531970 | 1 |
| Mouse | Spleen | Normal cell | Monocyte | CL_0000576 | Kcnn4 | Single-cell sequencing | 24531970 | 1 |
| Mouse | Spleen | Normal cell | Monocyte | CL_0000576 | Klf13 | Single-cell sequencing | 24531970 | 1 |
| Mouse | Spleen | Normal cell | Monocyte | CL_0000576 | Klf2 | Single-cell sequencing | 24531970 | 1 |
| Mouse | Spleen | Normal cell | Monocyte | CL_0000576 | Klf3 | Single-cell sequencing | 24531970 | 1 |
| Mouse | Spleen | Normal cell | Monocyte | CL_0000576 | Ldb1 | Single-cell sequencing | 24531970 | 1 |
| Mouse | Spleen | Normal cell | Monocyte | CL_0000576 | Lgals3 | Single-cell sequencing | 24531970 | 1 |
| Mouse | Spleen | Normal cell | Monocyte | CL_0000576 | Lime1 | Single-cell sequencing | 24531970 | 1 |
| Mouse | Spleen | Normal cell | Monocyte | CL_0000576 | Lrwd1 | Single-cell sequencing | 24531970 | 1 |
| Mouse | Spleen | Normal cell | Monocyte | CL_0000576 | Madd | Single-cell sequencing | 24531970 | 1 |
| Mouse | Spleen | Normal cell | Monocyte | CL_0000576 | Map3k3 | Single-cell sequencing | 24531970 | 1 |
| Mouse | Spleen | Normal cell | Monocyte | CL_0000576 | Map4k1 | Single-cell sequencing | 24531970 | 1 |
| Mouse | Spleen | Normal cell | Monocyte | CL_0000576 | Mbtps1 | Single-cell sequencing | 24531970 | 1 |
| Mouse | Spleen | Normal cell | Monocyte | CL_0000576 | Mrpl18 | Single-cell sequencing | 24531970 | 1 |
| Mouse | Spleen | Normal cell | Monocyte | CL_0000576 | Mrpl40 | Single-cell sequencing | 24531970 | 1 |
| Mouse | Spleen | Normal cell | Monocyte | CL_0000576 | Myh9 | Single-cell sequencing | 24531970 | 1 |
| Mouse | Spleen | Normal cell | Monocyte | CL_0000576 | Naa10 | Single-cell sequencing | 24531970 | 1 |
| Mouse | Spleen | Normal cell | Monocyte | CL_0000576 | Nbeal2 | Single-cell sequencing | 24531970 | 1 |
| Mouse | Spleen | Normal cell | Monocyte | CL_0000576 | Ndufb5 | Single-cell sequencing | 24531970 | 1 |
| Mouse | Spleen | Normal cell | Monocyte | CL_0000576 | Nipbl | Single-cell sequencing | 24531970 | 1 |
| Mouse | Spleen | Normal cell | Monocyte | CL_0000576 | Npc1 | Single-cell sequencing | 24531970 | 1 |
| Mouse | Spleen | Normal cell | Monocyte | CL_0000576 | Nrm | Single-cell sequencing | 24531970 | 1 |
| Mouse | Spleen | Normal cell | Monocyte | CL_0000576 | Nub1 | Single-cell sequencing | 24531970 | 1 |
| Mouse | Spleen | Normal cell | Monocyte | CL_0000576 | Nudt16l1 | Single-cell sequencing | 24531970 | 1 |
| Mouse | Spleen | Normal cell | Monocyte | CL_0000576 | Phax | Single-cell sequencing | 24531970 | 1 |
| Mouse | Spleen | Normal cell | Monocyte | CL_0000576 | Pnn | Single-cell sequencing | 24531970 | 1 |
| Mouse | Spleen | Normal cell | Monocyte | CL_0000576 | Pnpla7 | Single-cell sequencing | 24531970 | 1 |
| Mouse | Spleen | Normal cell | Monocyte | CL_0000576 | Ppp1ca | Single-cell sequencing | 24531970 | 1 |
| Mouse | Spleen | Normal cell | Monocyte | CL_0000576 | Prkacb | Single-cell sequencing | 24531970 | 1 |
| Mouse | Spleen | Normal cell | Monocyte | CL_0000576 | Prkd2 | Single-cell sequencing | 24531970 | 1 |
| Mouse | Spleen | Normal cell | Monocyte | CL_0000576 | Psmd4 | Single-cell sequencing | 24531970 | 1 |
| Mouse | Spleen | Normal cell | Monocyte | CL_0000576 | Ptpn6 | Single-cell sequencing | 24531970 | 1 |
| Mouse | Spleen | Normal cell | Monocyte | CL_0000576 | Rasal3 | Single-cell sequencing | 24531970 | 1 |
| Mouse | Spleen | Normal cell | Monocyte | CL_0000576 | Rasgrp2 | Single-cell sequencing | 24531970 | 1 |
| Mouse | Spleen | Normal cell | Monocyte | CL_0000576 | Rbm38 | Single-cell sequencing | 24531970 | 1 |
| Mouse | Spleen | Normal cell | Monocyte | CL_0000576 | Reep4 | Single-cell sequencing | 24531970 | 1 |
| Mouse | Spleen | Normal cell | Monocyte | CL_0000576 | Rheb | Single-cell sequencing | 24531970 | 1 |
| Mouse | Spleen | Normal cell | Monocyte | CL_0000576 | S1pr4 | Single-cell sequencing | 24531970 | 1 |
| Mouse | Spleen | Normal cell | Monocyte | CL_0000576 | Sgk1 | Single-cell sequencing | 24531970 | 1 |
| Mouse | Spleen | Normal cell | Monocyte | CL_0000576 | Slc50a1 | Single-cell sequencing | 24531970 | 1 |
| Mouse | Spleen | Normal cell | Monocyte | CL_0000576 | Slpi | Single-cell sequencing | 24531970 | 1 |
| Mouse | Spleen | Normal cell | Monocyte | CL_0000576 | Smc4 | Single-cell sequencing | 24531970 | 1 |
| Mouse | Spleen | Normal cell | Monocyte | CL_0000576 | Snx3 | Single-cell sequencing | 24531970 | 1 |
| Mouse | Spleen | Normal cell | Monocyte | CL_0000576 | Sra1 | Single-cell sequencing | 24531970 | 1 |
| Mouse | Spleen | Normal cell | Monocyte | CL_0000576 | Srsf9 | Single-cell sequencing | 24531970 | 1 |
| Mouse | Spleen | Normal cell | Monocyte | CL_0000576 | Stk38 | Single-cell sequencing | 24531970 | 1 |
| Mouse | Spleen | Normal cell | Monocyte | CL_0000576 | Sumf1 | Single-cell sequencing | 24531970 | 1 |
| Mouse | Spleen | Normal cell | Monocyte | CL_0000576 | Tbc1d10c | Single-cell sequencing | 24531970 | 1 |
| Mouse | Spleen | Normal cell | Monocyte | CL_0000576 | Tbc1d17 | Single-cell sequencing | 24531970 | 1 |
| Mouse | Spleen | Normal cell | Monocyte | CL_0000576 | Tcp11l2 | Single-cell sequencing | 24531970 | 1 |
| Mouse | Spleen | Normal cell | Monocyte | CL_0000576 | Tmed3 | Single-cell sequencing | 24531970 | 1 |
| Mouse | Spleen | Normal cell | Monocyte | CL_0000576 | Tmem14c | Single-cell sequencing | 24531970 | 1 |
| Mouse | Spleen | Normal cell | Monocyte | CL_0000576 | Tmem160 | Single-cell sequencing | 24531970 | 1 |
| Mouse | Spleen | Normal cell | Monocyte | CL_0000576 | Tmem208 | Single-cell sequencing | 24531970 | 1 |
| Mouse | Spleen | Normal cell | Monocyte | CL_0000576 | Tmem59 | Single-cell sequencing | 24531970 | 1 |
| Mouse | Spleen | Normal cell | Monocyte | CL_0000576 | Tpi1 | Single-cell sequencing | 24531970 | 1 |
| Mouse | Spleen | Normal cell | Monocyte | CL_0000576 | Tpm1 | Single-cell sequencing | 24531970 | 1 |
| Mouse | Spleen | Normal cell | Monocyte | CL_0000576 | Tprgl | Single-cell sequencing | 24531970 | 1 |
| Mouse | Spleen | Normal cell | Monocyte | CL_0000576 | Trim8 | Single-cell sequencing | 24531970 | 1 |
| Mouse | Spleen | Normal cell | Monocyte | CL_0000576 | Tsnax | Single-cell sequencing | 24531970 | 1 |
| Mouse | Spleen | Normal cell | Monocyte | CL_0000576 | Tspan32 | Single-cell sequencing | 24531970 | 1 |
| Mouse | Spleen | Normal cell | Monocyte | CL_0000576 | Vim | Single-cell sequencing | 24531970 | 1 |
| Mouse | Spleen | Normal cell | Monocyte | CL_0000576 | Vps16 | Single-cell sequencing | 24531970 | 1 |
| Mouse | Spleen | Normal cell | Monocyte | CL_0000576 | Wbp2 | Single-cell sequencing | 24531970 | 1 |
| Mouse | Spleen | Normal cell | Monocyte | CL_0000576 | Wdfy4 | Single-cell sequencing | 24531970 | 1 |
| Mouse | Spleen | Normal cell | T helper 2 cell | CL_0000546 | Il1rl1 | Experiment | 11207266 | 1 |
| Mouse | Spleen | Normal cell | Lymphocyte | CL_0000542 | Cd19 | Experiment | 26627738 | 1 |
| Mouse | Spleen | Normal cell | Lymphocyte | CL_0000542 | Cd3d | Experiment | 26627738 | 1 |
| Mouse | Spleen | Normal cell | Lymphocyte | CL_0000542 | Cd3e | Experiment | 26627738 | 1 |
| Mouse | Spleen | Normal cell | Lymphocyte | CL_0000542 | Cd3g | Experiment | 26627738 | 1 |
| Mouse | Spleen | Normal cell | Lymphocyte | CL_0000542 | Klrb1c | Experiment | 26627738 | 1 |
| Mouse | Spleen | Normal cell | CD4^+^ T helper cell | CL_0000492 | Cd3d | Company | Company | 1 |
| Mouse | Spleen | Normal cell | CD4^+^ T helper cell | CL_0000492 | Cd3e | Company | Company | 1 |
| Mouse | Spleen | Normal cell | CD4^+^ T helper cell | CL_0000492 | Cd3g | Company | Company | 1 |
| Mouse | Spleen | Normal cell | CD4^+^ T helper cell | CL_0000492 | Cd4 | Company | Company | 1 |
| Mouse | Spleen | Normal cell | CD4^+^ T helper cell | CL_0000492 | Ptprc | Company | Company | 1 |
| Mouse | Spleen | Normal cell | Dendritic cell | CL_0000451 | Itgax | Experiment | 27832135 | 1 |
| Mouse | Spleen | Normal cell | Dendritic cell | CL_0000451 | Cd74 | Experiment | 27832135 | 1 |
| Mouse | Spleen | Normal cell | Dendritic cell | CL_0000451 | Cd83 | Experiment | 27832135 | 1 |
| Mouse | Spleen | Normal cell | Dendritic cell | CL_0000451 | Cd86 | Experiment | 27832135 | 1 |
| Mouse | Spleen | Normal cell | Dendritic cell | CL_0000451 | Itgam | Experiment | 26627738 | 1 |
| Mouse | Spleen | Normal cell | Dendritic cell | CL_0000451 | Cd19 | Experiment | 26627738 | 1 |
| Mouse | Spleen | Normal cell | Dendritic cell | CL_0000451 | Cd3d | Experiment | 26627738 | 1 |
| Mouse | Spleen | Normal cell | Dendritic cell | CL_0000451 | Cd3e | Experiment | 26627738 | 1 |
| Mouse | Spleen | Normal cell | Dendritic cell | CL_0000451 | Cd3g | Experiment | 26627738 | 1 |
| Mouse | Spleen | Normal cell | Dendritic cell | CL_0000451 | Ly6g | Experiment | 26627738 | 1 |
| Mouse | Spleen | Normal cell | Dendritic cell | CL_0000451 | Klrb1c | Experiment | 26627738 | 1 |
| Mouse | Spleen | Normal cell | Dendritic cell | CL_0000451 | Itgax | Company | Company | 1 |
| Mouse | Spleen | Normal cell | Dendritic cell | CL_0000451 | MHC class II | Company | Company | 1 |
| Mouse | Spleen | Normal cell | Dendritic cell | CL_0000451 | Xcr1 | Company | Company | 1 |
| Mouse | Spleen | Normal cell | B cell | CL_0000236 | Cd19 | Experiment | 29373896 | 1 |
| Mouse | Spleen | Normal cell | B cell | CL_0000236 | Cd79a | Experiment | 29373896 | 1 |
| Mouse | Spleen | Normal cell | B cell | CL_0000236 | Fasn | Experiment | 24052571 | 1 |
| Mouse | Spleen | Normal cell | B cell | CL_0000236 | Cd19 | Company | Company | 1 |
| Mouse | Spleen | Normal cell | B cell | CL_0000236 | Ptprc | Company | Company | 1 |
| Mouse | Spleen | Normal cell | B cell | CL_0000236 | Cd69 | Single-cell sequencing | 24531970 | 1 |
| Mouse | Spleen | Normal cell | B cell | CL_0000236 | Cxcl10 | Single-cell sequencing | 24531970 | 1 |
| Mouse | Spleen | Normal cell | B cell | CL_0000236 | Ifi47 | Single-cell sequencing | 24531970 | 1 |
| Mouse | Spleen | Normal cell | B cell | CL_0000236 | Ifit1 | Single-cell sequencing | 24531970 | 1 |
| Mouse | Spleen | Normal cell | B cell | CL_0000236 | Ifit2 | Single-cell sequencing | 24531970 | 1 |
| Mouse | Spleen | Normal cell | B cell | CL_0000236 | Ifit3 | Single-cell sequencing | 24531970 | 1 |
| Mouse | Spleen | Normal cell | B cell | CL_0000236 | Igtp | Single-cell sequencing | 24531970 | 1 |
| Mouse | Spleen | Normal cell | B cell | CL_0000236 | Irf7 | Single-cell sequencing | 24531970 | 1 |
| Mouse | Spleen | Normal cell | B cell | CL_0000236 | Irgm1 | Single-cell sequencing | 24531970 | 1 |
| Mouse | Spleen | Normal cell | B cell | CL_0000236 | Isg15 | Single-cell sequencing | 24531970 | 1 |
| Mouse | Spleen | Normal cell | B cell | CL_0000236 | Isg20 | Single-cell sequencing | 24531970 | 1 |
| Mouse | Spleen | Normal cell | B cell | CL_0000236 | Mx1 | Single-cell sequencing | 24531970 | 1 |
| Mouse | Spleen | Normal cell | B cell | CL_0000236 | Oasl1 | Single-cell sequencing | 24531970 | 1 |
| Mouse | Spleen | Normal cell | B cell | CL_0000236 | Parp14 | Single-cell sequencing | 24531970 | 1 |
| Mouse | Spleen | Normal cell | B cell | CL_0000236 | Rgs1 | Single-cell sequencing | 24531970 | 1 |
| Mouse | Spleen | Normal cell | B cell | CL_0000236 | Rsad2 | Single-cell sequencing | 24531970 | 1 |
| Mouse | Spleen | Normal cell | B cell | CL_0000236 | Usp18 | Single-cell sequencing | 24531970 | 1 |
| Mouse | Spleen | Normal cell | Macrophage | CL_0000235 | Ly6c1 | Experiment | 22213571 | 1 |
| Mouse | Spleen | Normal cell | Macrophage | CL_0000235 | Ly6g | Experiment | 22213571 | 1 |
| Mouse | Spleen | Normal cell | Macrophage | CL_0000235 | Cd68 | Experiment | 17187680 | 1 |
| Mouse | Spleen | Normal cell | Macrophage | CL_0000235 | Adgre1 | Experiment | 16364031 | 1 |
| Mouse | Spleen | Normal cell | Macrophage | CL_0000235 | Adgre1 | Experiment | 9498754 | 1 |
| Mouse | Spleen | Normal cell | Macrophage | CL_0000235 | Itgam | Experiment | 9498754 | 1 |
| Mouse | Spleen | Normal cell | Macrophage | CL_0000235 | Adgre1 | Experiment | 7590892 | 1 |
| Mouse | Spleen | Normal cell | Macrophage | CL_0000235 | Itgam | Experiment | 7590892 | 1 |
| Mouse | Spleen | Normal cell | Macrophage | CL_0000235 | Cd19 | Experiment | 26627738 | 1 |
| Mouse | Spleen | Normal cell | Macrophage | CL_0000235 | Cd3d | Experiment | 26627738 | 1 |
| Mouse | Spleen | Normal cell | Macrophage | CL_0000235 | Cd3e | Experiment | 26627738 | 1 |
| Mouse | Spleen | Normal cell | Macrophage | CL_0000235 | Cd3g | Experiment | 26627738 | 1 |
| Mouse | Spleen | Normal cell | Macrophage | CL_0000235 | Adgre1 | Experiment | 26627738 | 1 |
| Mouse | Spleen | Normal cell | Macrophage | CL_0000235 | Ly6g | Experiment | 26627738 | 1 |
| Mouse | Spleen | Normal cell | Macrophage | CL_0000235 | Klrb1c | Experiment | 26627738 | 1 |
| Mouse | Spleen | Normal cell | Macrophage | CL_0000235 | Adgre1 | Company | Company | 1 |
| Mouse | Spleen | Normal cell | Macrophage | CL_0000235 | Adamts10 | Single-cell sequencing | 24531970 | 1 |
| Mouse | Spleen | Normal cell | Macrophage | CL_0000235 | Alkbh6 | Single-cell sequencing | 24531970 | 1 |
| Mouse | Spleen | Normal cell | Macrophage | CL_0000235 | Anapc5 | Single-cell sequencing | 24531970 | 1 |
| Mouse | Spleen | Normal cell | Macrophage | CL_0000235 | Anp32b | Single-cell sequencing | 24531970 | 1 |
| Mouse | Spleen | Normal cell | Macrophage | CL_0000235 | Ap1s1 | Single-cell sequencing | 24531970 | 1 |
| Mouse | Spleen | Normal cell | Macrophage | CL_0000235 | Apex1 | Single-cell sequencing | 24531970 | 1 |
| Mouse | Spleen | Normal cell | Macrophage | CL_0000235 | Arhgef11 | Single-cell sequencing | 24531970 | 1 |
| Mouse | Spleen | Normal cell | Macrophage | CL_0000235 | Asna1 | Single-cell sequencing | 24531970 | 1 |
| Mouse | Spleen | Normal cell | Macrophage | CL_0000235 | Atrx | Single-cell sequencing | 24531970 | 1 |
| Mouse | Spleen | Normal cell | Macrophage | CL_0000235 | B3gat3 | Single-cell sequencing | 24531970 | 1 |
| Mouse | Spleen | Normal cell | Macrophage | CL_0000235 | Banf1 | Single-cell sequencing | 24531970 | 1 |
| Mouse | Spleen | Normal cell | Macrophage | CL_0000235 | Bbip1 | Single-cell sequencing | 24531970 | 1 |
| Mouse | Spleen | Normal cell | Macrophage | CL_0000235 | Blk | Single-cell sequencing | 24531970 | 1 |
| Mouse | Spleen | Normal cell | Macrophage | CL_0000235 | Brd1 | Single-cell sequencing | 24531970 | 1 |
| Mouse | Spleen | Normal cell | Macrophage | CL_0000235 | Calcoco1 | Single-cell sequencing | 24531970 | 1 |
| Mouse | Spleen | Normal cell | Macrophage | CL_0000235 | Camta2 | Single-cell sequencing | 24531970 | 1 |
| Mouse | Spleen | Normal cell | Macrophage | CL_0000235 | Card11 | Single-cell sequencing | 24531970 | 1 |
| Mouse | Spleen | Normal cell | Macrophage | CL_0000235 | Ccdc88c | Single-cell sequencing | 24531970 | 1 |
| Mouse | Spleen | Normal cell | Macrophage | CL_0000235 | Cct4 | Single-cell sequencing | 24531970 | 1 |
| Mouse | Spleen | Normal cell | Macrophage | CL_0000235 | Cd3d | Single-cell sequencing | 24531970 | 1 |
| Mouse | Spleen | Normal cell | Macrophage | CL_0000235 | Chmp1b | Single-cell sequencing | 24531970 | 1 |
| Mouse | Spleen | Normal cell | Macrophage | CL_0000235 | Cirbp | Single-cell sequencing | 24531970 | 1 |
| Mouse | Spleen | Normal cell | Macrophage | CL_0000235 | Ciz1 | Single-cell sequencing | 24531970 | 1 |
| Mouse | Spleen | Normal cell | Macrophage | CL_0000235 | Clint1 | Single-cell sequencing | 24531970 | 1 |
| Mouse | Spleen | Normal cell | Macrophage | CL_0000235 | Clpp | Single-cell sequencing | 24531970 | 1 |
| Mouse | Spleen | Normal cell | Macrophage | CL_0000235 | Cmtm7 | Single-cell sequencing | 24531970 | 1 |
| Mouse | Spleen | Normal cell | Macrophage | CL_0000235 | Cndp2 | Single-cell sequencing | 24531970 | 1 |
| Mouse | Spleen | Normal cell | Macrophage | CL_0000235 | Coro1c | Single-cell sequencing | 24531970 | 1 |
| Mouse | Spleen | Normal cell | Macrophage | CL_0000235 | Crebbp | Single-cell sequencing | 24531970 | 1 |
| Mouse | Spleen | Normal cell | Macrophage | CL_0000235 | Creld2 | Single-cell sequencing | 24531970 | 1 |
| Mouse | Spleen | Normal cell | Macrophage | CL_0000235 | Ctnnbl1 | Single-cell sequencing | 24531970 | 1 |
| Mouse | Spleen | Normal cell | Macrophage | CL_0000235 | Cxxc5 | Single-cell sequencing | 24531970 | 1 |
| Mouse | Spleen | Normal cell | Macrophage | CL_0000235 | Dcps | Single-cell sequencing | 24531970 | 1 |
| Mouse | Spleen | Normal cell | Macrophage | CL_0000235 | Dctn2 | Single-cell sequencing | 24531970 | 1 |
| Mouse | Spleen | Normal cell | Macrophage | CL_0000235 | Dtnbp1 | Single-cell sequencing | 24531970 | 1 |
| Mouse | Spleen | Normal cell | Macrophage | CL_0000235 | Dtx2 | Single-cell sequencing | 24531970 | 1 |
| Mouse | Spleen | Normal cell | Macrophage | CL_0000235 | Ensa | Single-cell sequencing | 24531970 | 1 |
| Mouse | Spleen | Normal cell | Macrophage | CL_0000235 | Fam129c | Single-cell sequencing | 24531970 | 1 |
| Mouse | Spleen | Normal cell | Macrophage | CL_0000235 | Fam160b2 | Single-cell sequencing | 24531970 | 1 |
| Mouse | Spleen | Normal cell | Macrophage | CL_0000235 | Fam98c | Single-cell sequencing | 24531970 | 1 |
| Mouse | Spleen | Normal cell | Macrophage | CL_0000235 | Fcgr2b | Single-cell sequencing | 24531970 | 1 |
| Mouse | Spleen | Normal cell | Macrophage | CL_0000235 | Fcrla | Single-cell sequencing | 24531970 | 1 |
| Mouse | Spleen | Normal cell | Macrophage | CL_0000235 | Foxred2 | Single-cell sequencing | 24531970 | 1 |
| Mouse | Spleen | Normal cell | Macrophage | CL_0000235 | Gale | Single-cell sequencing | 24531970 | 1 |
| Mouse | Spleen | Normal cell | Macrophage | CL_0000235 | Gnb2 | Single-cell sequencing | 24531970 | 1 |
| Mouse | Spleen | Normal cell | Macrophage | CL_0000235 | Golga4 | Single-cell sequencing | 24531970 | 1 |
| Mouse | Spleen | Normal cell | Macrophage | CL_0000235 | Grhpr | Single-cell sequencing | 24531970 | 1 |
| Mouse | Spleen | Normal cell | Macrophage | CL_0000235 | Hbp1 | Single-cell sequencing | 24531970 | 1 |
| Mouse | Spleen | Normal cell | Macrophage | CL_0000235 | Hipk3 | Single-cell sequencing | 24531970 | 1 |
| Mouse | Spleen | Normal cell | Macrophage | CL_0000235 | Hsd3b7 | Single-cell sequencing | 24531970 | 1 |
| Mouse | Spleen | Normal cell | Macrophage | CL_0000235 | Hsdl1 | Single-cell sequencing | 24531970 | 1 |
| Mouse | Spleen | Normal cell | Macrophage | CL_0000235 | Iqgap2 | Single-cell sequencing | 24531970 | 1 |
| Mouse | Spleen | Normal cell | Macrophage | CL_0000235 | Itpr3 | Single-cell sequencing | 24531970 | 1 |
| Mouse | Spleen | Normal cell | Macrophage | CL_0000235 | Klf4 | Single-cell sequencing | 24531970 | 1 |
| Mouse | Spleen | Normal cell | Macrophage | CL_0000235 | Klhl6 | Single-cell sequencing | 24531970 | 1 |
| Mouse | Spleen | Normal cell | Macrophage | CL_0000235 | Lsp1 | Single-cell sequencing | 24531970 | 1 |
| Mouse | Spleen | Normal cell | Macrophage | CL_0000235 | Lxn | Single-cell sequencing | 24531970 | 1 |
| Mouse | Spleen | Normal cell | Macrophage | CL_0000235 | Lysmd2 | Single-cell sequencing | 24531970 | 1 |
| Mouse | Spleen | Normal cell | Macrophage | CL_0000235 | Med8 | Single-cell sequencing | 24531970 | 1 |
| Mouse | Spleen | Normal cell | Macrophage | CL_0000235 | Mrpl46 | Single-cell sequencing | 24531970 | 1 |
| Mouse | Spleen | Normal cell | Macrophage | CL_0000235 | Mybbp1a | Single-cell sequencing | 24531970 | 1 |
| Mouse | Spleen | Normal cell | Macrophage | CL_0000235 | Myg1 | Single-cell sequencing | 24531970 | 1 |
| Mouse | Spleen | Normal cell | Macrophage | CL_0000235 | Nap1l4 | Single-cell sequencing | 24531970 | 1 |
| Mouse | Spleen | Normal cell | Macrophage | CL_0000235 | Nckipsd | Single-cell sequencing | 24531970 | 1 |
| Mouse | Spleen | Normal cell | Macrophage | CL_0000235 | Ncoa6 | Single-cell sequencing | 24531970 | 1 |
| Mouse | Spleen | Normal cell | Macrophage | CL_0000235 | Nenf | Single-cell sequencing | 24531970 | 1 |
| Mouse | Spleen | Normal cell | Macrophage | CL_0000235 | Nhp2 | Single-cell sequencing | 24531970 | 1 |
| Mouse | Spleen | Normal cell | Macrophage | CL_0000235 | Ninj1 | Single-cell sequencing | 24531970 | 1 |
| Mouse | Spleen | Normal cell | Macrophage | CL_0000235 | Nmt1 | Single-cell sequencing | 24531970 | 1 |
| Mouse | Spleen | Normal cell | Macrophage | CL_0000235 | Nop16 | Single-cell sequencing | 24531970 | 1 |
| Mouse | Spleen | Normal cell | Macrophage | CL_0000235 | Npepl1 | Single-cell sequencing | 24531970 | 1 |
| Mouse | Spleen | Normal cell | Macrophage | CL_0000235 | Nr2c2ap | Single-cell sequencing | 24531970 | 1 |
| Mouse | Spleen | Normal cell | Macrophage | CL_0000235 | Ostf1 | Single-cell sequencing | 24531970 | 1 |
| Mouse | Spleen | Normal cell | Macrophage | CL_0000235 | Pafah1b3 | Single-cell sequencing | 24531970 | 1 |
| Mouse | Spleen | Normal cell | Macrophage | CL_0000235 | Pik3c3 | Single-cell sequencing | 24531970 | 1 |
| Mouse | Spleen | Normal cell | Macrophage | CL_0000235 | Pik3r4 | Single-cell sequencing | 24531970 | 1 |
| Mouse | Spleen | Normal cell | Macrophage | CL_0000235 | Pknox1 | Single-cell sequencing | 24531970 | 1 |
| Mouse | Spleen | Normal cell | Macrophage | CL_0000235 | Pold3 | Single-cell sequencing | 24531970 | 1 |
| Mouse | Spleen | Normal cell | Macrophage | CL_0000235 | Pon2 | Single-cell sequencing | 24531970 | 1 |
| Mouse | Spleen | Normal cell | Macrophage | CL_0000235 | Ppfia4 | Single-cell sequencing | 24531970 | 1 |
| Mouse | Spleen | Normal cell | Macrophage | CL_0000235 | Ppm1g | Single-cell sequencing | 24531970 | 1 |
| Mouse | Spleen | Normal cell | Macrophage | CL_0000235 | Pqlc3 | Single-cell sequencing | 24531970 | 1 |
| Mouse | Spleen | Normal cell | Macrophage | CL_0000235 | Prkar1a | Single-cell sequencing | 24531970 | 1 |
| Mouse | Spleen | Normal cell | Macrophage | CL_0000235 | Prmt7 | Single-cell sequencing | 24531970 | 1 |
| Mouse | Spleen | Normal cell | Macrophage | CL_0000235 | Ptprf | Single-cell sequencing | 24531970 | 1 |
| Mouse | Spleen | Normal cell | Macrophage | CL_0000235 | Pts | Single-cell sequencing | 24531970 | 1 |
| Mouse | Spleen | Normal cell | Macrophage | CL_0000235 | Rai1 | Single-cell sequencing | 24531970 | 1 |
| Mouse | Spleen | Normal cell | Macrophage | CL_0000235 | Rbm5 | Single-cell sequencing | 24531970 | 1 |
| Mouse | Spleen | Normal cell | Macrophage | CL_0000235 | Relt | Single-cell sequencing | 24531970 | 1 |
| Mouse | Spleen | Normal cell | Macrophage | CL_0000235 | Rnaseh2c | Single-cell sequencing | 24531970 | 1 |
| Mouse | Spleen | Normal cell | Macrophage | CL_0000235 | Rnpepl1 | Single-cell sequencing | 24531970 | 1 |
| Mouse | Spleen | Normal cell | Macrophage | CL_0000235 | Rpl11 | Single-cell sequencing | 24531970 | 1 |
| Mouse | Spleen | Normal cell | Macrophage | CL_0000235 | Rpl15 | Single-cell sequencing | 24531970 | 1 |
| Mouse | Spleen | Normal cell | Macrophage | CL_0000235 | Rpl28 | Single-cell sequencing | 24531970 | 1 |
| Mouse | Spleen | Normal cell | Macrophage | CL_0000235 | Rufy1 | Single-cell sequencing | 24531970 | 1 |
| Mouse | Spleen | Normal cell | Macrophage | CL_0000235 | S1pr1 | Single-cell sequencing | 24531970 | 1 |
| Mouse | Spleen | Normal cell | Macrophage | CL_0000235 | Scamp2 | Single-cell sequencing | 24531970 | 1 |
| Mouse | Spleen | Normal cell | Macrophage | CL_0000235 | Sec13 | Single-cell sequencing | 24531970 | 1 |
| Mouse | Spleen | Normal cell | Macrophage | CL_0000235 | Sh2b1 | Single-cell sequencing | 24531970 | 1 |
| Mouse | Spleen | Normal cell | Macrophage | CL_0000235 | Sh2b2 | Single-cell sequencing | 24531970 | 1 |
| Mouse | Spleen | Normal cell | Macrophage | CL_0000235 | Sh3glb2 | Single-cell sequencing | 24531970 | 1 |
| Mouse | Spleen | Normal cell | Macrophage | CL_0000235 | Slc25a28 | Single-cell sequencing | 24531970 | 1 |
| Mouse | Spleen | Normal cell | Macrophage | CL_0000235 | Smarcd1 | Single-cell sequencing | 24531970 | 1 |
| Mouse | Spleen | Normal cell | Macrophage | CL_0000235 | Snw1 | Single-cell sequencing | 24531970 | 1 |
| Mouse | Spleen | Normal cell | Macrophage | CL_0000235 | Sp3 | Single-cell sequencing | 24531970 | 1 |
| Mouse | Spleen | Normal cell | Macrophage | CL_0000235 | Spib | Single-cell sequencing | 24531970 | 1 |
| Mouse | Spleen | Normal cell | Macrophage | CL_0000235 | Srebf2 | Single-cell sequencing | 24531970 | 1 |
| Mouse | Spleen | Normal cell | Macrophage | CL_0000235 | St6gal1 | Single-cell sequencing | 24531970 | 1 |
| Mouse | Spleen | Normal cell | Macrophage | CL_0000235 | Stk4 | Single-cell sequencing | 24531970 | 1 |
| Mouse | Spleen | Normal cell | Macrophage | CL_0000235 | Tbc1d2b | Single-cell sequencing | 24531970 | 1 |
| Mouse | Spleen | Normal cell | Macrophage | CL_0000235 | Tifab | Single-cell sequencing | 24531970 | 1 |
| Mouse | Spleen | Normal cell | Macrophage | CL_0000235 | Tmem163 | Single-cell sequencing | 24531970 | 1 |
| Mouse | Spleen | Normal cell | Macrophage | CL_0000235 | Tmem221 | Single-cell sequencing | 24531970 | 1 |
| Mouse | Spleen | Normal cell | Macrophage | CL_0000235 | Tnfrsf13c | Single-cell sequencing | 24531970 | 1 |
| Mouse | Spleen | Normal cell | Macrophage | CL_0000235 | Tor2a | Single-cell sequencing | 24531970 | 1 |
| Mouse | Spleen | Normal cell | Macrophage | CL_0000235 | Tpd52 | Single-cell sequencing | 24531970 | 1 |
| Mouse | Spleen | Normal cell | Macrophage | CL_0000235 | Tppp3 | Single-cell sequencing | 24531970 | 1 |
| Mouse | Spleen | Normal cell | Macrophage | CL_0000235 | Trappc5 | Single-cell sequencing | 24531970 | 1 |
| Mouse | Spleen | Normal cell | Macrophage | CL_0000235 | Trit1 | Single-cell sequencing | 24531970 | 1 |
| Mouse | Spleen | Normal cell | Macrophage | CL_0000235 | Trmt1 | Single-cell sequencing | 24531970 | 1 |
| Mouse | Spleen | Normal cell | Macrophage | CL_0000235 | Tsc22d1 | Single-cell sequencing | 24531970 | 1 |
| Mouse | Spleen | Normal cell | Macrophage | CL_0000235 | Ttc7 | Single-cell sequencing | 24531970 | 1 |
| Mouse | Spleen | Normal cell | Macrophage | CL_0000235 | Ube2l3 | Single-cell sequencing | 24531970 | 1 |
| Mouse | Spleen | Normal cell | Polymorphonuclear neutrophil | CL_0000096 | Ly6g | Experiment | 11591793 | 1 |
| Mouse | Spleen | Normal cell | Granulocyte | CL_0000094 | Itgam | Experiment | 26627738 | 1 |
| Mouse | Spleen | Normal cell | Granulocyte | CL_0000094 | Cd19 | Experiment | 26627738 | 1 |
| Mouse | Spleen | Normal cell | Granulocyte | CL_0000094 | Cd3d | Experiment | 26627738 | 1 |
| Mouse | Spleen | Normal cell | Granulocyte | CL_0000094 | Cd3e | Experiment | 26627738 | 1 |
| Mouse | Spleen | Normal cell | Granulocyte | CL_0000094 | Cd3g | Experiment | 26627738 | 1 |
| Mouse | Spleen | Normal cell | Granulocyte | CL_0000094 | Ly6g | Experiment | 26627738 | 1 |
| Mouse | Spleen | Normal cell | Granulocyte | CL_0000094 | Klrb1c | Experiment | 26627738 | 1 |
| Mouse | Spleen | Normal cell | Granulocyte | CL_0000094 | Itgam | Company | Company | 1 |
| Mouse | Spleen | Normal cell | Granulocyte | CL_0000094 | Ccr3 | Company | Company | 1 |
| Mouse | Spleen | Normal cell | Granulocyte | CL_0000094 | Itga2 | Company | Company | 1 |
| Mouse | Spleen | Normal cell | Granulocyte | CL_0000094 | Ly6c1 | Company | Company | 1 |
| Mouse | Spleen | Normal cell | Granulocyte | CL_0000094 | Siglecf | Company | Company | 1 |
| Mouse | Spleen | Normal cell | T cell | CL_0000084 | Cd4 | Experiment | 11145690 | 1 |
| Mouse | Spleen | Normal cell | T cell | CL_0000084 | Cd8a | Experiment | 11145690 | 1 |
| Mouse | Spleen | Normal cell | T cell | CL_0000084 | Sell | Experiment | 1909300 | 1 |
| Mouse | Spleen | Normal cell | T cell | CL_0000084 | Cd3d | Experiment | 25338846 | 1 |
| Mouse | Spleen | Normal cell | T cell | CL_0000084 | Cd3e | Experiment | 25338846 | 1 |
| Mouse | Spleen | Normal cell | T cell | CL_0000084 | Cd3g | Experiment | 25338846 | 1 |
| Mouse | Spleen | Normal cell | T cell | CL_0000084 | Cd3d | Experiment | 8562871 | 1 |
| Mouse | Spleen | Normal cell | T cell | CL_0000084 | Cd3e | Experiment | 8562871 | 1 |
| Mouse | Spleen | Normal cell | T cell | CL_0000084 | Cd3g | Experiment | 8562871 | 1 |
| Mouse | Spleen | Normal cell | T cell | CL_0000084 | Cd4 | Experiment | 8562871 | 1 |
| Mouse | Spleen | Normal cell | T cell | CL_0000084 | Thy1 | Experiment | 8407056 | 1 |
| Mouse | Spleen | Normal cell | T cell | CL_0000084 | Cd3d | Experiment | 2045127 | 1 |
| Mouse | Spleen | Normal cell | T cell | CL_0000084 | Cd3e | Experiment | 2045127 | 1 |
| Mouse | Spleen | Normal cell | T cell | CL_0000084 | Cd3g | Experiment | 2045127 | 1 |
| Mouse | Spleen | Normal cell | T cell | CL_0000084 | Cd4 | Experiment | 2045127 | 1 |
| Mouse | Spleen | Normal cell | T cell | CL_0000084 | Cd8a | Experiment | 2045127 | 1 |
| Mouse | Spleen | Normal cell | T cell | CL_0000084 | Cd3d | Company | Company | 2 |
| Mouse | Spleen | Normal cell | T cell | CL_0000084 | Cd3e | Company | Company | 2 |
| Mouse | Spleen | Normal cell | T cell | CL_0000084 | Cd3g | Company | Company | 2 |
| Mouse | Spleen | Normal cell | T cell | CL_0000084 | Cd4 | Company | Company | 1 |
| Mouse | Spleen | Normal cell | T cell | CL_0000084 | Cd8a | Company | Company | 1 |
| Mouse | Spleen | Normal cell | T cell | CL_0000084 | Thy1 | Company | Company | 2 |
| Mouse | Spleen | Normal cell | T cell | CL_0000084 | Cd2 | Company | Company | 1 |
| Mouse | Spleen | Normal cell | T cell | CL_0000084 | Ptprc | Company | Company | 1 |
| Mouse | Spleen | Normal cell | T cell | CL_0000084 | Cd5 | Company | Company | 1 |
| Mouse | Spleen | Normal cell | T cell | CL_0000084 | Itgb3 | Company | Company | 1 |
| Mouse | Spleen | Normal cell | Erythroid progenitor | CL_0000038 | Tfrc | Experiment | 21847081 | 1 |
| Mouse | Spleen | Normal cell | Erythroid progenitor | CL_0000038 | Ly76 | Experiment | 21847081 | 1 |

| Table S5 The amount of cells corresponding to the samples in each cluster. | | | | | | | |  |
| --- | --- | --- | --- | --- | --- | --- | --- | --- |
| Cell type | Cell cluster | BT-Pri 1 cell count | BT-Pri 2 cell count | BT-Pri 3 cell count | BT-Pri-IL-38 1 cell count | BT-Pri-IL-38 2 cell count | BT-Pri-IL-38 3 cell count | P value |
| T cell | 2 | 902 | 347 | 383 | 266 | 503 | 449 | 0.025 |
|  | 3 | 578 | 378 | 306 | 244 | 452 | 430 |  |
|  | 10 | 391 | 176 | 206 | 199 | 235 | 267 |  |
|  | 14 | 369 | 225 | 204 | 141 | 191 | 242 |  |
|  | 15 | 259 | 189 | 320 | 157 | 132 | 196 |  |
|  | 23 | 156 | 101 | 91 | 63 | 110 | 109 |  |
|  | 29 | 21 | 11 | 11 | 6 | 27 | 29 |  |
|  | 30 | 10 | 7 | 7 | 5 | 4 | 3 |  |
| B cell | 0 | 1776 | 957 | 993 | 1066 | 1188 | 1092 | 0.874 |
|  | 1 | 1391 | 716 | 861 | 838 | 947 | 980 |  |
|  | 5 | 496 | 270 | 314 | 321 | 317 | 340 |  |
|  | 28 | 37 | 40 | 39 | 31 | 14 | 8 |  |
| Monocyte | 6 | 103 | 440 | 258 | 434 | 264 | 351 | 0.643 |
|  | 9 | 142 | 393 | 209 | 368 | 254 | 363 |  |
|  | 11 | 169 | 263 | 239 | 232 | 214 | 330 |  |
|  | 12 | 112 | 301 | 205 | 321 | 206 | 299 |  |
|  | 13 | 55 | 260 | 471 | 217 | 183 | 191 |  |
|  | 16 | 150 | 221 | 181 | 169 | 281 | 201 |  |
|  | 22 | 174 | 89 | 89 | 87 | 78 | 157 |  |
|  | 24 | 109 | 81 | 80 | 85 | 86 | 97 |  |
|  | 25 | 43 | 102 | 65 | 53 | 80 | 106 |  |
|  | 26 | 96 | 18 | 106 | 104 | 43 | 66 |  |
|  | 27 | 96 | 62 | 45 | 23 | 58 | 66 |  |
| NK cell | 4 | 105 | 310 | 597 | 291 | 155 | 671 | 0.940 |
|  | 7 | 105 | 305 | 453 | 276 | 146 | 558 |  |
|  | 17 | 40 | 117 | 372 | 103 | 65 | 358 |  |
|  | 21 | 27 | 65 | 386 | 45 | 49 | 236 |  |
| pDC | 8 | 141 | 437 | 375 | 452 | 113 | 249 | 0.716 |
|  | 18 | 102 | 199 | 180 | 167 | 209 | 186 |  |
|  | 19 | 151 | 160 | 277 | 104 | 104 | 106 |  |
|  | 20 | 62 | 174 | 163 | 196 | 83 | 208 |  |

BT, BTN3A1; Pri, pristane; pDC, plasmacytoid dendritic cell; NK cell, Natural killer cell.

| Table S6 The amount of T cells corresponding to the samples in each cluster. | | | | | | | |
| --- | --- | --- | --- | --- | --- | --- | --- |
| Cell type | Cell cluster | BT-Pri 1 cell count | BT-Pri 2 cell count | BT-Pri 3 cell count | BT-Pri-IL-38 1 cell count | BT-Pri-IL-38 2 cell count | BT-Pri-IL-38 3 cell count |
| Naive CD4 T cell | 0 | 670 | 261 | 323 | 206 | 413 | 340 |
|  | 7 | 137 | 56 | 61 | 42 | 70 | 66 |
|  | 12 | 7 | 14 | 26 | 9 | 6 | 14 |
| Tfh cell | 1 | 382 | 287 | 230 | 189 | 341 | 311 |
|  | 2 | 451 | 249 | 208 | 147 | 237 | 271 |
| Th1 cell | 3 | 244 | 92 | 127 | 152 | 148 | 138 |
| Naive CD8 T cell | 4 | 279 | 135 | 86 | 72 | 117 | 159 |
|  | 10 | 56 | 24 | 31 | 21 | 32 | 50 |
| CD3e^-^CD8a^+^ T cell | 5 | 139 | 133 | 231 | 114 | 76 | 110 |
| CD3e^-^CD40LG^-^CD8a^+^CCR7^+^ T cell | 6 | 122 | 56 | 91 | 43 | 56 | 85 |
| CD8 Pro GZMK | 8 | 75 | 73 | 52 | 42 | 69 | 73 |
| NK T cell | 9 | 92 | 32 | 44 | 35 | 59 | 68 |
| CD3e^-^CD4^+^GPR183^+^S100A4^+^ T cell | 11 | 23 | 14 | 11 | 5 | 26 | 35 |
| CD3e^-^GATA3^+^MAF^+^CCR4^+^ T cell | 13 | 9 | 8 | 7 | 4 | 4 | 5 |

BT, BTN3A1; Pri, pristane; Tfh, T follicular helper; Th1, T helper 1 ; NK, Natural killer.

| Table S7 The amount of B cells corresponding to the samples in each cluster. | | | | | | | |
| --- | --- | --- | --- | --- | --- | --- | --- |
| Cell type | Cell cluster | BT-Pri 1 cell count | BT-Pri 2 cell count | BT-Pri 3 cell count | BT-Pri-IL-38 1 cell count | BT-Pri-IL-38 2 cell count | BT-Pri-IL-38 3 cell count |
| Follicular B cells | 0 | 1035 | 535 | 525 | 597 | 659 | 567 |
|  | 1 | 857 | 500 | 536 | 560 | 651 | 637 |
|  | 2 | 768 | 336 | 365 | 365 | 499 | 523 |
|  | 8 | 6 | 17 | 16 | 6 | 5 | 7 |
|  | 9 | 10 | 9 | 18 | 7 | 5 | 5 |
| Marginal Zone B cells | 3 | 400 | 260 | 332 | 381 | 291 | 336 |
| Transitional B cells | 4 | 239 | 128 | 130 | 131 | 149 | 134 |
| Plasma cells | 5 | 234 | 100 | 114 | 121 | 132 | 91 |
| B1 cells/Atypical memory B cells | 6 | 105 | 69 | 69 | 51 | 55 | 54 |
| Germinal center-like B cells | 7 | 46 | 29 | 102 | 37 | 20 | 66 |

BT, BTN3A1; Pri, pristane.

| Table S8 The amount of NK cells corresponding to the samples in each cluster. | | | | | | | |
| --- | --- | --- | --- | --- | --- | --- | --- |
| Cell type | Cell cluster | BT-Pri 1 cell count | BT-Pri 2 cell count | BT-Pri 3 cell count | BT-Pri-IL-38 1 cell count | BT-Pri-IL-38 2 cell count | BT-Pri-IL-38 3 cell count |
| Rsrp1^+^Xpo7^+^ NK cell | 0 | 30 | 121 | 357 | 110 | 61 | 384 |
|  | 2 | 44 | 102 | 269 | 94 | 72 | 215 |
| Hbb-bs^+^ NK cell | 1 | 52 | 149 | 217 | 144 | 56 | 296 |
|  | 4 | 16 | 59 | 339 | 37 | 44 | 215 |
| Mik67^+^Ccnb2^+^ NK cell | 3 | 37 | 87 | 155 | 113 | 62 | 268 |
|  | 5 | 27 | 90 | 180 | 80 | 44 | 199 |
|  | 6 | 19 | 47 | 138 | 44 | 29 | 142 |
|  | 7 | 33 | 78 | 69 | 47 | 35 | 63 |
| Gm26917^+^ NKcell | 8 | 19 | 64 | 84 | 46 | 12 | 41 |

BT, BTN3A1; Pri, pristane; NK, natural killer.

| Table S9 The amount of monocytes corresponding to the samples in each cluster. | | | | | | | |
| --- | --- | --- | --- | --- | --- | --- | --- |
| Cell type | Cell cluster | BT-Pri 1 cell count | BT-Pri 2 cell count | BT-Pri 3 cell count | BT-Pri-IL-38 1 cell count | BT-Pri-IL-38 2 cell count | BT-Pri-IL-38 3 cell count |
| Jchain^+^Igkc^+^ monocyte | 0 | 161 | 257 | 203 | 229 | 210 | 308 |
|  | 15 | 7 | 10 | 15 | 8 | 3 | 15 |
| CD11b^+^/Itgam monocyte | 1 | 107 | 285 | 172 | 304 | 197 | 280 |
|  | 2 | 48 | 247 | 137 | 243 | 127 | 242 |
|  | 5 | 50 | 203 | 117 | 206 | 128 | 164 |
|  | 6 | 49 | 225 | 111 | 198 | 124 | 159 |
|  | 14 | 9 | 45 | 34 | 44 | 17 | 41 |
| CD62L^+^/Sell monocyte | 3 | 92 | 210 | 162 | 146 | 218 | 184 |
|  | 7 | 151 | 127 | 65 | 128 | 183 | 118 |
|  | 9 | 152 | 81 | 73 | 71 | 69 | 143 |
| Unknown monocyte (Hba-a1) | 4 | 22 | 113 | 427 | 112 | 104 | 110 |
| CD43^+^/Spn monocyte | 8 | 37 | 156 | 122 | 125 | 90 | 117 |
|  | 12 | 43 | 99 | 65 | 51 | 80 | 104 |
| CD11c^+^/Itgax monocyte | 10 | 120 | 88 | 86 | 94 | 89 | 103 |
|  | 13 | 95 | 60 | 45 | 19 | 61 | 65 |
| CD115^+^CD43^+^ monocyte | 11 | 106 | 24 | 114 | 115 | 47 | 74 |

BT, BTN3A1; Pri, pristane.

| Table S10 The amount of pDC cells corresponding to the samples in each cluster. | | | | | | | |
| --- | --- | --- | --- | --- | --- | --- | --- |
| Cell type | Cell cluster | BT-Pri 1 cell count | BT-Pri 2 cell count | BT-Pri 3 cell count | BT-Pri-IL-38 1 cell count | BT-Pri-IL-38 2 cell count | BT-Pri-IL-38 3 cell count |
| Mt1^+^ pDC | 0 | 64 | 196 | 107 | 180 | 58 | 106 |
|  | 3 | 25 | 123 | 133 | 161 | 18 | 47 |
|  | 10 | 22 | 31 | 28 | 36 | 15 | 27 |
| Tmem14c^+^ pDC | 1 | 65 | 140 | 126 | 150 | 58 | 149 |
|  | 2 | 37 | 118 | 91 | 128 | 57 | 133 |
| Pf4^+^ pDC | 4 | 49 | 62 | 100 | 74 | 108 | 88 |
| Ebf1^+^ pDC | 5 | 91 | 88 | 73 | 69 | 55 | 55 |
| Lyz2^+^ pDC | 6 | 33 | 108 | 57 | 69 | 87 | 69 |
| H2-Ab1^+^ pDC | 7 | 37 | 35 | 112 | 14 | 13 | 10 |
| Jchain^+^ pDC | 8 | 29 | 46 | 28 | 21 | 36 | 44 |
| S100a9^+^ pDC | 9 | 0 | 22 | 136 | 13 | 3 | 18 |
| Igfbp7^+^ pDC | 11 | 4 | 1 | 4 | 4 | 1 | 3 |

BT, BTN3A1; Pri, pristane; pDC, plasmacytoid dendritic cell.

| Table S11 Characteristics of SLE patients and healthy controls. | | | |
| --- | --- | --- | --- |
| Characteristics | SLE patients | Healthy controls | P value |
| Male/Female (n) | 32/248 | 12/88 | 0.917 |
| Age (year) | 39 (12-77) | 42 (20-60) | 0.584 |
| SLEDAI | 10 (6, 14.5) | - | - |
| Mental disorder (+) (n) | 4 | - | - |
| Optic nerve degeneration (+) (n) | 10 | - | - |
| Lupus headache (+) (n) | 20 | - | - |
| Vasculitis (+) (n) | 22 | - | - |
| Arthritis (+) (n) | 153 | - | - |
| Myositis (+) (n) | 40 | - | - |
| Cylindruria (+) (n) | 15 | - | - |
| Hematuria (+) (n) | 77 | - | - |
| Lupus nephritis (+) (n) | 123 | - | - |
| Pyuria (+) (n) | 32 | - | - |
| Rash (+) (n) | 89 | - | - |
| Alopecia (+) (n) | 69 | - | - |
| Mucosal ulcer (+) (n) | 22 | - | - |
| Low complement (+) (n) | 110 | - | - |
| Fever (+) (n) | 55 | - | - |
| Thrombocytopenia (+) (n) | 50 | - | - |
| Leukocytopia (+) (n) | 34 | - | - |
| ANA (+) (n) | 145 | - | - |
| anti-dsDNA (+) (n) | 53 | - | - |
| anti-Sm (+) (n) | 59 | - | - |
| anti-SSA (+) (n) | 102 | - | - |
| anti-SSB (+) (n) | 34 | - | - |
| anti-RNF (+) (n) | 49 | - | - |
| C3 (g/L) | 0.77±0.34 | - | - |
| C4 (g/L) | 0.17±0.11 | - | - |
| IgA (g/L) | 2.92±1.86 | - | - |
| IgG (g/L) | 14.69±6.53 | - | - |
| RF (IU/mL) | 11.71±8.48 | - | - |
| CRP (mg/L) | 17.48±35.12 | - | - |
| ESR (mm/H) | 32.50±29.23 | - | - |

SLE, systemic lupus erythematosus; SLEDAI, systemic lupus erythematosus disease activity index; RF, rheumatoid factor; CRP, C-reactive protein; ANA, antinuclear antibody; ESR, erythrocyte sedimentation rate.

| Table S12 Characteristics of SLE patients in qRT-PCR and flow cytometry analysis. | | | | | | | | | | | | |
| --- | --- | --- | --- | --- | --- | --- | --- | --- | --- | --- | --- | --- |
| qRT-PCR analysis | | | | | |  | Flow cytometry analysis | | | | | |
|  | Age | Gender |  | Age | Gender |  |  | Age | Gender |  | Age | Gender |
| SLE-1 | 44 | Female | HC-1 | 18 | Female |  | SLE-31 | 24 | Female | HC-31 | 24 | Female |
| SLE-2 | 47 | Female | HC-2 | 36 | Female |  | SLE-32 | 64 | Female | HC-32 | 25 | Female |
| SLE-3 | 47 | Female | HC-3 | 39 | Female |  | SLE-33 | 53 | Female | HC-33 | 27 | Female |
| SLE-4 | 36 | Female | HC-4 | 31 | Female |  | SLE-34 | 54 | Female | HC-34 | 51 | Male |
| SLE-5 | 54 | Female | HC-5 | 57 | Female |  | SLE-35 | 50 | Male | HC-35 | 52 | Male |
| SLE-6 | 62 | Male | HC-6 | 60 | Female |  | SLE-36 | 27 | Female | HC-36 | 65 | Male |
| SLE-7 | 52 | Female | HC-7 | 48 | Female |  | SLE-37 | 24 | Female | HC-37 | 61 | Male |
| SLE-8 | 54 | Female | HC-8 | 24 | Male |  | SLE-38 | 21 | Female | HC-38 | 25 | Female |
| SLE-9 | 36 | Female | HC-9 | 43 | Female |  | SLE-39 | 66 | Male | HC-39 | 53 | Female |
| SLE-10 | 38 | Female | HC-10 | 36 | Female |  | SLE-40 | 46 | Female | HC-40 | 36 | Female |
| SLE-11 | 22 | Female | HC-11 | 41 | Female |  | SLE-41 | 52 | Male | HC-41 | 43 | Female |
| SLE-12 | 43 | Female | HC-12 | 51 | Female |  | SLE-42 | 44 | Female | HC-42 | 85 | Female |
| SLE-13 | 66 | Female | HC-13 | 36 | Female |  | SLE-43 | 37 | Female | HC-43 | 49 | Female |
| SLE-14 | 59 | Female | HC-14 | 53 | Female |  | SLE-44 | 18 | Female | HC-44 | 22 | Female |
| SLE-15 | 26 | Female | HC-15 | 55 | Female |  | SLE-45 | 59 | Male | HC-45 | 21 | Female |
| SLE-16 | 60 | Female | HC-16 | 25 | Female |  | SLE-46 | 27 | Female | HC-46 | 54 | Female |
| SLE-17 | 52 | Female | HC-17 | 50 | Female |  | SLE-47 | 46 | Female | HC-47 | 18 | Female |
| SLE-18 | 24 | Female | HC-18 | 25 | Female |  | SLE-48 | 21 | Female | HC-48 | 26 | Female |
| SLE-19 | 44 | Female | HC-19 | 41 | Female |  |  |  |  |  |  |  |
| SLE-20 | 18 | Female | HC-20 | 52 | Female |  |  |  |  |  |  |  |
| SLE-21 | 52 | Female | HC-21 | 54 | Female |  |  |  |  |  |  |  |
| SLE-22 | 52 | Male | HC-22 | 35 | Female |  |  |  |  |  |  |  |
| SLE-23 | 58 | Female | HC-23 | 47 | Female |  |  |  |  |  |  |  |
| SLE-24 | 58 | Female | HC-24 | 59 | Female |  |  |  |  |  |  |  |
| SLE-25 | 51 | Female | HC-25 | 24 | Female |  |  |  |  |  |  |  |
| SLE-26 | 56 | Male | HC-26 | 66 | Female |  |  |  |  |  |  |  |
| SLE-27 | 56 | Female | HC-27 | 56 | Female |  |  |  |  |  |  |  |
| SLE-28 | 34 | Female | HC-28 | 52 | Female |  |  |  |  |  |  |  |
| SLE-29 | 30 | Female | HC-29 | 32 | Female |  |  |  |  |  |  |  |
| SLE-30 | 36 | Female | HC-30 | 75 | Female |  |  |  |  |  |  |  |

SLE, systemic lupus erythematosus; HC, healthy control.

Table S13 Scoring criteria for pathologic grading of renal injury.

| Type | Lesion level | Grading |
| --- | --- | --- |
| Glomerular injury/necrosis | No, <5% | 0 |
|  | Mild, 5%-25% | 1 |
|  | Moderate, 26%-50% | 2 |
|  | Severe, >50% | 3 |
| Renal tubular injury/necrosis | No, <5% | 0 |
|  | Mild, 5%-25% | 1 |
|  | Moderate, 26%-50% | 2 |
|  | Severe, >50% | 3 |
| Interstitial inflammation of the kidney | No, <5% | 0 |
|  | Mild, 5%-25% | 1 |
|  | Moderate, 26%-50% | 2 |
|  | Severe, >50% | 3 |
| Renal interstitial fibrosis | No, <5% | 0 |
|  | Mild, 5%-25% | 1 |
|  | Moderate, 26%-50% | 2 |
|  | Severe, >50% | 3 |
| Protein cast | No, <5% | 0 |
|  | Mild, 5%-25% | 1 |
|  | Moderate, 26%-50% | 2 |
|  | Severe, >50% | 3 |

| Table S14 Primer sequences for related genes. | | |
| --- | --- | --- |
| Gene | Sequences | |
|  | Mouse | Human |
| Steap3 | Former: GAGGTCATCTTTGTGGCCGT  Reverse: TTCTCCGTGGGGTTGCTTAC | Former: AGGTCATCTTTGTGGCTGTGTTC  Reverse: GCTGAAGGTGCTCTTGCTCTG |
| Slc11a2 | Former: CCTGTGGCTGATGGTGGAGTTG  Reverse:AAGTGTCTGCGATGGTGATGAGG | Former: TCCCTTTGCTCTCATACCCATCC  Reverse: ATCCGCCAGCCTAGTCCATTG |
| Trfc | Former: AGCAGCATTGGTCAAAACATGG  Reverse: ATGCCACATAACCCTCGGGA | Former: GACTATGAGAGGTACAACAGCCAAC  Reverse: GAAGAAGTCTCCACGAGCAGAATAC |
| Slc7a11 | Former: GTTCGCTGTCTCCAGGTTATTCTAC  Reverse: AGAGCATCACCATCGTCAGAGG | Former: TTTTCTGAGCGGCTACTGGG  Reverse: CAGCAAACACACCACCGTTC |
| GAPDH | Former: GGTTGTCTCCTGCGACTTCA  Reverse: TGGTCCAGGGTTTCTTACTCC | Former: CAGGAGGCATTGCTGATGAT  Reverse: GAAGGCTGGGGCTCATTT |
